# Mental Health Screening Approaches for Resettling Refugees and Asylum Seekers: A Scoping Review

**DOI:** 10.3390/ijerph19063549

**Published:** 2022-03-16

**Authors:** Olivia Magwood, Azaad Kassam, Dorsa Mavedatnia, Oreen Mendonca, Ammar Saad, Hafsa Hasan, Maria Madana, Dominique Ranger, Yvonne Tan, Kevin Pottie

**Affiliations:** 1 C.T. Lamont Primary Care Research Center, Bruyère Research Institute, 85 Primrose Avenue, Ottawa, ON K1R 7G5, Canada; omagwood@bruyere.org (O.M.); oreenmend@gmail.com (O.M.); ammar.saad@uottawa.ca (A.S.); hafsa.hsnn@gmail.com (H.H.); dominiqueranger.730@gmail.com (D.R.); yvonnetann@yahoo.com (Y.T.); 2 Interdisciplinary School of Health Sciences, Faculty of Health Sciences, University of Ottawa, 25 University Private, Ottawa, ON K1N 7K4, Canada; 3 Department of Psychiatry, University of Ottawa, 75 Laurier Ave E, Ottawa, ON K1N 6N5, Canada; a.kassam@pqchc.com; 4 Pinecrest-Queensway Community Health Centre, 1365 Richmond Rd #2, Ottawa, ON K2B 6R7, Canada; 5 Ottawa Newcomer Health Centre, 291 Argyle, Ottawa, ON K2P 1B8, Canada; 6 Faculty of Medicine, University of Ottawa, 451 Smyth Road, Ottawa, ON K1H 8M5, Canada; dmave060@uottawa.ca (D.M.); mmada042@uottawa.ca (M.M.); 7 School of Epidemiology and Public Health, University of Ottawa, 600 Peter Morand Crescent, Ottawa, ON K1G 5Z3, Canada; 8 Children’s Hospital of Eastern Ontario Research Institute, Ottawa, ON K1H 5B2, Canada; 9 Institute of Health Policy, Management and Evaluation, University of Toronto, 155 College St, Toronto, ON M5T 3M6, Canada; 10 Faculty of Arts and Sciences, Queen’s University, 99 University Ave, Kingston, ON K7L 3N6, Canada; 11 Department of Family Medicine, Western University, London, ON N6A 3K7, Canada

**Keywords:** refugee, asylum seeker, mental health, resettlement, migration, screening, health assessment

## Abstract

Refugees and asylum seekers often face delayed mental health diagnoses, treatment, and care. COVID-19 has exacerbated these issues. Delays in diagnosis and care can reduce the impact of resettlement services and may lead to poor long-term outcomes. This scoping review aims to characterize studies that report on mental health screening for resettling refugees and asylum seekers pre-departure and post-arrival to a resettlement state. We systematically searched six bibliographic databases for articles published between 1995 and 2020 and conducted a grey literature search. We included publications that evaluated early mental health screening approaches for refugees of all ages. Our search identified 25,862 citations and 70 met the full eligibility criteria. We included 45 publications that described mental health screening programs, 25 screening tool validation studies, and we characterized 85 mental health screening tools. Two grey literature reports described pre-departure mental health screening. Among the included publications, three reported on two programs for women, 11 reported on programs for children and adolescents, and four reported on approaches for survivors of torture. Programs most frequently screened for overall mental health, PTSD, and depression. Important considerations that emerged from the literature include cultural and psychological safety to prevent re-traumatization and digital tools to offer more private and accessible self-assessments.

## 1. Introduction

Approximately 79.5 million people around the world have been forced to leave their homes, and nearly 26 million are considered refugees [1]. The COVID-19 pandemic has also created unprecedented delays in resettlement [2]. In 2022, a projected 1.47 million refugees will need urgent resettlement [3]. ”Resettlement” is the selection and transfer of refugees from a state in which they have sought temporary protection to a third state that has agreed to admit them as refugees with permanent residence status [4]. This status ensures protection against refoulement and provides a resettled refugee and their family or dependents with access to civil, political, economic, social, and cultural rights [1]. Conversely, an asylum seeker is someone whose claim for protection and resettlement has not yet been finally decided on by the country in which the claim is submitted [5].

Providing refugees and asylum seekers appropriate and timely mental health services is a global challenge [6]. Most recently, the COVID-19 pandemic has dramatically reduced programs and delayed refugee resettlement, thereby increasing uncertainty and isolation [2,7]. Early screening and care for common mental health disorders is now recognized as a priority for resettlement programs [8,9]. However, there is a risk that screening for mental health can lead to re-traumatization [10]. Therefore, screening approaches should incorporate safety, comfort, physical care, ensure access to basic needs, and use culturally appropriate tools and clinical assessments [11].

Refugees encounter many risk factors for poor mental health outcomes before, during, and after migration and resettlement [6]. Such factors include exposure to traumatic violence, genocide, and economic hardship; experience of physical harm and separation; and poor socioeconomic conditions once resettled, such as social isolation, racism, and unemployment [12,13]. Refugees are at risk of developing common mental health disorders including depression, anxiety, posttraumatic stress disorder (PTSD), and related somatic health symptoms [6,14,15]. Epidemiological studies indicate that the age-standardized point prevalence of PTSD and major depression in conflict-affected populations is estimated to be 12.9% and 7.6%, respectively [16]. As a comparison, it has been estimated that approximately 4.4% of the world’s population suffers from major depression [17] and 3.3% from PTSD [18]. However, the true prevalence of common mental disorders among refugees could be higher since there is no systematic or consistent approach to diagnose mental disorders in this population [12].

Pre-departure overseas health assessments and screening represent a potential, but often underdeveloped, component of the migration health screening process for resettling refugees. A health assessment is a medical examination, usually conducted by a registered medical practitioner (or “panel physician”) based on criteria set by the resettlement state [19]. Health assessments are conducted as a measure to limit or prevent the transmission of diseases of public health importance to their host populations and to avert potential costs and burdens on local health systems [19]. These assessments support the health of migrating populations as well as protect domestic public health, promote collaboration with international health partners, and strengthen understanding of the health profiles of diverse arriving populations [20]. For example, health assessment results may be communicated to local resettlement agencies so that appropriate health services can be arranged for the refugees on arrival [21]. However, if refugee health assessment processors are to meaningfully contribute to the public health good, then they need to overcome exclusionary approaches, be linked to the national health systems, and be complemented by health promotion measures to enhance the health-seeking behaviour of refugees [19]. Currently, there are 24 official resettlement states (See Supplementary File S1) for whom pre-departure mental health screening approaches for refugees could be beneficial.

Current health assessments do not routinely screen for common mental health concerns. Providing early care for treatable mental health conditions could help refugees benefit from resettlement, language, cultural and employment training programs, develop positive relationships, reduce intergenerational trauma, gain access to employment, and ultimately lead to more meaningful and productive lives [8]. Developing early common mental health screening and treatment programs is therefore an important first step when integrating refugees into local primary healthcare services [22].

The majority of synthesized literature on refugee mental health to date focuses on the prevalence of mental illness (for example, [23,24,25]); access to mental health services (for example, [26,27]); and tailored programs and interventions (for example, [28,29,30]). There is limited available evidence which characterizes screening tools and procedures specific to assessing mental health among refugee and asylum-seeking populations during resettlement. One existing systematic review identified only seven screening tools for trauma and mental health assessment in refugee children [31]. Older reviews suggest that more tools have been used among adult populations; however, the authors concluded that existing tools had limited or untested validity and reliability in refugees [32,33].

## 2. Research Objectives

The objective of this scoping review is to identify and characterize mental health screening approaches for refugees and asylum seekers. This review aims to inform and catalyze country-level resettlement policies and practices regarding the identification of mental health conditions and linkage to care by addressing the following research question:

What are the characteristics of existing and emerging approaches to mental health screening for resettling refugees and asylum seekers? (See Box 1).

Box 1. **Research sub-questions**

➢ In what setting(s) has refugee mental health screening been conducted?
➢ At what point in time during the migration pathway is screening conducted and for what purpose?
➢ What tools have been used in the refugee population, and what conditions do they screen for?
➢ In which language(s) and formats are mental health assessments delivered?
➢ Have any of these tools been adapted, validated, or evaluated specifically for use among refugees?
➢ What approaches are used to screen vulnerable subgroups?
➢ What are the professional characteristics and training of individuals who administer mental health assessments?
➢ What are the lessons learned from pilots/approaches that have been tried on the ground?

## 3. Methods

**Figure 1:**
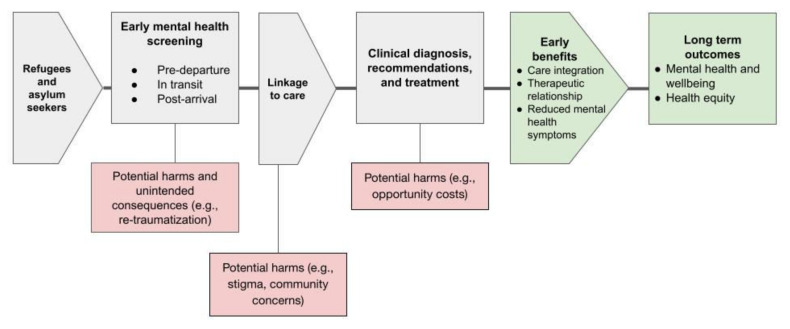
Logic model of mental health screening along the resettlement pathway.

We registered the methods of this scoping review on the Open Science Framework (DOI: 10.17605/OSF.IO/RWVBE) and published an open-access protocol [34]. We reported our review according to the PRISMA extension for scoping reviews (PRISMA-ScR; [35]) [Supplementary File S2]. We reported our search strategy according to the PRISMA Statement for Reporting Literature Searches in Systematic Reviews (PRISMA-S; [36]) [Supplementary File S3]. We created a logic model to outline the conceptual framework involved in the mental health screening process (Figure 1).

## 4. Eligibility Criteria

We identified eligible studies using the SPIDER acronym (Table 1). We included publications of quantitative, qualitative, or mixed-methods evidence that evaluated approaches to the early screening of mental health disorders among resettling refugees and asylum seekers of all ages. We defined the resettling period as 6 months prior to travel and 12 months after arrival in the resettlement country. We excluded qualitative publications that focused on patient experiences rather than characteristics of early screening approaches. By “approach”, we mean the process from the assessment of mental health to the transfer of results to the patient, immigration officials, or healthcare providers, including the development of the assessment tool itself if it included pilot-testing and validation among refugees. We considered documents published in any language. We restricted the year of publication from 1995 to 2020 to coincide with the creation of the Annual Tripartite Consultations on Resettlement (ATCR) and subsequent UNHCR Resettlement Handbook [37].

## 5. Search Methods

We developed our search strategies in consultation with a health sciences librarian. We searched the following databases, individually, from 1995 to 2020: EMBASE (Ovid; 1995 to 24 December 2020); Medline (Ovid; 1995 to 21 December 2020); PsycINFO (Ovid; 1995 to December Week 3 2020); Cochrane Central Register of Controlled Trials (CENTRAL) (Ovid; 1995 to January Week 2 2021); Cumulative Index to Nursing and Allied Health Literature (CINAHL) (Ebsco; January 1995 to January Week 2 2021). We used a combination of keywords and subject headings. Complete search strategies for each database are available in Supplementary File S4.

In addition to searching bibliographic databases, we conducted a focused grey literature search. We searched the government websites from the 24 countries listed in Supplementary File S1 and the International Organization for Migration (IOM). We contacted an immigration policy researcher from each country of the Immigration and Refugee Health Working Group (Australia, Canada, New Zealand, United Kingdom, United States of America) and other experts to identify any missing literature.

## 6. Screening and Selection

We used Covidence software [38] and a two-part study selection process: (1) a title and abstract review, and (2) full-text review. Two review authors independently assessed all potential studies and documents against a priori inclusion and exclusion criteria (Table 1). We resolved any disagreements through discussion, or we consulted a third review author.

## 7. Data Extraction and Management

We developed a standardized extraction sheet. Pairs of reviewers extracted data in duplicate and independently. They compared results and resolved disagreements by discussion or with help from a third reviewer. To ensure the validity of the data extraction form, we piloted this form with two reviewers and the accuracy of the content was confirmed with a third reviewer. Reviewers extracted all variables identified in our protocol [34].

## 8. Synthesis of Results

We summarized the data according to the setting, timing, and purpose of the assessment, as well as the characteristics of screening tools and administrators. We narratively described approaches for special populations and implementation lessons learned, as described by the study authors. As a scoping review, the purpose of this study is to present an overview of the research rather than to evaluate the quality of the individual studies; therefore, we did not conduct an overall assessment or appraisal of the strength of the evidence.

## 9. Results

**Figure 2:**
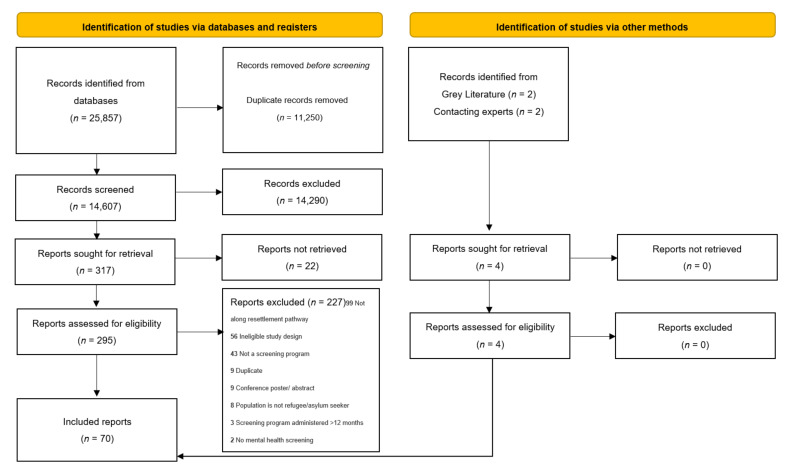
PRISMA Flow Diagram.

Our systematic search identified 25,857 citations. After the removal of duplicates, we screened 14,607 citations by title and abstract. We retained 315 for full text review. Of these, 66 met full eligibility criteria. Reasons for exclusion are presented in Figure 2 and Supplementary File S5. Additionally, our grey literature search identified two additional publications for inclusion. Two studies were brought forward by immigration representatives and other subject matter experts, for a total of 70 included studies.

## 10. Characteristics of Included Studies

**Figure 3:**
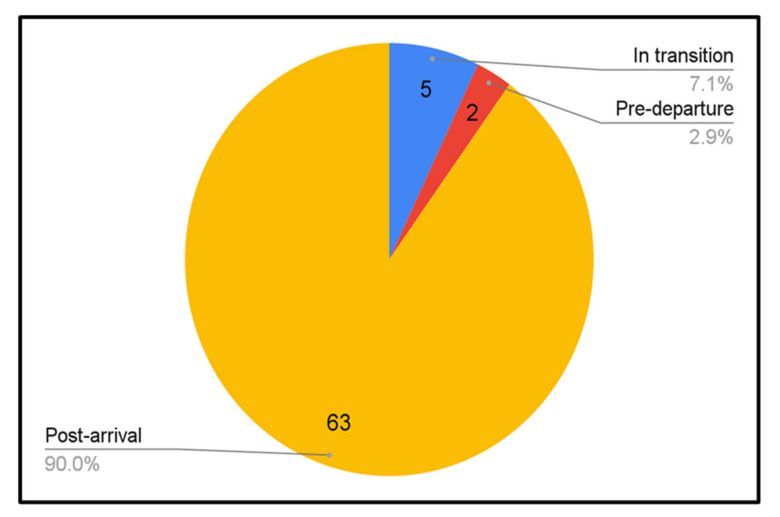
Timing of mental health assessments.

**Figure 4:**
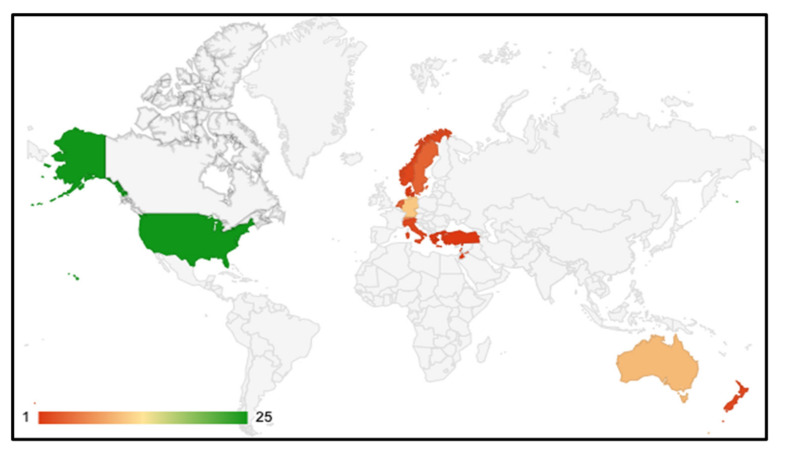
Global distribution of mental health assessments for refugees and asylum seekers according to setting of screening. To note: we identified one publication on pre-departure screening conducted in Lebanon prior to departure to the UK.

**Figure 5:**
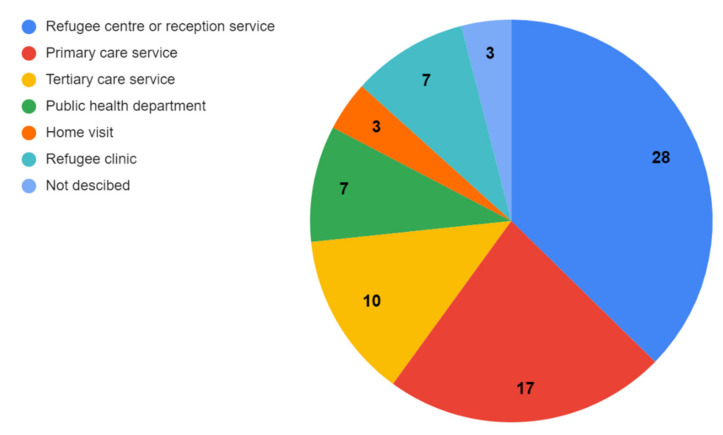
Setting of mental health assessments.

We summarized the characteristics of all included publications of refugee mental health screening approaches (see Table 2). This included 45 publications which described screening programs, and 25 validation studies conducted in 13 different resettlement countries. Most assessments (90% of included studies) occurred within the first 12 months post-arrival to the resettlement country (see Figure 3). We identified two reports of pre-departure screening prior to resettlement [4,39]. Post-arrival assessments were most common in the USA, Switzerland, and Australia (See Figure 4). While some assessments were conducted in tertiary care settings (i.e., hospitals), most refugees sought health assessments in primary care clinics or interdisciplinary refugee health clinics (see Figure 5). Assessments were also held in community or public health centres or other settings such as detention centres, national intermediary centres, independent medical examinations, and torture treatment centres. One study reported that mental health screening was most effective when completed during a home visit [40]. Among publications which included asylum seeker populations (18/70), the majority conducted screening at reception centres and asylum accommodations.

## 11. Conditions and Mental Health Screening Tools

**Figure 6:**
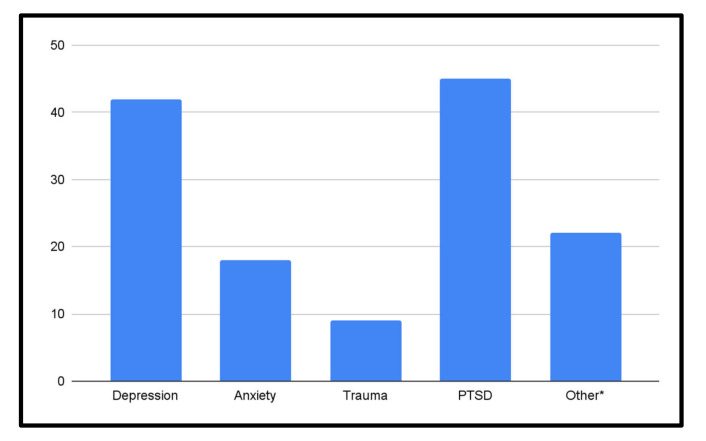
Overview of mental health conditions assessed among refugees and asylum seekers. * NB: Any mental health assessments that did not include depression, anxiety, trauma, or PTSD were categorized as ‘Other’ (e.g., general mental health, panic disorders, adverse childhood events, etc.)

A total of 85 mental health screening tools were identified (Table 3). Several of these tools are available in multiple languages and are either self-administered or administered by various trained professionals such as primary care providers (PCPs), mental health specialists (MHSs), or community health workers (CHWs). Several tools could also be administered by a lay person without clinical training [46,64,80]. The most common tools were the Harvard Trauma Questionnaire (HTQ), the Hopkins Symptom Checklist-25 (HSCL-25), the Mini International Neuropsychiatric Interview (MINI), and the Refugee Health Screener-15 (RHS-15). The most common screened mental health conditions were overall mental health, PTSD, depression, and anxiety (see Figure 6).

## 12. Pre-Departure Mental Health Screening

The International Organization for Migration (IOM) conducts several pre-migration health activities at the request of receiving country governments to identify health conditions of public health importance and to provide continuity of care linking the pre-departure, travel, and post-arrival phases. These assessments include radiology services, laboratory services, treatment of communicable diseases, vaccinations, and detection of non-communicable diseases, including mental health assessments. We identified two grey literature reports which evaluated pre-departure screening programs for refugees [4,39].

In 2019, IOM conducted a total of 110,992 pre-departure health assessments for refugees [4]. Most assessments among refugees were conducted in Lebanon (11.7%), Turkey (11.1%), and Jordan (8.8%). The top destination countries were the United States (39.7%), Canada (27.9%), and Australia (14.6%). In total, 48.8 percent of assessments were conducted among females and 51.2 percent among males. The majority of health assessments were among refugees younger than 30 (67.1%), with the highest number in the under-10 age group. During 2019, mental health conditions were identified in 2249 pre-departure health assessments conducted among refugees (2.0%). Where indicated, refugees were referred to a specialist for further evaluation (1%). The report does not provide any further details on the specific conditions assessed or other administration details [4].

In 2016–2017, IOM collaborated with Public Health England (UK) to evaluate the pre-departure administration of the Global Mental Health Assessment Tool (GMHAT) among 200 Syrian refugees in a refugee camp in Lebanon [39]. These refugees had already been accepted for resettlement to the UK. This clinically validated, computerized assessment tool was administered by a range of healthcare staff and was designed to detect common psychiatric disorders and serious mental health conditions within the time span of 15–20 min [39].

Findings suggested that a pre-departure mental health assessment could be a useful tool to assist in the preparation for refugee arrivals to overseas resettlement facilities and serve as a valuable resource for general practitioners. Other potential benefits included overcoming barriers such as trust and language, expediting referral and treatment, increasing awareness of mental health issues, and improving support and integration of refugees by proactively addressing concerns [39].

Several considerations were identified to improve the impact and roll out of pre-departure mental health assessments [39]. Firstly, the GMHAT identified 9% of participants with a likely diagnosis of mental illness and an additional 1.5% of participants were referred post-arrival based on clinical judgment; as such, it was noted that the pre-arrival assessment should not be used in isolation or as a replacement for routine psychological assessments post-arrival, and that practitioners should use their clinical expertise to pick up on any missed diagnoses. Secondly, the use of the tool was deemed appropriate, but it was noted that participants’ cases took longer to process than those who had not undergone an assessment. Though it was not possible to distinguish whether the GMHAT was the cause of the delay, this is an important consideration. An evaluation of the program indicated that additional information is needed to estimate the impacts on costs and case processing times. Further, the authors concluded it is important to ensure that the information obtained from the pre-assessment will not lead to the rejection of vulnerable refugees based on their mental health status nor based on the resettlement country’s service availability. Clear parameters should be defined to determine the flow of information sharing, and usage should be defined a priori, as it was noted that some healthcare workers in this pilot study were unsure on how the information was intended to be used and whom it could be shared with, ultimately devaluing the purpose of this tool. Lastly, it is important to ensure adequate post-arrival mental health service delivery, since pre-departure assessments can also pose a risk of raising expectations of the care that refugees hope to receive upon resettlement. Although not unique to pre-departure screening procedures, several other concerns were raised including the risk of re-traumatization during assessments, the increased need for mental health services upon arrival, additional guidance and training for healthcare workers, and an increase in the provision of culturally appropriate services. This pilot study acknowledges concerns regarding the acceptability of screening for refugees but recommends that the GHMAT tool requires further modifications to be appropriate for use in the resettlement context [39].

## 13. Mental Health Screening for Survivors of Torture

We identified four studies which described screening approaches and tools for survivors of torture [45,78,80,105]. Masmas and colleagues identified a high prevalence of torture survivors among an unselected population of asylum seekers using the WHO’s General Health Questionnaire and a clinical interview conducted by physicians [78]. Mewes and colleagues conducted a validation study among adult asylum seekers in Germany. They used the Process of Recognition and Orientation of Torture Victims in European Countries to Facilitate Care and Treatment (PROTECT) Questionnaire, which identifies symptoms of PTSD and depression and categorizes asylum seekers into risk categories, supporting a two-stage approach to mental health screening [80]. The questionnaire was specially developed to be administered by nonmedical/psychological staff for the early identification of asylum seekers who suffered traumatic experiences (e.g., experiences of torture). The tool was administered directly in refugee reception centres and refugee accommodations. The validity of the PROTECT Questionnaire was confirmed by Wulfes and colleagues, who concluded that the use of the PROTECT Questionnaire could be more efficient than other brief screening tools (eight-item short-form Posttraumatic Diagnostic Scale (PDS-8) and the Patient Health Questionnaire (PHQ-9)) because it detects two conditions at once [105].

Among included studies, most community-based programs were not offered specifically for victims of torture. In New York, USA, a hospital-based Program for Survivors of Torture (PSOT) exists to offer services to clients who experienced torture [45]. Referrals to this program typically came from asylum lawyers, other health care professionals when they learned about these clients’ trauma histories, and word-of-mouth in the communities. At PSOT, asylum seekers were screened for PTSD with the Harvard Trauma Questionnaire (HTQ) and Major Depressive Disorder (MDD) screening was conducted with the Patient Health Questionnaire-9 (PHQ-9). If a client screened positive for MDD or PTSD at intake, they were referred for a mental health evaluation and management through PSOT. Severe cases were evaluated urgently by a PSOT psychologist or psychiatrist. Of those clients diagnosed with depression and PTSD, 94% received follow-up, defined as either referral to a psychiatrist, psychologist, or support group, or pharmacologic management by a primary care provider [45]. The high follow-up rate was attributed to the unique multidisciplinary medical home structure of the program, which has significantly more allied health professionals, live interpreters, and support staff than an average primary care clinic in the area [45].

## 14. Mental Health Screening Approaches for Refugee Women

Three publications described two mental health screening programs specifically for refugee women of reproductive age [47,69,104]. The report by Boyle and colleagues is a protocol for a screening program [47] whose acceptability and feasibility has been evaluated [104], but whose effectiveness (outcome) data are not yet available. Boyle et al. conducted their study in a Refugee Antenatal Clinic in Australia [47], while Johnson-Agbakwu et al. conducted their study in a Refugee Women’s Health Clinic in the United States [69]. Both studies screened for mental health conditions post-arrival in a clinic specifically aimed at assessing and treating refugee women. Boyle et al. screened pregnant women in the perinatal and postnatal period at their first appointment, with screening repeated in the third trimester [47]. Johnson-Agbakwu et al. recruited women seeking obstetric and/or gynaecological care, not differentiating between pregnant and non-pregnant women [69]. The purpose of the screening programs was to improve resettlement and integration outcomes [69], and to identify the urgent needs of refugee women for referral to ensure continuity of care [47].

In the USA, Johnson-Agbakwu et al. administered the Refugee Health Screener-15 (RHS-15) with a cultural health navigator to screen women for PTSD, depression, and anxiety [69]. The aim was for women to complete the screening independently and confidentially without the presence of spouses, family members, or friends, as this may influence patient responses. However, this was difficult to enforce. In contrast, Boyle et al. have planned to use the Edinburgh Postnatal Depression Scale to assess depression and anxiety in the perinatal period [47]. In addition, Boyle et al. will use the Monash Health psychosocial needs assessment tool to assess perinatal mental health disorders, such as past birthing experiences, violence and safety, and social factors (finances and housing). Women will complete both assessments on a tablet in their chosen language and interpreters or bicultural workers are available to assist.

The Refugee Women’s Health Clinic where Johnson-Agbakwi et al. conducted their study employed multilingual cultural health navigators; program managers skilled in social work who reflected the ethnic and cultural diversity of the patient population and helped with the administration of the screening tool [69]. They were all female, which helped to build strong rapport and trusting relationships for refugee women to feel more comfortable discussing sensitive concerns in their native language. The implementation of their program was dependent on a community-partnered approach and a sustainable interdisciplinary model of care, which was necessary to build trust, empower refugees towards greater receptivity to mental health services, and provide bi-directional learning. Johnson-Agbakwu et al. reported that interdisciplinary models of care, gender-matched multi-disciplinary health care providers, and patient health navigators and interpreters are necessary for integrated approaches and community empowerment [69].

Prior to the implementation of a screening program in Australia, little support was offered to refugee women as midwives were unsure of what services were available [104]. Following the implementation, midwives expressed they were now making more referrals using a co-designed referral pathway than before the screening program, and more information was available at the point of referral because of screening [104]. Finally, it was reported in the USA that while screening for mental health disorders amongst refugee women provides greater awareness and identifies those who need treatment, many women still do not enroll in mental health care [69]. This was either due to women declining care or a lack of health insurance [69]. It was speculated that one reason women may decline care is due to the social stigma of mental health which could be introduced via social desirability bias and may be heightened through the verbal administration of questionnaires [69].

## 15. Mental Health Screening Approaches for Refugee Children and Adolescents

Eleven studies were identified that investigated mental health screening approaches specific to refugee children and adolescents [52,57,58,59,61,62,67,82,89,97,100]. Children and adolescents between the ages of 6 months to 18 years old were included and all identified screening programs were completed post-arrival to the resettlement country. All studies included adolescent populations (ages 10–18) and fewer studies included children below the age of 10 [58,59,82,89,100]. The programs reported that there is variability in the timing of presentations of mental health disorders; thus, an early assessment of the psychological needs of children and families allows for timely targeted support [58,59].

Children and adolescent screening programs focused on a wider range of conditions which consider critical developmental stages. The psychological factors screened for included: emotional problems, conduct problems, hyperactivity, peer problems and prosocial behavior, stressful life events, PTSD [82,100], anxiety, depression [58,59,67,97], and somatization disorder [58]. Health risk behaviours, health-related quality of life, and physical and psychosocial well-being, including physical functioning, body pain, emotional problems, self-esteem, and family cohesion were also screened for [57,62]. The most common mental health condition screened for was PTSD, as 10/11 identified studies included a questionnaire which screened for it.

Various “child-centered” approaches were described. Two studies, consistent with trauma-informed care guidelines, offered children the possibility to be accompanied by a person they trusted as support [82,100]. In contrast, another study recommended seeing adolescents alone during consultations [62]. Children, regardless of their age, were offered help to read the items on the questionnaire, to clarify and ensure understanding of the concepts being screened for in the questionnaire [89]. When administering questionnaires to children, investigators noted that it is important to not overload them with various instruments as it may cause confusion and a decrease in completion rates [57]. Furthermore, children can experience difficulties with Likert scales and question formats, despite surveys being constructed with attention to literacy, linguistic, and culture issues [57]. The approaches emphasized the importance of interdisciplinary collaboration and discussions regarding confidentiality [59]. Children and adolescents often require diverse services; thus, multidisciplinary healthcare was recommended to manage health risk behaviours (e.g., medical, sexual, reproductive, mental, social services) [59,62].

Only two publications reported on the digital administration of mental health screening with adolescents. Of note, Jakobsen et al. utilized a computer-based system (laptops and touch-screen function) to administer their screening questionnaires to unaccompanied adolescents with limited school backgrounds [67]. Similarly, Sukale et al. administered a computer-based tool named ‘Providing Online Resource and Trauma Assessment’ (PORTA), which combines disorder-specific questionnaires on the topics of trauma (CATS), depression and anxiety (RHS + PHQ-9), behavioural problems (SDQ), and self-harm and suicidality (SITBI) [97]. Investigators found that regardless of how they rated their own reading and writing abilities, or how many years of formal schooling they had, they were able to complete the computer-based assessments independently, and there was a minimal need for interpreters [67].

Several studies included pediatric populations in addition to adults, but these studies were not exclusive to children or adolescents [60,65,66,70,92,107]. These studies represent community and primary care settings that do not separate out the children, adolescents, women, and men, but rather provide services to families and any individual patient.

## 16. Mental Health Screening Tool Validation Studies

A total of 25 studies evaluated the validity of mental health screening tools among a cumulative sample of N = 4341 refugees and asylum seekers [42,44,46,48,50,52,55,64,65,66,67,70,72,80,82,86,87,89,95,99,100,101,105,106]. All of the included studies followed a cross-sectional study design. Screening took place post-arrival or in transit to the host country, which varied between studies and included the United States (n = 6), Sweden (n = 4), Germany (n = 5), Italy (n = 2), The Netherlands (n = 3), Australia (n = 1), Norway (n = 2), Greece (n = 1), and Switzerland (n = 1). Sixteen studies screened for mental health conditions among refugees, seven among asylum seekers, and one among unaccompanied migrant minors regardless of their legal migration status.

Screening targeted refugees and asylum seekers regardless of their age in nineteen studies [42,44,46,48,50,55,64,65,66,70,72,80,86,87,95,99,101,105,106], whereas it targeted adolescents and children (also referred to as minors) in five [52,67,82,89,100]. Studies seldom screened for just one mental health condition and most commonly screened for multiple; trauma-spectrum disorders, such as posttraumatic stress disorder (PTSD) and complex posttraumatic stress disorder (CPTSD), as well as traumatic events and experiences, were the most commonly screened conditions across studies (n = 21/25), followed by major depression (n = 12/25), anxiety (n = 8/25), somatization (n = 1/25), general psychological needs (n = 1/25), and environment safety (n = 1/25). Four screening tools emerged as the most commonly used among the identified validation studies:

The Harvard Trauma Questionnaire (HTQ) screened for posttraumatic stress disorder and traumatic events and was validated in six studies [42,67,72,87,95,101]. Translation of the tool to non-English languages was reported in four studies and the use of interpreters to facilitate its administration was reported in three. Sondergaard and colleagues discussed the superiority of the HTQ in screening PTSD compared to other screening tools [95]. Another study reported its higher sensitivity but warned of lower specificity [67]. Further, two studies reported the high validity of HTQ but discussed how certain items carry some threat to its validity when adapted across cultures [72,87]. Finally, Arnetz and colleagues discussed the importance of distinguishing two trauma subtypes when screening for PTSD using the HTQ: physical trauma and lack of necessities [42].

The Refugee Health Screener (RHS), both the 13- and 15-item versions, screened for depression, anxiety, and posttraumatic stress disorder and was validated in five studies [46,65,66,70,106]. The tool was translated in all studies and the use of interpreters to facilitate its administration was reported in four of five. All studies reported the adequate validity of the RHS tool in screening depression, anxiety, and posttraumatic stress disorder [46,65,66,70,106].

The Hopkins Symptom Checklist (HSCL-25) screened for depression and anxiety and was validated in two studies that translated the tool to the language of screened refugees and asylum seekers, and used interpretation services to facilitate its administration [67,72]. Jakobsen and colleagues reported the higher sensitivity but lower specificity of the tool when using a cut-off value of 2 [67]. Similarly, Kleijn and colleagues reported the high validity of the tool in screening depression and anxiety, but commented on the different meanings some items may carry across cultures [72].

The Mini International Neuropsychiatric Interview (MINI) was adapted and validated in two studies. The first study translated the instrument into Arabic and tested its validity in screening major depressive episodes, PTSD, panic disorders, generalized anxiety disorder, and agoraphobia among Syrian, Iraqi, and Palestinian refugees [50]. When compared to the PHQ-9, authors reported the high validity of the MINI instrument in screening for depression, anxiety, and PTSD [50]. The second study validated the major depression and PTSD sections of the French version of the MINI among asylum seekers from Europe, Asia, and Africa [55]. The authors of this study concluded that the tool could be used to systematically screen for depression and PTSD among refugees from different origins [55].

Other screening tools were sporadically tested for validity among refugees and asylum seekers and are described in Table 3 [44,48,64,80,82,89,99,105].

## 17. Discussion

Early screening and care programs for common mental health disorders in refugees and asylum seekers are emerging in many resettlement countries. Our scoping review aimed to characterize these approaches to inform a country-level resettlement policy and practice. We reported on two evaluations of pre-departure screening programs [4,39], 43 post-arrival screening programs, and 25 validation studies of screening tools/instruments. Our results characterized mental health assessment approaches, described approaches for special populations, such as women and children, and highlighted which screening tools are available, which have been validated among refugee populations, and where and how they have been used. Further, we summarized lessons learned and implementation considerations (see Box 2). Our results offered an overview of the international literature in this rapidly expanding area of refugee mental health research [108] and highlighted ongoing challenges and areas of uncertainty.

Box 2. **Lessons learned and implementation considerations**

**Who administers mental health screening?**
  Most mental health assessments are administered by a trained health professional with various levels of mental health expertise. This includes general practitioners, nurses, psychiatrists, psychologists, and community health workers. However, some tools can be self-administered (for example, the Refugee Health Screener) and completed on paper or using digital technology such as a tablet or computer. We identified a few mental health screening tools (PROTECT Questionnaire; STAR-MH; Refugee Health Screener) administered by staff without medical or psychological health training. Regardless of who administers the mental health assessment, numerous studies highlighted the importance of a trained interpreter or translator to assist in the assessment and prevent misinterpretations and miscommunications. Authors suggest the presence of trained interpreters improved the quality of communication and also served as cultural mediators [56].
**Which mental health screening tool should be administered?**
  There is no international consensus regarding the most effective mental health screening tool to be applied in the context of resettlement. While several tools are gaining popularity (for example, the Harvard Trauma Questionnaire or the Refugee Health Screener), there is currently insufficient effective research to guide the selection of mental health screening tools for national level programs. Currently, tools are chosen to reflect the cultural sensitivity and geographical diversity of refugee groups, but as migration patterns change rapidly, it is difficult to specify a singular set of tools that can be applicable to a large array of refugee populations [53]. It is well recognized that Western diagnostic classifications of mental health conditions have significant limitations with refugee populations because of variations in causality, sociocultural context, and symptom manifestation [6,66]. Authors agree that there is a need for culturally appropriate validated tools to detect mental health problems in refugees [48]. According to Poole et al. screening tools should be (1) self-reported or administered by trained non-medical health workers; (2) responsive to change; with (3) a demonstrated acceptable response rate, reliability, and validity in displaced populations; and (4) a minimal response burden [86].
**When should mental health screening take place?**
  Despite the existence of country-level guidance for pre-migration mental health screening (for example, from the USA [109], Australia [61], or New Zealand [110]), there are very few published reports evaluating these processes. The published literature shows that most assessments occur post-arrival to the resettlement state. Post-arrival programs can leverage community partnerships (e.g., [69]) and medical home models (e.g., [45]) to ensure efficient and appropriate linkages to care. Some studies noted the difficulty of following up with refugees as they often get transferred from one location to another in the first few months post-arrival [89,94]. Further, one Australian study reported challenges with the information transfer between and within pre-migration and post-arrival health systems, causing duplication of avoidable tests, increased costs, inefficiencies, and possible clinical consequences [61]. Evidence from the UK also identified critical operational issues with the information flow and supports the notion that further evaluation of pre-departure screening is warranted prior to widespread implementation [39]. To date, there is neither consensus nor sufficient program research to identify the optimal time to screen and assess the mental health needs of refugees and asylum seekers.
**Where does mental health screening take place?**
  The majority of mental health screening takes place in a primary care community setting, including refugee specific clinics or services where professionals were trained and familiar with the caseload. Buchwald et al. proposed that individuals presenting to primary care have come for help and accepted the “patient” role; therefore, psychiatric case finding and offering treatment may be less intrusive than it would be in other settings [49]. Furthermore, because this setting is not defined as “psychiatric,” the stigma associated with mental health treatment may be more easily minimized [6,49]. One study reported a high rate of refusal during a clinic-based post-arrival health assessment and found that mental health screening was more effective when conducted during a home visit [40].
**Do screening programs facilitate linkages to care?**
  Post-arrival screening programs usually include a linkage to care, either on-site or through referrals to community organizations or further specialized care. Programs which operate a medical home model can offer direct multidisciplinary care with allied health professionals and interpreters [45]. The evidence on pre-departure screening is less conclusive: while this information could function as an “early warning” to help local authorities prepare for individuals needing additional support, the impact of the screening is likely to be limited by resource availability and access to specialist mental health services [39]. Existing community resources may not be appropriate for the specific mental health needs of refugees who have fled conflict or experienced violence, torture, or trauma. However, as these pre-departure reports provide valuable information which is usually not available on arrival or takes time and trust to elicit from a patient, pre-departure mental health screening may help primary care providers save time and take appropriate action more proactively, thereby expediting the referral and provision of care [39].
**How can mental health screening be implemented?**
  Several studies highlighted that funding for mental health screening and care programs is essential [41,45,102]. Although many factors affect program success, the loss of program funds has been identified as the primary factor contributing to staff reductions and implementation failure [49]. Further, basic training about the context and important health issues of resettled refugees and administration procedures is necessary for all clinical and non-clinical staff [43]. Processes should be streamlined to reduce the time required to complete the assessment [39,43]. National training programs can provide technical assistance and support culturally relevant behaviours, attitudes, and policies in clinical practice [41,106], and help address mental health stigma [66]. Finally, the results from two studies suggest that sequential screening (i.e., categorizing refugees by level of risk to inform linkage to care) is a pragmatic strategy that can reduce the response burden and facilitate the detection of mental health conditions in settings with a scarcity of mental health specialists [80,86].

Among our identified studies, mental health screening programs were most common for adult refugee populations and most commonly delivered in primary care settings. We did identify studies on programs tailored to survivors of torture, women of reproductive age, and children and adolescents. We failed to identify studies on other vulnerable refugee subgroups, such as refugees who identify as LGBTQ+ and people living with disabilities. Cowen suggests that research on these vulnerable refugee populations is in its infancy [111]. For example, a 2019 report identified only six published studies on the mental health of sexual and gender minority refugees and asylum seekers [112]. These subgroups of refugees may be understudied due, in part, to complex intersecting identities and experiences which are not captured by immigration systems or other institutions. Concepts of “impairment”, “disability”, and “gender” can differ enormously among different cultures and societies, and these identities are often excluded from refugee registration and assistance programs [113]. Despite this, our findings noted that refugee mental health screening programs were often tailored to the refugee population by applying the principles of trauma-informed and person-centered care [114,115], including linguistically and culturally appropriate approaches and the evolution of gender- and age-specific programs.

Four studies focused on asylum seekers with an interest to identify and care for survivors of torture or violence [45,78,80,105]. Early health assessments and follow-ups for survivors of torture and violence are considered important to ensure the safety of these people [9]. Among survivors of torture, unmet mental health care needs are pervasive [116] and they are more likely to have PTSD and major depressive disorder in response to the trauma they had experienced [117]. Advocacy organizations, such as the Canadian Centre of Victims of Torture, can contribute to the resettlement of these populations by organizing networks of psychiatrists and refugee health practitioners, supporting mental health training, and providing medical-legal resources and general advocacy.

An overwhelming majority of studies (90%) reported on post-arrival mental health assessments. We only identified two reports on pre-departure mental health screening [4,39]. Pre-departure health assessments are an important established approach for individual and public health promotion, disease prevention, and facilitation of refugee integration in the resettlement country [19]. Due to the limited availability of and access to mental health services for refugees, countries such as the UK have identified a need for more pre-departure mental health screening to enable effective planning for resettlement [118]. However, the inclusion of mental health assessments within these pre-departure assessments is contentious given the lack of acceptability among refugee populations, lack of immediate and culturally appropriate interventions, and the challenges in information flow, suggesting that pre-departure mental health assessments are not ready to replace assessments on arrival [39,118,119].

Mental health screening was primarily administered by health professionals such as primary care providers (i.e., nurses, physicians) and mental health specialists (e.g., psychologists, psychiatrists). In some cases, a community health worker or research professional (often of the same cultural or linguistic group as the refugees themselves) conducted the assessments. We identified several studies where the assessment was made by a lay or administrative person [46,64,80,105]. Several studies also supported self-assessments, and demonstrated the potential value of digital approaches (e.g., laptops, tablets) when literacy levels allow [46,47,67,80,81,97]. Recent advances in automating screening with technologies such as mobile phones or tablets may facilitate the use of sequential screening in such settings [80,86]. Evidence that instrument performance is similar, regardless of the mode of administration (e.g., patient self-report, interviewer-administered either in-person or electronically) for self-reported depression supports the adoption of adaptive screening processes [81,86]. Mobile applications could offer youth, who otherwise lack independence, to access an assessment and information on their own [97,120]. Multidisciplinary programs for refugee children and their families have also suggested the merits and risks of including family members in the screening process [54].

A screening tool is assessed for sensitivity and specificity, but these are not constant or absolute performance measures. The performance of a tool will depend on the prevalence of the disorder within a population. The performance could also vary based on other characteristics of populations such as age, language, and culture. For this reason, a tool is often taken through a cross-cultural validation process to determine if it provides accurate and consistent measures across cultures. These tool characteristics are only the first part in the pathway to determining actual screening and care effectiveness (see Figure 1). A systematic review would be necessary to provide meaningful commentary on the effectiveness of tools along this care cascade (for e.g., [121]).

When selecting the most appropriate mental health screening tool, program developers must consider the specific refugee population, the estimated prevalence of mental health disorders, cultural idioms of distress, and the complex environmental stressors and traumatic events that may provoke mental health issues [21]. A comprehensive biopsychosocial assessment and meaningful intervention may need to occur over time with trusting, supportive therapeutic relationships and sometimes with specialized mental health care teams [21]. Literacy levels also play a role in determining the use of digital, self-administered, lay, or primary care provider assessment tools. Finally, women of a reproductive age often encounter their own unique challenges, and assessments should also factor in refugee lived experiences of pregnancy, childbirth, and raising children [21]. A recent review of mobile applications for women during pregnancy showed that technology-assisted approaches may improve timely access to mental health support and facilitate successful mental health care across different ethnicities [122]. Assessment tools for children may also need to include a broader spectrum of conditions and assess social determinants of health, developmental delays, family separation, and trauma [123].

## 18. Implications for Policy

The integration of refugees into society has significant health equity implications [124]. Policymakers need to ensure that new programs and policies are beneficial and not harmful for refugees prior to their implementation. While the benefit of the treatment of symptomatic mental illness among refugees is well-recognized [6], several factors influence the timing and feasibility of these assessments and subsequent treatment interventions. Ensuring refugee communities understand the goal and privacy of mental health screening, and ensuring access to care after screening, are essential factors for program success. Community-based screening with links to a holistic health settlement process is the most common and feasible approach. This may include formal routes of intersectoral collaboration between various services to provide multidisciplinary health care (e.g., medical, sexual, and reproductive health, mental health, allied health, educational agencies, social services, governmental bodies) [62]. Pre-departure overseas screening may provide some benefits, but more evaluation and refugee community support is required. Immigration policy should also be aware of mental health stigma and racism in the general population. Values of pluralism, equity, diversity, and inclusion within the receiving country’s society may also play a role in the mental health of refugees [13].

## 19. Implications for Practice

Most refugee mental health assessments were held in refugee-specific clinics or services with interdisciplinary primary care, primary care clinics, and hospital services. As cultural idioms of distress and the presentation of mental health symptoms vary across cultures, it is essential that health care workers are supported and equipped with the training and tools to adequately assess the mental health of refugees and asylum seekers in a sensitive and culturally appropriate manner [6,13,21]. Mental health care is often specialized, but most refugees and asylum seekers will initially present to primary care clinics [21]. It is important to remember that mental health disorders are most often experienced as social, cultural, spiritual, and medical issues, and these can lead to a range of first presentations, often to family, friends, and religious leaders. Primary care clinics need interdisciplinary programs with co-located physical and mental health services [90], and these programs need screening and monitoring tools to help engage team members in identifying illness, monitoring care, and detecting the severity of symptoms. In addition to primary care support, there will also be a need for more specialized clinicians and experts in cultural psychiatry who can meet the serious or severe mental health needs of patients.

## 20. Implications for Research

Our review highlights an array of programs and screening and diagnostic assessment tools in various languages across several ethnic groups of varied ages and experiences. Nonetheless, there still remains a gap in understanding which tools may be the most useful in each context, including increasing screening capacity, addressing acceptability concerns, and building trust in team-based interdisciplinary care. It also remains unknown what type of services and supports must be in place in order to safely and effectively implement pre-departure screening programs. Future realist-informed research may reveal contextual factors that influence program success, such as community outreach programs, rapid screening tools, community leaders, and primary care clinics [29,125]. Additionally, there were only two reports that assessed pre-departure mental health assessments [4,39]. It is important to understand if there are evidence-based benefits to performing the assessment of mental health at different time points (i.e., pre-departure, during their transition, or post-arrival) in order to determine how the timing of the assessment can impact immigration, referral to care, access to support, and overall health outcomes. Further, we identified few studies conducted among asylum seekers in detention, and this population warrants further research. Future research could include how information from these screening tools serves to empower screening and care programs, as well as to support physicians in diagnosis, care, and monitoring of patients. Further, evaluations should consider the impact of mental health screening on long-term resettlement outcomes.

## 21. Strengths and Limitations

This comprehensive review captures a large number of studies on refugee mental health screening tools and programs. We searched multiple databases, sought grey literature, and followed rigorous scoping review methods as suggested by the Joanna Briggs Institute according to a published protocol [34]. As a scoping review, our methods were not geared to synthesize the benefits and harms of screening programs but instead focused on characterizing existing mental health screening approaches. Within our results, there are examples of innovative community programs, a rich array of validated tools for screening and monitoring, and years of primary care screening experience. With additional research, the tools could guide the development of frameworks for mobile applications to improve access and allow anonymous use.

We, nonetheless, recognize several limitations of this work. Our focus was the screening components of programs and not cultural psychiatric consultation, psychotherapy services, and cultural navigation. Additionally, we excluded qualitative publications that focused on patient experiences rather than characteristics of early screening approaches. We restricted the ”resettlement” period to 12 months post-arrival. We recognize that many asylum seekers may not have received a decision or official refugee status within this time (i.e., they may spend several years as ”asylum seekers” as in Australia and Europe). We focused only on refugees and asylum seekers during resettlement, and excluded studies among general immigrant populations, refugee camp populations, and internally displaced populations, as these groups may each have unique levels of needs and complex pathways to mental health care.

Displacement and resettlement are often experienced at a collective level [6,54], and mental health includes a dynamic interplay of family and community. However, screening tends to occur at the individual level. Understanding the relationship between the patient, family, community, and provider is an important concept to consider. This level of complexity was outside the scope of this review but has significant implications for designing screening programs.

## 22. Deviations from Protocol

Due to time constraints, our grey literature was limited to searching government websites and reaching out to migration health experts in the field. We did not complete a grey literature search using Google search engines for NGOs and IGOs. We did not conduct a grey literature search focused on Europe. In the protocol we had stated: “We will search OpenGrey for grey literature originating from Europe. We will also use a Google Custom Search Engine to search the websites of over 1500 non-governmental organizations (NGOs) and over 400 international governmental organizations (IGOs)” [34].

## 23. Conclusions

Many refugees and asylum seekers face protracted migration status uncertainty, isolation, trauma, and additional delays in resettlement. Our review suggests early refugee mental health screening and care are feasible and often linked to established post-arrival medical screening programs in primary care. Vulnerable population programs for women, children, and survivors of torture are also emerging. More programmatic and realist evaluation research is needed to help programs select the most appropriate tools and processes for mental health screening and care programs in their context. Pre-departure screening exists but needs more evaluation.

## Figures and Tables

**Table 1:** Eligibility criteria.

SPIDER	Inclusion Criteria	Exclusion Criteria
Sample	Refugees and asylum seekers of all ages	All populations other than refugees and asylum seekers
Phenomenon of Interest	Pre-settlement overseas screening approaches or post-arrival (<12 months) approaches for mental health	Screening for other health conditions Routine screening after 1 year post-arrival
Design	Experimental and quasi-experimental studies Observational studies Program evaluations Resettlement handbooks and manuals Policy documents Development & validation studies Clinical assessment studies	Systematic reviews Scoping reviews Literature reviews Commentaries/opinion Theoretical papers
Evaluation	Characteristics of screening approaches	Estimates of effect Experiences/views
Research type	Quantitative, qualitative, or mixed-method documents published in peer-reviewed or grey literature	N/A
**Other**		
Year of publication	1995–2020	Prior to 1995
Language of publication	All languages eligible	N/A

**Table 2:** Characteristics of included studies (*n* = 70).

Study	Design	Setting	Timing of Assessment	Population	Mental Health Condition Assessed and Assessment Tool	Administration Details
Al-Obaidi (2015) [41]	Screening program	Refugee health care programs USA	Post-arrival	Refugees Burma, Haiti, Sudan, Iraq, Afghanistan, Somalia, and Cuba	Mental health assessment was not widely practiced (6/16 refugee-serving organizations). Assessment conducted by asking a few basic questions, e.g., for adults, any history of sleep problems, loss of energy, loss of appetite, feeling depressed, torture; and asking about the story of the refugee’s journey; for children, any history of seizures, learning problems, and head injuries In Denver, screening includes a DSM-based, non-validated questionnaire designed to detect major depression and PTSD The Seattle and New Mexico programs offer the Refugee Health Screener-15 (RHS-15) [PTSD, Depression, Anxiety]	In Denver, the mental health screening is conducted by a master’s level social worker Screening using the RHS-15 administered by health care providers. Clients themselves can complete the form if they have the appropriate level of education Interpreter not present Oral interview Assessment time not reported
Arnetz (2014) [42]	Validation study	Community centees USA	Post-arrival	Refugees Age 18–69 years, M = 33.41, SD = 11.29 54% male Iraq	Pre-immigration trauma exposure: Trauma section of Harvard Trauma Questionnaire (HTQ) PTSD: Civilian version of the PTSD Checklist (PCL-C) Depression: Hospital Anxiety and Depression Scale (HADS)	Administered by trained Arabic-speaking research personnel Interpreter present Self-assessment survey 120 min
Baird (2020) [43]	Screening program	Nurse-managed urban primary care safety-net clinic USA	Post-arrival	Refugees Mean age 32.8 years, SD = 13.6, Range = 17–72; 46% male. Country of origin not reported	Emotional distress including anxiety, depression, and PTSD: Refugee Health Screener-15 (RHS-15)	Administered by trained Doctor of Nursing Practice (DNP) family nurse practitioners Interpreter present Likert Scale, oral interview 60 min
Barbieri (2019) [44]	Validation study	Outpatient clinic and reception centre Italy	Post-arrival	Refugees and asylum seekers Age 18 and older (M = 25.1 years, SD = 6.7); 86% male Participants were from 19 African countries, mainly West Africa	PTSD: Posttraumatic Stress Disorder Checklist (PCL-5) CPTSD: International Trauma Questionnaire (ICD-11)	Administered by cultural mediator, a medical doctor, and a clinical psychologist Interpreter present Oral interview 60–90 min
Barnes (2001) [40]	Screening program	Local health department not affiliated with a hospital, combined with home visits USA	Post-arrival	Refugees Age 18–74 46.4% male Vietnam, Cuba, Bosnia, and African countries	Depression: DSM-IV criteria psychiatric interview	Administered by psychiatric residents with multi-national immigrant backgrounds (Africa and India) Interpretation provided by a paid interpreter (Vietnamese), administrative assistant (Bosnian), or nurse (Spanish). Family members also acted as interpreters (e.g., Farsi) Oral interview Assessment time not reported
Bertelsen (2018) [45]	Screening program	Primary care torture treatment centre USA	Post-arrival	Asylum seekers Mean age of 36.6 years (SD10.2) 66.2% male Majority from Sub-Saharan Africa (Guinea, Burkina Faso, and Democratic Republic of Congo being the most represented). Minority from Asia (with the Nepal and Tibet accounting for the majority of these)	PTSD: Harvard Trauma Questionnaire (HTQ) Major depressive disorder: Patient Health Questionnaire-9 (PHQ-9)	Administered by mental health professionals Interpreter present Oral interview Assessment time not reported
Bjarta (2018) [46]	Validation study	Asylum accommodations and health and service centres Sweden	Post-arrival	Refugees and asylum seekers aged 18 years and older; 72% male Afghanistan, Syria, Iraq, Iran, Eritrea, and Somalia	PTSD, depression, anxiety: Refugee Health Screener (RHS-13) Depression: Patient Health Questionnaire 9 (PHQ-9) Generalized anxiety: Generalized Anxiety Disorder 7 (GAD-7) PTSD: Primary Care PTSD-4 (PC-PTSD-4) Quality of life: World Health Organization Quality of Life–Brief Version (WHOQOL-BREF)	Self-assessment facilitated by bilingual administration staff Interpreter (bilingual staff) present Tablet. Audio support was available in Arabic, Dari, Farsi, and Tigrinya for individuals with low reading proficiency Assessment time not reported
Boyle (2019) [47]	Screening program	Refugee antenatal clinic Australia	Post-arrival	Refugees of childbearing age (below 35 years old) 100% female Afghanistan, Myanmar, Iraq, the Republic of South Sudan, and Sri Lanka.	Depression and anxiety: Edinburgh Postnatal Depression Scale (EPDS) Perinatal mental health: Monash Psychosocial Screening Tool	Self-assessment. Clinic staff available for assistance Interpreter present if needed Electronic via tablet 6–10 min
Brink (2016) [48]	Validation study	Primary care clinic USA	Post-arrival	Karen Refugees Aged 18–80 (M = 38.09, SD 13.82) 30% male Burma	PTSD and MDD: PTSD and MDD portions of the structured clinical interview for DSM disorders (SCID-CV for DSM-IV)	A physician with mental health training and a Karen interpreter administered the measures Oral interview and Likert-scale questionnaire Assessment time not reported
Buchwald (1995) [49]	Screening program	Ten refugee public health clinics USA	Post-arrival	Refugees Aged 16–85 yo (average age 31) 95% male Vietnam	Depression: Vietnamese Depression Scale	Administered by trained community health nurse Interpreter present Self-assessment 5 min
Churbaji (2020) [50]	Validation study	University hospital Germany	Post-arrival	Refugees Mean age 33.5 yo 75% male Syria, Iraq, Palestine	Depression and PTSD: Mini International Neuropsychiatric Interview (MINI) Depression: Patient Health Questionnaire, 9 (PHQ-9) PTSD: Harvard Trauma Questionnaire (HTQ)	Not reported
Cook (2015) [51]	Screening program	Primary care clinic USA	Post-arrival	Arabic-speaking Karen refugees Aged 18 yo and over (mean age: 35 (SD 14.6) 51% male	Four semi-structured items which asked retrospectively about lifetime experiences of primary and secondary war trauma and torture	Administered by trained research assistants (social work trainees) Interpreter present Oral interview Assessment time not reported
Di Pietro (2021) [52]	Validation study	Second-line reception centre Italy	Post-arrival	Unaccompanied migrant minors Age 12–18 yo 100% male Bangladesh, Egypt, Gambia, Senegal, Benin, Tunisia, Guinea Bissau, Morocco	Overall psychological needs: Unaccompanied Migrant Minors Questionnaire (AEGIS-Q)	Interpreter not present Self-administered with cultural mediator 20 min
Durieux-Paillard (2006) [53]	Validation study	Migrant health centre (University Hospital) Switzerland	Post-arrival	Asylum seekers Age 16 years or older	MDD and PTSD: Mini International Neuropsychiatric Interview (MINI)	Nurses without mental health training Interpreter present Oral interview 45 min
El Ghaziri (2019) [54]	Screening program	Centre for primary care and public health Switzerland	Post-arrival	Refugee families Members over age 8 yo 40–60% female Syria	Risk behaviours: ASSIST Support: Multidimensional Scale of Perceived Social Support (MSPSS) Parent-child relationship: Family Peer Relationship Questionnaire, Arabic version (A-FPRQ) Adults: major depressive disorder, panic disorder, posttraumatic stress disorder, generalized anxiety: Mini International Neuropsychiatric Interview (MINI) Children: major depressive disorder, panic disorder, separation anxiety, posttraumatic stress disorder: Mini International Neuropsychiatric Interview for Kids (MINI Kid)	Research assistant Interpreter present (research assistant) Administration mode not reported Assessment time not reported
Eytan (2007) [55]	Validation study	Primary care clinic Switzerland	In transition	Refugees Mean age 30 yo 75% male 33 countries, mostly Africa and Central or Eastern Europe	Major depressive episodes and PTSD: Mini International Neuropsychiatric Interview (MINI)	Administered by trained nurse Interpreter present Oral interview Assessment time not reported
Eytan (2002) [56]	Screening program	IME assessment Switzerland	In transition	Refugees, median age 24 yo 72% male Kosovo	MDD and PTSD: Mini International Neuropsychiatric Interview (MINI)	Administered by trained nurse Interpreter present Oral interview Assessment time not reported
Geltman (2005) [57]	Screening program	Unaccompanied Refugee Minors Program (URMP) sites USA	Post-arrival	Refugee minors, mean age 17.6 yo 84% male Sudan	PTSD: Harvard Trauma Questionnaire (HTQ) and Child Health Questionnaire (CHQ)	Administered by staff Interpreter not present Oral interview and self-assessment Assessment time not reported
Green (2021) [58]	Screening program	Primary care “office” USA	Post-arrival	Refugees, age 4–18 yo 56% male Bhutan, Burma, Democratic Republic of Congo/Burundi, Iraq, Somalia	PTSD, depression, trauma: Strengths and Difficulties Questionnaire (SDQ)	Administered by interpreters Interpreter present Oral interview Assessment time not reported
Hanes (2017) [59]	Screening program	Hospital refugee health service Australia	Post-arrival	Refugees Age 2–16 yo mean age 9.4 yo 49% male Top 7 countries: Burma, Afghanistan, Sudan, Ethiopia Congo, Somalia, Iran	Adverse childhood experiences: Strengths and Difficulties Questionnaire (SDQ)	Interpreter present Self-administered Assessment time not reported
Hauff (1995) [60]	Screening program	n/a Norway	Post-arrival	Refugees Age over 15 yo 79% male Vietnam	Psychiatric disorders: Symptom Checklist 90 (SCL-90) and Present State Examination (PSE)	Researcher Interpreter present Oral interview Assessment time not reported
Heenan (2019) [61]	Screening program	Specialist immigrant health service within a children’s hospital Australia	Post-arrival	Refugee children Age 7 months to 16 years old 64.8% male Syria, Iraq	Mental health (including PTSD) and development screening was conducted, but no assessment tool is described	Refugee health program nurses Primary care health assessment Assessment time not reported
Hirani (2018) [62]	Screening program	Tertiary refugee health service Australia	Post-arrival	Adolescent refugees Age 12–17 years old (mean age 14; 49% male) 15 countries (Middle East, Africa, Asia)	Psychosocial assessment: Home, Education/Eating, Activities, Drugs, Sexuality, Suicide/mental health’ (HEADSS) Questionnaire	Interviewer Interpreter present Oral interview 25–60 min
Hobbs (2002) [63]	Screening program	Public health hospital New Zealand	Post-arrival	Asylum seekers Age 0–60+ years old 68.1% male Middle Eastern countries	Symptoms, or history of symptoms, of psychological illness: Auckland Public Health Protection Asylum Seekers Screening	Clinic staff Health screening Assessment time not reported
Hocking (2018) [64]	Validation study	Asylum seeker welfare centre Australia	Post-arrival	Refugees 19–82 yo, median age 33 69.8% male Mostly from countries in Africa and Asia	Major depressive disorder (MDD) and post-traumatic stress disorder (PTSD): Mental Health Screening Tool for Asylum seekers and Refugees (STAR-MH)	Administered by trained non-mental health workers Interpreter present Oral interview 6 min
Hollifield (2013) [65]	Validation study	Public health centre USA	Post-arrival	Refugees Age over 14 yo 50% male Bhutan, Burma, and Iraq	Anxiety, depression, PTSD: Refugee Health Screener-15 (RHS-15)	Administered by physicians or public health clinic staff Interpreter present Oral interview 4–12 min
Hollifield (2016) [66]	Validation study	Public health centre USA	Post-arrival	Refugees Age over 14 yo 50% male Bhutan, Burma, and Iraq	Anxiety and depression: Hopkins Symptom Checklist 25 (HSCL-25) PTSD: Posttraumatic Symptom Scale- Self Report (PSS-SR) Anxiety, depression, PTSD: Refugee Health Screener-15 (RHS-15)	Administered by trained public health nurses Interpreter not present Oral interview Assessment time not reported
Hough (2019) [39]	Screening program	Refugee clinic Lebanon	Pre-departure	Refugees 18 years and above 50% male Syria	General mental health: Global Mental Health Assessment Tool (GMHAT)	Administered by healthcare professionals (psychiatrist, general physician, pediatrician, and two nurses) Administrators served as translators Computerized tool 15–20 min
International Organization for Migration (IOM) (2020) [4]	Screening program	IOM migration health assessment clinics Lebanon, Turkey, and Jordan	Pre-departure	Refugees: majority younger than 30 (67.1%), with the highest number in the under-10 age group 51.2% male	Not described	Not described
Jakobsen (2017) [67]	Validation study	Setting not reported Norway	Post-arrival	Unaccompanied adolescent asylum seekers Age 15–18 years old (mean: 16.2) 100% male Afghanistan, Somalia	PTSD, anxiety, and depression: combined Hopkins Symptom Checklist-25 (HSCL-25) and Harvard Trauma Questionnaire (HTQ- IV)	Interpreter present Self-administered via laptop computer Assessment duration not reported
Javanbakht (2019) [68]	Screening program	Primary care clinic USA	Post-arrival	Refugees Age 18–65 yo 52.9% male Syria	PTSD: PTSD Checklist Civilian version (PCL-C) Anxiety and depression: Hopkins Symptom Checklist 25 items (HSCL-25)	Research assistant Interpreter present (research assistant) Self-assessment 20 min (5–10 min per tool)
Johnson-Agbakwu (2014) [69]	Screening program	Refugee women’s health clinic with a behavioural health partnership USA	Post-arrival	Refugees Age 18 years and older 100% female Iraq, Burma, Somalia	Anxiety, depression, PTSD: Refugee Health Screener-15 (RHS-15)	Cultural health navigator (served as interpreter) Oral interview 5–10 min
Kaltenbach (2017) [70]	Validation study	Refugee accommodation Germany	In transition	Refugees Age over 12 yo, median age 28.79 yo Majority from Syria, minority from Afghanistan, Albania, Kosovo, Serbia, Iraq, Macedonia, Somalia, Georgia	PTSD, depression, anxiety: Refugee Health Screener-15 (RHS-15) Semi-structured interview: PTSD: Post-traumatic Stress Disorder Checklist-5 (PCL-5) Depression: Refugee Health Screener (RHS-15)—only the first 13 questions Trauma exposure: Life Events Checklist (LEC-5) Psychological distress: semi-structured interview via Brief Symptom Inventory -18	RHS Self-administered Interpreter present if needed 10–30 min Semi-structured interview by a clinical psychologist Interpreter present Oral interview 90 min
Kennedy (1999) [71]	Screening program	Primary care clinic (University Hospital) USA	Post-arrival	Adult refugees and their children	Depression, anxiety, and PTSD: A set of questions about history of imprisonment, trauma, or torture + a 25-item, self-administered symptom checklist that surveys for symptoms of depression, anxiety, and PTSD. The checklist was developed by Dawn Noggle, PhD, of the International Rescue Committee in Arizona. In addition, parents are asked standard questions about their children’s adjustment and symptoms of stress or depression	Administered by nurse or physician Interpreter present Self-assessment symptom checklist Assessment time not reported
Kleijn (2001) [72]	Validation study	Psychiatric clinic Netherlands	Post-arrival	Refugees 81% male Arabic, Farsi, or Serbo-Croatian speaking regions	PTSD: The Harvard Trauma Questionnaire (HTQ) Depression and Anxiety: Hopkins Symptoms Checklist-25(HSCL-25)	Administered by psychologist or psychiatrist Interpreter present Self-assessment Assessment time not reported
Kleinert (2019) [73]	Screening program	Primary care centre within a reception centre Germany	Post-arrival	Refugee and asylum seekers median age of all patients was 26 years, SD 18.529 51% of asylum-seeker patients and 49% of resettlement-refugee patients were female Iraq, Syria, Afghanistan, Georgia, Iran	Mental and behavioural disorders classified by ICD 10: Digital Communication Assistance Tool (DCAT)	General practitioners and nurses Digital Communication Assistance Tool (DCAT) via tablet No interpreter present Self-assessment Assessment time not reported
Kroger (2016) [74]	Screening Program	Reception centre Germany	Post-arrival	Refugees and asylum seekers Average age 30.5 88.2% male Balkan States, Middle East, Northern Africa, rest of Africa	PTSD: Post-traumatic Diagnostic Scale-8 (PDS-8) Depression: Patient Health Questionnaire (PHQ-8)	Administered by psychological psychotherapist, medical assistant, or psychology undergraduate students Interpreter present Oral interview 15–90 min
LeMaster (2018) [75]	Screening program	Local resettlement agencies USA	Post-arrival	Refugees Mean age 33.4 Iraq	PTSD: civilian version of the PTSD Checklist (PCL-C) and Harvard Trauma Questionnaire (HTQ) Depression: Hospital Anxiety and Depression Scale	Administered by a trained Arabic-speaking interviewer Interpreter present Oral interview 120 min
Lillee (2015) [76]	Screening program	Humanitarian entrant health service Australia	Post-arrival	Refugees Age 18–70 48.7% male Africa, South-Eastern and South-Western Asia	Non-specific psychological distress: The Kessler Psychological Distress Scale (K10) PTSD: PTSD treatment screener	Administered by physicians Interpreter present Oral interview or self-assessment 10–15 min
Loutan (1999) [77]	Screening program	University Hospital Switzerland	Post-arrival	Refugees Median age 27 67% male Yugoslavia, Somalia, Angola, Sri-Lanka	Physical and psychological symptoms and previous exposure to traumatic events: No name; short questionnaire developed and tested at the Policlinic	Administered by trained nurses (who were multilingual) Interpreter not present Oral interview 15 min
Masmas (2008) [78]	Screening program	Reception centre Denmark	Post-arrival	Asylum seekers, average age 32 years (16–73 years) 71% male Afghanistan, Iraq, Iran, Syria, and Chechnya	PTSD: International Classification of Disease Codes (ICD-10) Overall psychological health: WHO’s General Health Questionnaire	Administered by trained health care professionals Translator available if needed Oral interview 60 min
McLeod (2005) [79]	Screening program	Refugee resettlement centre medical clinic New Zealand	Post-arrival	Refugees: majority 20–34 years old 53.2% male, 34 different nationalities, majority Iraqi, Somali, Ethiopian	Psychosocial assessment—screening tool not reported	Administered by trained health care professionals Administration details not reported
Mewes 2018 [80]	Validation study	Asylum accommodation or at meeting points for asylum seekers Germany	Post-arrival	Asylum seekers Aged 18 years and older (M = 31.9 years SD 7.8), 67% male Most participants came from Iran, Afghanistan, Syria, or African countries	PTSD and depression: Process of Recognition and Orientation of Torture Victims in European Countries to Facilitate Care and Treatment (PROTECT) Questionnaire PTSD: Posttraumatic Diagnostic Scale (PDS) Depression: Patient Health Questionnaire-9 (PHQ9)	Self-assessment The software ‘MultiCasi’ was used via a laptop with touchscreen Interpreter present Assessment time not reported
Morina (2017) [81]	Screening program	Clinical setting outpatient clinic Switzerland	Post-arrival	Refugees Aged 28–64 (mean 50.07, SD 8.65) 77% male Afghanistan, Sri Lanka, Iraq, Turkey, Sudan	PTSD: Posttraumatic Diagnostic Scale based on DSM-5 (PDS) Depression: Hopkins Symptom Checklist-25 (HSCL-25) Quality of Life: EUROHIS-QoL Questionnaire	Interview with therapist Interpreter present Oral interview Assessed in 24 min or Computer assisted self-interviews using multi-adaptive psychological screening software (MAPSS) Tablet Assessed in 9 min
Nehring (2021) [82]	Validation study	Reception camp Germany	Post-arrival	Refugee children Age 4–14 years (mean: 8.9 years (SD: 2.8)) tijana59.0% male Syria	PTSD: Child Behaviour Checklist (CBCL)	Child and adolescent psychiatrists Interpreter and native speaking doctors were present Oral interview with parents and children The duration of all examinations lasted 1–2 days for one family
Nikendei (2019) [83]	Screening program	Outpatient clinic Germany	Post-arrival	Asylum seekers Age over 18 yo Asia, Africa, Eastern Europe	PTSD: Primary Care PTSD Screen for DSM-5 (PC-PTSD-5) Depression: Patient Health Questionnaire-2 (PHQ-2) General anxiety: Generalized Anxiety Disorder (GAD-2) Panic symptoms: PHQ-PD Social well-being: World Health Organization- Five Well-Being Index (WHO-5) Alcohol and drug addiction: three screening questions derived from the screening questions from the SCID (Structured Clinical Interview)	Research assistant Interpreter available Self-assessment Assessment time not reported
Ovitt (2003) [84]	Screening program	Resettlement office or at participants’ homes USA	Post-arrival	Refugees Ages 29–72 yo 50% male Bosnia	Anxiety and depression: The Hopkins Symptom Checklist-25 (HSCL-25) and a client questionnaire	Psychiatrist or medical doctor Interpreter not present Oral interview Assessment time not reported
Polcher (2016) [85]	Screening program	Community health centre USA	Post-arrival	Refugees Ages 18 yo or older tijana41% male Bhutan, Iraq, Somalia, Congo, Sudan, Burma, Iran, and Eritrea	Anxiety, depression and PTSD: Refugee Health Screener–15 (RHS-15)	Administered by trained interpreters and medical assistants Interpreter present Oral interview 10–15 min
Poole (2020) [86]	Validation study	Refugee camp Greece	In transition	Refugees Mean age 30 years, range 18–61. 59% male Syria	Major Depressive Disorder (MDD): Patient Health Questionnaires (PHQ-2 and PHQ-8)	Administered by research personnel Arabic-English interpreter present Oral interview Assessment time not reported
Rasmussen (2015) [87]	Validation study	Primary care clinic USA	Post-arrival	Asylum seekers Mean age 34.9 yo 59% male West Africa, Himalayan Asia, and Central Africa	PTSD: Harvard Trauma Questionnaire (HTQ)	Administered by trained interpreters Interpreter present Oral interview Assessment time not reported
Richter (2015) [88]	Screening program	Central reception facilities Germany	Post-arrival	Asylum seekers Mean age 31.9 years old, SD 10.6 66.8% male Iran, Russia, Afghanistan, and Iraq	General psychiatric assessment: Structured diagnostic interview MINI-International Neuropsychiatric Interview (MINI-Plus) Essen Trauma Inventory (ETI) Brief Symptom Inventory (BSI) Montgomery-Asberg Depression Scale (MADRS) WHO-5 Pittsburgh Sleep Quality Index (PSQI)	Administered by a physician Interpreter present Oral interview 3 h (two 1.5 h sessions)
Salari (2017) [89]	Validation study	Primary care clinic Sweden	Post-arrival	Refugees Ages 9–18 97.6% male Majority from Afghanistan. Others from Iran, Syria, Iraq, Pakistan, Somalia, Eritrea, Ethiopia, Libya, and Lebanon	PTSD: Children’s Revised Impact of Event Scale (CRIES-8)	Clinicians and nurses Interpreter present if needed Self-assessment Assessment time not reported
Savin (2005) [90]	Screening program	Primary care clinic USA	Post-arrival	Refugees Ages 18 years old and over (mean age 27.4) 51.5% male 24 countries of origin: most frequently Bosnia, Russia, Ukraine, Sudan, Somalia, Ethiopia, Afghanistan, Burma, Vietnam, Iran, and Iraq	PTSD, anxiety, depression: 25-item psychiatric symptom checklist derived from the Diagnostic and Statistical Manual of Mental Disorders, Fourth Edition (DSM-IV)	Administered by a team composed of a case manager from a publicly funded resettlement agency, a primary care nurse experienced with culturally diverse populations, a primary care physician, and if needed, a psychologist or psychiatrist. Nurses primarily administered the screening tool Interpreter present Oral interview Assessment time not reported
Schweitzer (2011) [91]	Screening program	Settlement service Australia	Post-arrival	Refugees Mean age 34.13 yo (range 18–80 yo) 43.9% male Burma	Pre-migration trauma: HTQ Depression, anxiety, somatization: Hopkins Symptom Checklist-37 (HSCL-37) Post-migration stressors: Post-migration Living Difficulties Checklist	Researchers and counsellors Interpreter present Oral interview 2–3 h
Seagle (2019) [92]	Screening program	Outpatient clinics USA	Post-arrival	Administrative sample, 64% refugees Age 14 years and older 42.6% male Cuba, Burma, Afghanistan, Bhutan, Iraq, Somalia, Iran, Ethiopia, Syria	Not reported; however, the authors state that clinicians may consider the use of the Refugee Health Screener-15 (RHS-15), Harvard Trauma Questionnaire (HTQ), Vietnamese Depression Scale (VDS), New Mexico Refugee Symptom Checklist 121 (NMRSCL-121), and the Hopkins Symptom Checklist 25 (HSCL-25) Georgia public health officials recommend use of the RHS-15	Administered by clinicians Interpreter present Self-assessment and oral interview Time of assessment was variable
Shannon (2015) [93]	Screening program	Primary care clinic USA	Post-arrival	Refugees Mean age 35.27 51.4% male Burma	PTSD, distress, somatic complaints, depression: unspecified 32-item questionnaire	Administered by trained research staff Interpreter present Self-assessment 45 min
Sondergaard (2001) [94]	Screening program	Reception centre Sweden	Post-arrival	Refugees Ages 18–48, mean age 35 yo 63% male Iraq	PTSD: Questionnaire developed uniquely for this study, based on the Holmes-Rahe Life Event Questionnaire	Assessor background not reported Interpreter not present Self-assessment Assessment duration not reported
Sondergaard (2003) [95]	Validation study	Reception centre Sweden	Post-arrival	Refugees Ages 18–48, mean age 35 yo 63% male Iraq	Mental health screen using Health Leaflet: Harvard Trauma Questionnaire (HTQ), Impact of Event Scale (IES-22), General Health Questionnaire (GHQ-28), Hopkins Symptoms Checklist (HSCL-25) PTSD: Structured Clinical Interview for DSM Disorders (SCID) or Clinician Administered PTSD Scale for DSM-5 (CAPS) Depression: Hopkins Symptoms Checklist (HSCL-25)	Administered by case manager Interpreter not present Self-assessment Assessment time not reported
Stingl (2019) [96]	Screening program	Reception centre (RC) & communal accommodation (CU) Germany	Post-arrival	Refugees Mean age 25.6 (RC), 28.9 (CU) 92.9% (RC), 69.8% (CU) male Afghanistan, Algeria, Ethiopia, Eritrea, Iraq, Iran, Somalia, Syria	Depression, anxiety, and PTSD: Refugee Health Screener (RHS-15)	Administered by doctorate students and a linguist Interpreter present Written Likert-scale 4–12 min
Sukale (2017) [97]	Screening program	Clearing and pre-clearing institution Germany	Post-arrival	Refugee minors Age 16.24 years, SD 1.03 100% male Syria, Afghanistan, Iran, Somalia, Sudan, Iraq	Providing Online Resource and Trauma Assessment (PORTA) screening tool, which comprises of disorder-specific questionnaires: Trauma: CATS Depression and Anxiety: Refugee Health Screener (RHS-15) + Patient Health Questionnaire-9 (PHQ-9) Behavioural problems: Strengths and Difficulties Questionnaire Self harm and suicidality: SITBI	Self-administered Interpreter present if needed Online questionnaire via computer (PORTA) 30–90 min
Tay (2013) [98]	Screening program	Reception centre Australia	In transition	Asylum seekers Mean age 39 65% male 18 countries- majority from Iran, Ghana, Zimbabwe, Afghanistan, and China	PTSD and depression: Structured Clinical Interview for DSM-IV (SCID) PTSD: Harvard Trauma Questionnaire (HTQ) Depression: Hopkins Symptoms Checklist (HSCL-25)	Assessment by psychologists Interpreter present Oral interview 120 min
Thulesius (1999) [99]	Validation study	Asylum centre (refugees) and Healthcare clinic (Swedish comparison group) Netherlands	Post-arrival	Refugees Mean age 33.7 58% male Bosnia-Herzegovina	PTSD and depression: Modified Posttraumatic Symptom Scale (PTSS-10-70)	Assessor background not reported Interpreter not present Self-assessment Assessment time not reported
van Os (2018) [100]	Screening program	National intermediary organizations Netherlands	Post-arrival	Refugees and asylum seekers—16 unaccompanied children (15–18 years) and 11 accompanied children (4–16 years) 63% male 44% from Afghanistan	Well-being and child development: Best Interests of the Child (BIC-Q), Strengths and Difficulties Questionnaire (SDQ) Stressful life events: Stressful Life Events (SLE) PTSD: Reactions of Adolescents on Traumatic Stress (RATS)	Assessment by trained professionals Interpreter present Self-assessment and oral interview 180–240 min
Van Dijk (1999) [101]	Validation study	Psychiatric hospital Netherlands	Post-arrival	Refugees Mean age 35.7 years, range 17–70 67% male Diverse nationalities (country not specified)	PTSD: Harvard Trauma Questionnaire (HTQ), Hopkins Symptom Check List-90 (HSCL-90), DSM-IV	Administered by psychiatrists and psychological assistants Interpreter present Oral interview Assessment time not reported
Vergara (2003) [102]	Screening program	Refugee health program USA	Post-arrival	26,374 refugees, or 38.1% of all refugees, resettling in the United States during fiscal year 1997 No additional characteristics reported	3/9 sites offered mental status examinations during the domestic refugee health assessment. Specific mental health conditions and assessment tools not reported	Not reported
Weine (1998) [103]	Screening program	Primary care clinic USA	Post-arrival	Refugees Ages 13–59 yo 50% male Bosnia	PTSD: PTSD Symptoms Scale, the Communal Traumatic Experiences Inventory, the Global Assessment of Functioning (DSM-IV), and the Symptoms Checklist 90 (SCL-90-R)	Administered by mental health professionals Interpreter present Oral interview 60–120 min
Willey (2020) [104]	Screening program	Refugee antenatal clinic Australia	Post-arrival	Women from a refugee background or considered refugee-like, i.e., arrived in Australia on a spousal visa from a refugee-source country such as Afghanistan	Depression and anxiety: Edinburgh Postnatal Depression Scale (EPDS) Perinatal mental health: Monash Psychosocial Screening Tool	Administered by maternal care staff (midwives, bi-cultural workers) Interpreter present Electronic tablet 10 min
Wulfes (2019) [105]	Validation study	Refugee accommodations Germany	Post-arrival	Refugee and asylum seekers Ages 17–90 yo, average age 32.9 64.4% male Syria, Iraq, Afghanistan, Iran, Sudan	1. Posttraumatic stress screening: PQ 2. Traumatic events: a list of events that was modified from those included in the Posttraumatic Diagnostic Scale (PDS) 3. Posttraumatic stress symptoms: PDS-8 4. Depression: Patient Health Questionnaire 9 (PHQ-9) 5. Axis I disorders: Structured Clinical Interview for DSM Disorders (SCID)	Administered by staff without medical or psychological health training I nterpreter not present Self-assessment Assessment time not reported
Yalim (2021) [106]	Screening program	Refugee resettlement agency/home visit USA	Post-arrival	Refugees Aged 18 years and older (mean age 36.38 years, SD 12.5) Majority from Democratic Republic of Congo, Iraq, Syria, and Eritrea	PTSD, depression, anxiety: Refugee Health Screener (RHS-15)	Administered by research personnel Interpreter present Oral interview or self-assessment, depending on the literacy level of the participant 20–30 min
Young (2016) [107]	Screening program	Detention centres Australia	Post-arrival	Asylum seekers and refugees (detainees) All ages 73% male	Depression, anxiety, and PTSD: Self-rated Kessler 10 (K-10) The Harvard Trauma Questionnaire (HTQ) The Clinician-rated Health of the Nation Outcome Scores (HoNOS) The Clinician-rated Health of the Nation Child and Adolescent Outcome Scores (HoNOSCA)	Administered by mental health professionals (nurse or psychologist) Interpreter present Self-assessment Assessment time not reported

**Table 3:** Mental Health Screening Tool Characteristics.

	Screening Tool	Studies	Mental Health Conditions Assessed	Administrator	Languages
1	Family Peer Relationship Questionnaire, Arabic version (A-FPRQ)	El Ghaziri 2019	OTHER	unspecified	unspecified
2	Unaccompanied Migrant Minors Questionnaire (AEGIS-Q)	Di Pietro 2020	OTHER	unspecified	unspecified
3	Al-Obaidi et al. DSM-based non-validated questionnaire	Al-Obaidi 2015	Depression, PTSD	Master’s level social worker	unspecified
4	Arab Acculturation Scale	LeMaster 2018	PTSD, Depression, OTHER	unspecified	unspecified
5	Alcohol, Smoking and Substance Involvement Screening Test (ASSIST)	El Ghaziri 2019	Substance Use Disorder	unspecified	unspecified
6	Best Interests of the Child (BIC-Q)	van Os 2018	Disruptive Behaviour Disorders, Depression	unspecified	Arabic, Dari, Farsi, Somali
7	Brief Symptom Inventory-18 (BSI-18)	Kaltenbach 2017 Richter 2015	Depression, PTSD, Somatoform Disorders	unspecified	Albanian, Arabic, Farsi, Kurdish, Russian, Serbian, Somali
8	Child Behaviour Checklist (CBCL)	Nehring 2021	PTSD	MHS	German
9	Child Health Questionnaire (CHQ)	Geltman 2005	OTHER	Community health worker (CHW)	English
10	Children’s Revised Impact of Event Scale (CRIES-8)	Salari 2017	PTSD	Primary care provider (PCP)	Arabic, Dari, Farsi, Kurdish/Sorani, Swedish
11	Communal Traumatic Experiences Inventory (CTEI)	Weine 1998	PTSD, Depression	Mental health specialist (MHS), PCP, lay person (LAY)	Croatian
12	Cook et al. author-developed interview	Cook 2015	OTHER	Research assistants trained Master’s or Ph.D. level social work students	unspecified
13	Digital Communication Assistance Tool (DCAT)	Kleinert 2019	PTSD, Depression, Anxiety, Substance Use Disorder, Disruptive Behaviour Disorders, Somatoform Disorders, OTHER	Self-assessment, PCP	Modern Standard Arabic, Arabic Syrian, Arabic Egyptian, Arabic Tunisian, Arabic Moroccan, Turkish, Persian, Kurdish Sorani, Kurdish Kurmanji, Kurdish Feyli, Pashto Kandahari, Pashto Mazurka
14	Edinburgh Postnatal Depression Scale (EPDS)	Boyle 2019 Willey 2020	Depression	MHS, PCP, CHW, LAY	unspecified
15	Essen Trauma Inventory (ETI)	Richter 2015	Trauma	MHS	German
16	Eytan et al. (2002) author-developed interview	Eytan 2002	Health Conditions, Presence of Symptoms and Previous Exposure to Trauma	Nurses	French, German, Italian, Spanish, Portuguese, English
17	Family Assessment Device (FAD)	El Ghaziri 2019	OTHER	unspecified	unspecified
18	Geltman et al. author-developed ad-hoc assessment	Geltman 2005	Emotionally Traumatic Exposures	Staff from local URMO agencies	English
19	Generalized Anxiety Disorder 7 (GAD-7)	Bjarta 2018	Anxiety	MHS, PCP, CHW	Arabic, Dari
20	GB	El Ghaziri 2019	OTHER	unspecified	unspecified
21	General Health Questionnaire (GHQ-28)	Sondergaard 2003	Depression	Self-rating	unspecified
22	Global Assessment of Functioning Scale (DSM-IV)	Weine 1998	OTHER	MHS, PCP, CHW	Croatian
23	Global Mental Health Assessment Tool (GMHAT)	Hough 2019	Anxiety, Depression, Substance Use Disorders, Disruptive Behaviour Disorders	PCP, MHS	English, Arabic
24	Hospital Anxiety and Depression Scale (HADS)	Arnetz 2014 LeMaster 2018	Anxiety, Depression	CHW	Arabic
25	Hassles Scale	LeMaster 2018	OTHER	CHW	unspecified
26	Home, Education/Eating, Activities, Drugs, Sexuality, Suicide/mental health (HEADSS) Questionnaire	Hirani 2016	OTHER	unspecified	English
27	Health Leaflet (HL)	Sondergaard 2003	PTSD	Self-assessment	unspecified
28	Kennedy et al. author-developed tool	Kennedy 1999	Depression, Anxiety, PTSD	Self-assessment	unspecified
29	Clinician-rated Health of the Nation Outcome Scores (HoNOS)	Young 2016	Depression, Substance Use Disorders, Disruptive Behaviour Disorders	MHS, PCP	unspecified
30	Clinician-rated Health of the Nation Child and Adolescent Outcome Scores (HoNOSCA)	Young 2016	Depression, Substance Use Disorders, Disruptive Behaviour Disorders	MHS, PCP	unspecified
31	Hopkins Symptom Checklist-25 (HSCL-25)	Jakobsen 2017 Javanbakht 2019 Kleijn 2001 Ovitt 2003 Schweitzer 2011 Sondergaard 2003 Tay 2013 Van Dijk 1999	Anxiety, Depression	MHS, PCP, CHW	Arabic, Farsi, Russian, Bosnian-Serbo-Croatian
32	Harvard Trauma Questionnaire (HTQ)	Arnetz 2014 Bertelsen 2018 Churbaji 2020 Geltman 2005 Jakobsen 2017 Kleijn 2001 LeMaster 2018 Rasmussen 2015 Schweitzer 2011 Sondergaard 2003 Tay 2013 Young 2016 Van Dijk 1999	PTSD	MHS, PCP, CHW	Arabic, Cambodian, Dutch, English, Farsi, French, Laotian, Russian, Serbo-Croatian, Vietnamese
33	Impact of Event Scale (IES-22)	Sondergaard 2003	Trauma	Self-rating	unspecified
34	Interpersonal Support Evaluation Checklist (ISEL)	LeMaster 2018	OTHER	CHW	unspecified
35	ICD-11 International Trauma Questionnaire (ITQ)	Barbieri 2019	PTSD	MHS, PCP, CHW	Arabic, English, French
36	Kaltenbach et al. author-developed questionnaire	Kaltenbach 2017	Daily Functioning	Clinical psychologists	unspecified
37	Karen Mental Health Screener	Brink 2016	PTSD, Depression	PCP	Karenic
38	Self-rated Kessler 10 (K-10)	Lillee 2015 Young 2016	OTHER	Self-administered (SA)	English, Kurdish, Pashto
39	Life Events Checklist (LEC-5)	Kaltenbach 2017	OTHER	unspecified	Arabic, Albanian, Farsi, Kurdish, Russian, Serbian, Somali
39	Loutan et al. author-developed questionnaire	Loutan 1999	Anxiety, Depression, PTSD, and Traumatic Events	Nurse	French, English, Italian, Spanish and German
40	Multi-Adaptive Psychological Screening Software (MAPSS)	Morina 2017	Depression, PTSD	MHS, PCP	Arabic, Farsi, Tamil, Turkish
41	Mini International Neuropsychiatric Interview (MINI)	Churbaji 2020 Durieux-Paillard 2006 El Ghaziri 2019 Eytan 2007 Kaltenbach 2017 Richter 2015	Depression, Anxiety, Substance Use Disorders, Disruptive Behaviour Disorders, PTSD, Somatoform Disorders	MHS, PCP	70+ languages
42	Mini International Neuropsychiatric Interview for Children and Adolescents (MINI-KID)	El Ghaziri 2019	Depression, Anxiety, Substance Use Disorders, Disruptive Behaviour Disorders, PTSD, Somatoform Disorders	MHS, PCP	70+ languages
43	Monash Health Psychosocial Needs Assessment (Psychosocial Screening Tool)	Boyle 2019 Willey 2020	OTHER	MHS, PCP, CHW, LAY, Self-administered	unspecified
44	Montgomery-Asberg Depression Scale (MADRS)	Richter 2015	Depression	MHS, PCP	unspecified
45	Multidimensional Scale of Perceived Social Support (MSPSS)	El Ghaziri 2019	OTHER	MHS, PCP, CHW	unspecified
46	Nikendei et al. author-developed interviews	Nikendei 2019	Substance Use	Research personnel	English, German, French, Persian, Arabic, Turkish, Kurmanji (Northern Kurdish), Urdu, Hausa, Russian, Serbian, Albanian, Macedonian, Georgian, Mandinka, Tigrinya
47	Ovitt et al. author-developed client questionnaire	Ovitt 2003	OTHER		
48	Primary Care PTSD-4 (PC-PTSD-4)	Bjarta 2018	PTSD	MHS, PCP, CHW, LAY, SA	Arabic, Dari
49	PC-PTSD-5	Nikendei 2019	PTSD	Trained research assistants	English, German, French, Persian, Arabic, Turkish, Kurmanji (Northern Kurdish), Urdu, Hausa, Russian, Serbian, Albanian, Macedonian, Georgian, Mandinka, Tigrinya
50	PTSD Checklist for DSM-5 (PCL-5)	Barbieri 2019 Kaltenbach 2017	PTSD	MHS, PCP	Arabic, Albanian, Farsi, Kurdish, Russian, Serbian, Somali
51	Civilian version of the PTSD Checklist (PCL-C)	Arnetz 2014 Javanbakht 2019 LeMaster 2018	PTSD	CHW	Arabic
52	Posttraumatic Diagnostic Scale (PDS)	Kroger 2016 Mewes 2018 Wulfes 2019	PTSD	MHS, Self-assessment	German, Arabic, Persian, Kurdish, Turkish, English
53	Patient Health Questionnaire (PHQ-2)	Kroger 2016 Nikendei 2019 Poole 2020	Depression	Trained research assistants	English, German, French, Persian, Arabic, Turkish, Kurmanji (Northern Kurdish), Urdu, Hausa, Russian, Serbian, Albanian, Macedonian, Georgian, Mandinka, Tigrinya
54	Patient Health Questionnaire (PHQ-8)	Poole 2020	Depression	Research personnel	
55	Patient Health Questionnaire (PHQ-9)	Bertelsen 2018 Bjarta 2018 Churbaji 2020 Mewes 2018 Wulfes 2019	Depression	MHS, PCP, Self-assessment	Arabic, Dari, Farsi, English, Kurdish
56	Patient Health Questionnaire—Panic Disorders (PHQ-PD)	Nikendei 2019	Panic Symptoms	Trained research assistants	English, German, French, Persian, Arabic, Turkish, Kurmanji (Northern Kurdish), Urdu, Hausa, Russian, Serbian, Albanian, Macedonian, Georgian, Mandinka, Tigrinya
57	Parentification Inventory (PI)	El Ghaziri 2019	OTHER	MHS, PCP, CHW	unspecified
58	Post-Migration Living Difficulties Checklist (PMLDC)	Schweitzer 2011	OTHER	CHW	unspecified
59	Process of Recognition and Orientation of Torture Victims in European Countries to Facilitate Care and Treatment (PROTECT) Questionnaire	Mewes 2018 Wulfes 2019	PTSD, Depression	MHS, PCP, LAY	English, German, Farsi, French, Persian, Arabic, Turkish, Kurdish, Kurmanji (Northern Kurdish), Urdu, Hausa, Russian, Serbian, Albanian, Macedonian, Georgian, Mandinka, Tigrinya
60	Present State Examination (PSE)	Hauff 1995	PTSD, psychiatric disorders	The first author	unspecified
61	Providing Online Resource and Trauma Assessment’ (PORTA)	Sukale 2017	Trauma, Depression, Anxiety, Behavioural Problems, Self- Harm/Suicidality	Self-administered	German, English, French, Arabic, Dari/Farsi, Pashto, Tigrinja, Somali
62	PTSD Symptoms Scale (PSS)	Weine 1998	PTSD	MHS, CHW, LAY	Croatian
63	Posttraumatic Symptom Scale (PTSS-10-70)	Thulesius 1999	PTSD	unspecified	English, Serbo-Croatian
64	Reactions of Adolescents on Traumatic Stress (RATS)	van Os 2018	PTSD	unspecified	Arabic, Dari, Farsi, Somali
65	Resilience Scale	LeMaster 2018	OTHER	CHW	unspecified
66	Refugee Health Screener-13 (RHS-13)	Bjarta 2018 Kaltenbach 2017	Depression, Anxiety, SOM, PTSD, OTHER	MHS	Amharic, Arabic, Albanian, Burmese, Cuban Spanish, Farsi, French, Haitian Creole, Karen, Kurdish, Mexican Spanish, Nepali, Russian, Spanish, Serbian, Somali, Swahili, Tigrinya
67	Refugee Health Screener-15 (RHS-15)	Al-Obaidi 2015 Baird 2020 Hollifield 2013 Hollifield 2016 Johnson-Agbakwu 2014 Kaltenbach 2017 Polcher 2016 Stingl 2019 Yalim 2020	Depression, Anxiety, Somatoform Disorders, PTSD, OTHER	MHS, PCP, CHW	Amharic, Arabic, Albanian, Burmese, Cuban, English, Farsi, French, Haitian Creole, Karen, Kurdish, Mexican Spanish, Nepali, Russian, Spanish, Serbian, Somali, Swahili, Tigrinya
68	Refugee Trauma History Checklist (RTHC)	Sigvardsdotter 2017	OTHER	MHS, PCP	English, Arabic
69	Savin et al. author-developed checklist derived from the DSM-IV	Savin 2005	Depression, Anxiety, PTSD	PCP, MHS	English
70	S-DAS	El Ghaziri 2019	Disruptive Behaviour Disorders, OTHER	MHS, PCP, CHW	unspecified
71	Structured Clinical Interview for DSM-IV (SCID)	Brink 2016 Tay 2013 Wulfes 2019	Depression, Anxiety, PTSD, Substance Use Disorders, Disruptive Behaviour Disorders, Somatoform Disorders	MHS, PCP	Danish, French, German, Greek, Hebrew, Karen, Italian, Portuguese, Spanish, Swedish, Turkish, Zulu
72	SCL-90-R	Hauff 1995 Weine 1998	Depression, Anxiety, Somatoform Disorders, Disruptive Behaviour Disorders, OTHER	MHS, PCP, CHW	Croatian
73	Strength and Difficulty Questionnaire (SDQ)	Green 2021 Hanes 2017 van Os 2018	OTHER	CHW, LAY	75+ languages
74	SF-10	El Ghaziri 2019	Depression, Anxiety, Disruptive Behaviour Disorders	MHS, PCP, CHW	unspecified
75	SF-12	El Ghaziri 2019	Depression, Anxiety, Disruptive Behaviour Disorders	MHW, PCP, CHW	unspecified
76	Shannon et al. author-developed questionnaire	Shannon 2015	Depression, PTSD, OTHER	Master’s-level and doctoral-level research assistant, professional Karen interpreters	English and Karen
77	Stressful Life Events (SLE)	van Os 2018	OTHER	unspecified	Arabic, Dari, Farsi, Somali
78	Sondergaard et al. (2001) author-developed questionnaire	Sondergaard 2001	PTSD, Anxiety, Depression, OTHER	Professionals working with refugees	Arabic and Sorani
79	STAR-MH	Hocking 2018	Depression, PTSD	CHW	English
80	Trauma Exposure Questionnaire by Nickerson et al.	Barbieri 2019	PTSD	PCP	Arabic, English, French
81	Vietnamese Depression Scale (VDS)	Buchwald 1995	Depression	PCP	Vietnamese
82	WHO-5	Nikendei 2019 Richter 2015	Social Well-Being	LAY, MHS, PCP	English, German, French, Persian, Arabic, Turkish, Kurmanji (Northern Kurdish), Urdu, Hausa, Russian, Serbian, Albanian, Macedonian, Georgian, Mandinka, Tigrinya
83	WHO General Health Questionnaire	Masmas 2008	Psychological Health Status	PCP, MHS	unspecified
84	World Health Organization Quality of Life Brief Version (WHOQOL-Bref)	Bjarta 2018	OTHER	MHS, PCP, CHW, LAY, SA	28 languages (who.int)
85	World Health Organization PTSD screener	Lillee 2015	PTSD	unspecified	English, Kurdish, Pashto

Legend: PCP: Primary care provider. MHS: Mental health specialist. CHW: Community health worker. LAY: Layperson. SA: Self-administration.

## Data Availability

Not applicable.

## Supplementary Materials

The following supporting information can be downloaded at: https://www.mdpi.com/article/10.3390/ijerph19063549/s1, Supplementary File S1: List of resettlement states; Supplementary File S2: PRISMA-ScR; Supplementary File S3: PRISMA-S; Supplementary File S4: Search strategies; Supplementary File S5: Excluded studies with reasons.
