# Supplementary material for: Mental Health Screening Approaches for Resettling Refugees and Asylum Seekers: A Scoping Review
Source: Int J Environ Res Public Health. 2022 Mar 16;19(6):3549. doi: 10.3390/ijerph19063549 (PMC8953108; doi:10.3390/ijerph19063549)
Supplement: Supplementary file 1 [file ijerph-19-03549-s001.zip › Supplementary File S1_ List of resettlement states.pdf]

Appendix 1: List of resettlement states

The UNHCR Resettlement Handbook offers resettlement management and policy guidance to UNHCR staff, and is a key reference tool for resettlement states and NGOs on global resettlement policy and practice. First released in 1997, the Resettlement Handbook was fully revised in 2011. Country Chapters are written by Resettlement States, identified as:

1. Argentina (June 2013 revision)
2. Australia (May 2018 revision)
3. Belgium (2018 revision)
4. Brazil (July 2013 revision)
5. Bulgaria (2018 revision)
6. Canada (2018 revision)
7. Chile (January 2002 revision)
8. Czech Republic (July 2018 revision)
9. Denmark (March 2016 revision)
10. Finland (October 2018 revision)
11. France (November 2018 revision)
12. Germany (2018 revision)
13. Iceland (April 2016 revision)
14. Ireland (December 2018 revision)
15. Italy (September 2019)
16. Netherlands (2018 revision)
17. New Zealand (May 2018 revision)
18. Norway (2021 revision)
19. Portugal (January 2019 revision)
20. Romania (August 2016)
21. Sweden (2018 revision)
22. United Kingdom (2018 revision)
23. United States of America (2018 revision)
24. Uruguay (July 2016 revision)

For more information, please see the UNHCR Resettlement Handbook and Country Chapters: https://www.unhcr.org/protection/resettlement/4a2ccf4c6/unhcr-resettlement-handbook-country-chapters.html
