# Supplementary material for: Mental Health Screening Approaches for Resettling Refugees and Asylum Seekers: A Scoping Review
Source: Int J Environ Res Public Health. 2022 Mar 16;19(6):3549. doi: 10.3390/ijerph19063549 (PMC8953108; doi:10.3390/ijerph19063549)
Supplement: Supplementary file 1 [file ijerph-19-03549-s001.zip › Supplementary File S4_ Search Strategies.pdf]

Appendix 4: Search Strategies

Medline	Embase	PsycINFO	Cochrane CENTRAL	CINAHL
1. (refugee* or migrant* or resettle* or immigra* or undocumented or newcomer*).ti,ab,kw.	1. (refugee* or migrant* or resettle* or immigra* or undocumented or newcomer*).ti,ab,kw.	(refugee* or migrant* or resettle* or immigra* or undocumented or newcomer*).ti,ab,id.	(refugee* or migrant* or resettle* or immigra* or undocumented or newcomer*).ti,ab,kw.	TI ((refugee* or migrant* or resettle* or immigra* or undocumented or newcomer*)) OR AB ((refugee* or migrant* or resettle* or immigra* or undocumented or newcomer*))
2. (asylum adj1 seeker).ti,ab,kw.	2. (asylum adj1 seeker).ti,ab,kw.	(asylum adj1 seeker).ti,ab,id.	(asylum adj1 seeker).ti,ab,kw.	TI (asylum N1 seeker) OR AB (asylum N1 seeker)
3. (Forc* adj2 (immigrant* or migrant* or migration or displace*)).ti,ab,kw.	3. (Forc* adj2 (immigrant* or migrant* or migration or displace*)).ti,ab,kw.	(Forc* adj2 (immigrant* or migrant* or migration or displace*)).ti,ab,id.	(Forc* adj2 (immigrant* or migrant* or migration or displace*)).ti,ab,kw.	TI ((Forc* N2 (immigrant* or migrant* or migration or displace*))) OR AB ((Forc* N2 (immigrant* or migrant* or migration or displace*)))
4. ((Undocumented or irregular) adj2 (immigrant* or migrant* or migration)).ti,ab,kw.	4. ((Undocumented or irregular) adj2 (immigrant* or migrant* or migration)).ti,ab,kw.	((Undocumented or irregular) adj2 (immigrant* or migrant* or migration)).ti,ab,id.	((Undocumented or irregular) adj2 (immigrant* or migrant* or migration)).ti,ab,kw.	TI (((Undocumented or irregular) N2 (immigrant* or migrant* or migration))) OR AB (((Undocumented or irregular) N2 (immigrant* or migrant* or migration)))
5. exp Refugees/	5. Exp Refugee/	Exp Refugees/	exp Refugees/	(MH "Refugees+")
6. exp "Transients and Migrants"/	6. Exp Migrant/	Exp asylum seeking/	exp "Transients and Migrants"/	(MH "Immigrants+")
7. "Emigration and Immigration"/	7. Exp Migration/	Exp immigration/	exp "Emigration and Immigration"/	(MH "Emigration and Immigration")
8. or/1-7	8. Or/ 1-7	Or/ 1-7	1 or 2 or 3 or 4 or 5 or 6 or 7	S1 OR S2 OR S3 OR S4 OR S5 OR S6 OR S7
9. (screen or screening or test or tests or tool or tools or instrument* or assess* or evaluation* or strateg* or diagnos* or protocol* or exam or exams or examination* or checklist*).ti,ab.	9. (screen or screening or test or tests or tool or tools or instrument* or assess* or evaluation* or strateg* or diagnos* or protocol* or exam or exams or examination* or checklist*).ti,ab.	(screen or screening or test or tests or tool or tools or instrument* or assess* or evaluation* or strateg* or diagnos* or protocol* or exam or exams or examination* or checklist*).ti,ab.	(screen or screening or test or tests or tool or tools or instrument* or assess* or evaluation* or strateg* or diagnos* or protocol* or exam or exams or examination* or checklist*).ti,ab,kw.	TI ((screen or screening or test or tests or tool or tools or instrument* or assess* or evaluation* or strateg* or diagnos* or protocol* or exam or exams or examination* or checklist*)) OR AB ((screen or screening or test or tests or tool or tools or instrument* or assess* or evaluation* or strateg* or diagnos* or protocol* or exam or exams or examination* or checklist*))
10. Symptom assessment/	10. Symptom assessment/	Exp symptom checklists/<br>Exp psychological assessment/	Symptom Assessment/	(MH "Clinical Assessment Tools") OR (MH "Mental Health Screening (Saba CCC)"
11. 9 or 10	11. 9 or 10	9 or 10 or 11	9 or 10	S9 OR S10
12. ((mental adj1 health) or anxiet* or anxious* or depress* or ptsd or posttrauma* or post-trauma* or trauma or suicid* or bipolar* or psycholog* or psychiatr*).ti,ab,kw.	12. ((mental adj1 health) or anxiet* or anxious* or depress* or ptsd or posttrauma* or post-trauma* or trauma or suicid* or bipolar* or psycholog* or psychiatr*).ti,ab,kw.	((mental adj1 health) or anxiet* or anxious* or depress* or ptsd or posttrauma* or post-trauma* or trauma or suicid* or bipolar* or psycholog* or psychiatr*).ti,ab,id.	((mental adj1 health) or anxiet* or anxious* or depress* or ptsd or posttrauma* or post-trauma* or trauma or suicid* or bipolar* or psycholog* or psychiatr*).ti,ab,kw.	TI (((mental N1 health) or anxiet* or anxious* or depress* or ptsd or posttrauma* or post-trauma* or trauma or suicid* or bipolar* or psycholog* or psychiatr*)) OR AB (((mental N1 health) or anxiet* or anxious* or depress* or ptsd or posttrauma* or post-trauma* or trauma or suicid* or bipolar* or psycholog* or psychiatr*))
13. Mental Health/ or Depression/ or Anxiety/ or Stress disorder, post-traumatic/ or psychological distress/ or exp suicide/ or suicidal ideation/ or exp Mental Disorders/	13. Mental Health/ or Depression/ or Anxiety/ or exp posttraumatic stress disorder/ or exp distress syndrome/ or exp suicide/ or exp suicidal behavior/ or exp Mental disease/	Exp Mental Health/ or exp Depression/ or exp anxiety/ or exp anxiety disorders/ or exp posttraumatic stress disorder/ or exp psychological stress/ or exp suicide/ or exp mental disorders/	mental health/ or Depression/ or Anxiety/ or Stress Disorder, Post-Traumatic/ or exp Stress, Psychological/ or exp suicide/ or exp mental disorders/	(MH "Mental Health") OR (MH "Depression") OR (MH "Anxiety") OR (MH "Stress Disorder, Post-Traumatic") OR (MH "Stress, Psychological+) OR (MH "suicide+") OR (MH "mental disorders+")
14. 12 or 13	14. 12 or 13	12 or 13	12 or 13	S12 OR S13
15. 8 and 11 and 14	15. 8 and 11 and 14	8 and 11 and 14	8 and 11 and 14	S8 AND S11 AND S14 Limiters - Published Date: 19950101-20210131
16. limit 15 to yr="1995 -Current"	16. limit 15 to yr="1995 -Current"	limit 15 to yr="1995 -Current"	limit 15 to yr="1995 -Current"	

RESULTS	RESULTS	RESULTS	RESULTS	RESULTS
Date of search: 22 December 2020<br>Database: Ovid MEDLINE(R) ALL <1946 to December 21, 2020><br><br>1 (refugee* or migrant* or resettle* or immigra* or undocumented or newcomer*).ti,ab,kw. (63929)<br>2 (asylum adj1 seeker).ti,ab,kw. (227)<br>3 (Forc* adj2 (immigrant* or migrant* or migration or displace*)).ti,ab,kw. (3010)<br>4 ((Undocumented or irregular) adj2 (immigrant* or migrant* or migration)).ti,ab,kw. (872)<br>5 exp Refugees/ (10556)<br>6 exp "Transients and Migrants"/ (11965)<br>7 "Emigration and Immigration"/ (25319)<br>8 or/1-7 (84823)<br>9 (screen or screening or test or tests or tool or tools or instrument* or assess* or evaluation* or strateg* or diagnos* or protocol* or exam or exams or examination* or checklist*).ti,ab. (9162885)<br>10 Symptom assessment/ (5435)<br>11 9 or 10 (9164234)<br>12 ((mental adj1 health) or anxiet* or anxious* or depress* or ptsd or posttrauma* or post-trauma* or trauma or suicid* or bipolar* or psycholog* or psychiatr*).ti,ab,kw. (1390982)<br>13 Mental Health/ or Depression/ or Anxiety/ or Stress disorder, post-traumatic/ or psychological distress/ or exp suicide/ or suicidal ideation/ or exp Mental Disorders/ (1434058)<br>14 12 or 13 (2277968)<br>15 8 and 11 and 14 (6542)<br>16 limit 15 to yr="1995 -Current" (6051)	Date of Search: 27 December 2020<br>Database: Embase <1974 to 2020 December 24><br><br>1 (refugee* or migrant* or resettle* or immigra* or undocumented or newcomer*).ti,ab,kw. (72506)<br>2 (asylum adj1 seeker).ti,ab,kw. (391)<br>3 (Forc* adj2 (immigrant* or migrant* or migration or displace*)).ti,ab,kw. (3411)<br>4 ((Undocumented or irregular) adj2 (immigrant* or migrant* or migration)).ti,ab,kw. (996)<br>5 exp Refugee/ (13804)<br>6 exp Migrant/ (39229)<br>7 exp Migration/ (45312)<br>8 or/1-7 (107657)<br>9 (screen or screening or test or tests or tool or tools or instrument* or assess* or evaluation* or strateg* or diagnos* or protocol* or exam or exams or examination* or checklist*).ti,ab. (12315280)<br>10 Symptom assessment/ (8161)<br>11 9 or 10 (12316994)<br>12 ((mental adj1 health) or anxiet* or anxious* or depress* or ptsd or posttrauma* or post-trauma* or trauma or suicid* or bipolar* or psycholog* or psychiatr*).ti,ab,kw. (1837053)<br>13 Mental Health/ or Depression/ or Anxiety/ or exp posttraumatic stress disorder/ or exp distress syndrome/ or exp suicide/ or exp suicidal behavior/ or exp Mental disease/ (2396907)<br>14 12 or 13 (3289391)<br>15 8 and 11 and 14 (9159)<br>16 limit 15 to yr="1995 -Current" (8656)	Date of search: 29 December 2020<br>Database: APA PsycInfo <1806 to December Week 3 2020><br>1 (refugee* or migrant* or resettle* or immigra* or undocumented or newcomer*).ti,ab,id. (50344)<br>2 (asylum adj1 seeker).ti,ab,id. (240)<br>3 (Forc* adj2 (immigrant* or migrant* or migration or displace*)).ti,ab,id. (746)<br>4 ((Undocumented or irregular) adj2 (immigrant* or migrant* or migration)).ti,ab,id. (901)<br>5 exp Refugees/ (6404)<br>6 exp asylum seeking/ (643)<br>7 exp immigration/ (22978)<br>8 or/1-7 (51894)<br>9 (screen or screening or test or tests or tool or tools or instrument* or assess* or evaluation* or strateg* or diagnos* or protocol* or exam or exams or examination* or checklist*).ti,ab. (1967740)<br>10 exp symptom checklists/ (811)<br>11 exp psychological assessment/ (109843)<br>12 9 or 10 or 11 (1995993)<br>13 ((mental adj1 health) or anxiet* or anxious* or depress* or ptsd or posttrauma* or post-trauma* or trauma or suicid* or bipolar* or psycholog* or psychiatr*).ti,ab,id. (1320454)<br>14 exp Mental Health/ or exp Depression/ or exp anxiety/ or exp anxiety disorders/ or exp posttraumatic stress disorder/ or exp psychological stress/ or exp suicide/ or exp mental disorders/ (1014623)<br>15 13 or 14 (1776057)<br>16 8 and 12 and 15 (7511)<br>17 limit 16 to yr="1995 -Current" (6892)	Date of search: 15 January 2020<br>Database: Cochrane Central Register of Controlled Trials <1991 to January Week 2 2021><br>1 (refugee* or migrant* or resettle* or immigra* or undocumented or newcomer*).ti,ab,kw. (1743)<br>2 (asylum adj1 seeker).ti,ab,kw. (19)<br>3 (Forc* adj2 (immigrant* or migrant* or migration or displace*)).ti,ab,kw. (114)<br>4 ((Undocumented or irregular) adj2 (immigrant* or migrant* or migration)).ti,ab,kw. (8)<br>5 exp Refugees/ (115)<br>6 exp "Transients and Migrants"/ (71)<br>7 exp "Emigration and Immigration"/ (64)<br>8 1 or 2 or 3 or 4 or 5 or 6 or 7 (1886)<br>9 (screen or screening or test or tests or tool or tools or instrument* or assess* or evaluation* or strateg* or diagnos* or protocol* or exam or exams or examination* or checklist*).ti,ab,kw. (948121)<br>10 Symptom Assessment/ (238)<br>11 9 or 10 (948166)<br>12 ((mental adj1 health) or anxiet* or anxious* or depress* or ptsd or posttrauma* or post-trauma* or trauma or suicid* or bipolar* or psycholog* or psychiatr*).ti,ab,kw. (176680)<br>13 mental health/ or Depression/ or Anxiety/ or Stress Disorder, Post-Traumatic/ or exp Stress, Psychological/ or exp suicide/ or exp mental disorders/ (85313)<br>14 12 or 13 (217701)<br>15 8 and 11 and 14 (472)<br>16 limit 15 to yr="1995 -Current" (471)	Date of search: 15 January 2020<br>Database: CINAHL <1981 to January Week 2 2021><br>TI ((refugee* or migrant* or resettle* or immigra* or undocumented or newcomer*)) OR AB ((refugee* or migrant* or resettle* or immigra* or undocumented or newcomer*)) (27931)<br>2. TI (asylum N1 seeker) OR AB (asylum N1 seeker) (915)<br>3. TI ((Forc* N2 (immigrant* or migrant* or migration or displace*))) OR AB ((Forc* N2 (immigrant* or migrant* or migration or displace*))) (743)<br>4. TI (((Undocumented or irregular) N2 (immigrant* or migrant* or migration))) OR AB (((Undocumented or irregular) N2 (immigrant* or migrant* or migration))) (631)<br>5. (MH "Refugees+") (7505)<br>6. (MH "Immigrants+") (14996)<br>7. (MH "Emigration and Immigration") (6701)<br>8. S1 OR S2 OR S3 OR S4 OR S5 OR S6 OR S7 (38475)<br>9. TI ((screen or screening or test or tests or tool or tools or instrument* or assess* or evaluation* or strateg* or diagnos* or protocol* or exam or exams or examination* or checklist*)) OR AB ((screen or screening or test or tests or tool or tools or instrument* or assess* or evaluation* or strateg* or diagnos* or protocol* or exam or exams or examination* or checklist*)) (2073321)<br>10. (MH "Clinical Assessment Tools") OR (MH "Mental Health Screening (Saba CCC)") (166014)<br>11. S9 OR S10 (2137518)<br>12. TI (((mental N1 health) or anxiet* or anxious* or depress* or ptsd or posttrauma* or post-trauma* or trauma or suicid* or bipolar* or psycholog* or psychiatr*)) OR AB (((mental N1 health) or anxiet* or anxious* or depress* or ptsd or posttrauma* or post-trauma* or trauma or suicid* or bipolar* or psycholog* or psychiatr*)) (517945)<br>13. (MH "Mental Health") OR (MH "Depression") OR (MH "Anxiety") OR (MH "Stress Disorder, Post-Traumatic") OR (MH "Stress, Psychological+) OR (MH "suicide+") OR (MH "mental disorders+") (695484)<br>14. S12 OR S13 (956890)<br>15. S8 AND S11 AND S14 Limiters - Published Date: 19950101-20210131 (3787)
