# Supplementary material for: Mental Health Screening Approaches for Resettling Refugees and Asylum Seekers: A Scoping Review
Source: Int J Environ Res Public Health. 2022 Mar 16;19(6):3549. doi: 10.3390/ijerph19063549 (PMC8953108; doi:10.3390/ijerph19063549)
Supplement: Supplementary file 1 [file ijerph-19-03549-s001.zip › Supplementary File S5_ Excluded studies.pdf]

Authors	Published Year	Title	Journal	Volume	Issue	Pages	DOI	Notes
Abdalla, Kirsten; Elklit, Ask	2001	A nation-wide screening of refugee children from Kosovo.	Psykologisk Paedagogisk Radgivning	38	5	339-348		Exclusion reason: Full text unavailable;
Acarturk, Ceren; McGrath, Michael; Roberts, Bayard; Ilkkursun, Zeynep; Cuijpers, Pim; Sijbrandij, Marit; Sondorp, Egbert; Ventevogel, Peter; McKee, Martin; Fuhr, Daniela C; STRENGTHS consortium	2020	Prevalence and predictors of common mental disorders among Syrian refugees in Istanbul, Turkey: a cross-sectional study.	Social psychiatry and psychiatric epidemiology		uvp, 8804358		https://dx.doi.org/	Exclusion reason: Ineligible study design;
Achenbach, Thomas M; Becker, Andreas; Dopfner, Manfred; Heiervang, Einar; Roessner, Veit; Steinhausen, Hans-Christoph; Rothenberger, Aribert	2008	Multicultural assessment of child and adolescent psychopathology with ASEBA and SDQ instruments: research findings, applications, and future directions.	Journal of child psychology and psychiatry, and allied disciplines	49	3	251-75	https://dx.doi.org/	Exclusion reason: Population is not refugee/asylum seeker;
Acquaye, Hannah E	2018	Assessing the factor structure of models for posttraumatic stress disorder symptoms in a war-related civilian sample.	Counseling Outcome Research and Evaluation	9	2	90-101	http://dx.doi.org/1	Exclusion reason: Not along resettlement pathway;
Ahmad F, Shakya Y, Li J, Khoaja K, Norman CD, Lou W, Abuelaish I, Ahmadzi HM	2012	A pilot with computer-assisted psychosocial risk-assessment for refugees		12		71		Exclusion reason: Not along resettlement pathway;
Ahmad, A; Sundelin-Wahlsten, V; Sofi, M A; Qahar, J A; von Knorring, A L	2000	Reliability and validity of a child-specific cross-cultural instrument for assessing posttraumatic stress disorder.	European child & adolescent psychiatry	9	4	285-94		Exclusion reason: Not along resettlement pathway;
Ahmad, Abdulbaghi; Mohamed, Hawar T; Ameen, Nazar M	1998	A 26-month follow-up of posttraumatic stress symptoms in children after the mass-escape tragedy in Iraqi Kurdistan.	Nordic Journal of Psychiatry	52	5	357-366	http://dx.doi.org/1	Exclusion reason: Not along resettlement pathway;
Akgul, Sinem; Husnu, Senel; Derman, Orhan; Ozmert, Elif; Bideci, Aysun; Hasanoglu, Enver	2019	Mental health of Syrian refugee adolescents: how far have we come?.	The Turkish journal of pediatrics	61	6	839-845	https://dx.doi.org/	Exclusion reason: Not along resettlement pathway;
Al-Amer, Rasmieh; Maneze, Della; Ramjan, Lucie; Villarosa, Amy R; Darwish, Rima; Salamonson, Yenna	2020	Psychometric testing of the Arabic version of the Patient Health Questionnaire among adolescent refugees living in Jordan.	International journal of mental health nursing	29	4	685-692	https://dx.doi.org/	Exclusion reason: Not along resettlement pathway;
Andersen, Mathilde Horn; Kruse, Alexandra; Frederiksen, Hanne Winther; Ahmadi, Afsaneh; Norredam, Marie	2020	Health status of refugees newly resettled in Denmark.	Danish medical journal	67	12			Exclusion reason: Ineligible study design;
Ao, Trong; Shetty, Sharmila; Sivilli, Teresa; Blanton, Curtis; Ellis, Heidi; Geltman, Paul L; Cochran, Jennifer; Taylor, Eboni; Lankau, Emily W; Lopes Cardozo, Barbara	2016	Suicidal Ideation and Mental Health of Bhutanese Refugees in the United States.	Journal of immigrant and minority health	18	4	828-835	https://dx.doi.org/	Exclusion reason: Ineligible study design;
Aoun, A.; Joundi, J.; El Gerges, N.; El Jabbour, F.; El Osta, L.		SUN-P160: Eating Disorders and Post-Traumatic Stress Disorder Among Syrian Refugees in North Lebanon: Screening and Correlation.	Clinical Nutrition	36		S113-S113	10.1016/S0261-5	Exclusion reason: Ineligible study design;
Arfken, Cynthia L; Alsaud, Mohammed; Mischel, Edward F; Haddad, Luay; Sonderman, Samantha; Lister, Jamey J; Javanbakht, Arash	2018	Recent Iraqi refugees: Association between ethnic identification and psychological distress.	Journal of Muslim Mental Health	12	2	1-12		Exclusion reason: Ineligible study design;
Bailliet, Cecilia M	2009	Towards the restoration of individualized assessment of mental health in refugee law.	Tidsskrift for Norsk Psykologforening	46	12	1163-1168		Exclusion reason: Ineligible study design;
Banerjee T.; Ajmal S.; Khan A.; Arora R.	2019	Health needs of unaccompanied asylum seeker children-observations from initial health assessment in community paediatric clinic	Archives of Disease in Childhood	104	Supplement 2	A212	http://dx.doi.org/1	Exclusion reason: Full text unavailable;
Bapolisi, Achille Mwira; Song, Suzan J; Kesande, Claire; Rukundo, Godfrey Zari; Ashaba, Scholastic	2020	Post-traumatic stress disorder, psychiatric comorbidities and associated factors among refugees in Nakivale camp in southwestern Uganda.	BMC psychiatry	20	1	53	https://dx.doi.org/	Exclusion reason: Not along resettlement pathway;
Baranowski, Kim A	2020	Documenting human rights violations: An introduction to the psychological evaluation of asylum seekers.	Practice Innovations	5	1	32-44	http://dx.doi.org/1	Exclusion reason: Not a screening program;
Baranowski, Kim A; Moses, Melissa H; Sundri, Jasmine	2018	Supporting Asylum Seekers: Clinician Experiences of Documenting Human Rights Violations Through Forensic Psychological Evaluation.	Journal of traumatic stress	31	3	391-400	https://dx.doi.org/	Exclusion reason: Duplicate;
Barthel, Dana; Ravens-Sieberer, Ulrike; Schulte-Markwort, Michael; Klasen, Fionna; Zindler, Areej	2019	The clinical-psychological diagnostic process in an outpatient health-care center for refugee children and adolescents: First results of mental health problems, traumatic events, and treatment goals.	Kindheit und Entwicklung: Zeitschrift fur Klinische Kinderpsychologie	28	3	160-172	http://dx.doi.org/1	Exclusion reason: Not a screening program;
Basheti, Iman A; Ayasrah, Shahnaz M; Basheti, Mariam M; Mahfuz, Judeh; Chaar, Betty	2019	The Syrian refugee crisis in Jordan: a cross sectional pharmacist-led study assessing post-traumatic stress disorder.	Pharmacy practice	17	3	1475	https://dx.doi.org/	Exclusion reason: Not along resettlement pathway;
Bastin, Pierre; Bastard, Mathieu; Rossel, Ludovic; Melgar, Pablo; Jones, Alison; Antierens, Annick	2013	Description and predictive factors of individual outcomes in a refugee camp based mental health intervention (Beirut, Lebanon).	PloS one	8	1	e54107	https://dx.doi.org/	Exclusion reason: Not along resettlement pathway;

Authors	Published Year	Title	Journal	Volume	Issue	Pages	DOI	Notes
Bean, Tammy; Derluyn, Ilse; Eurelings-Bontekoe, Elisabeth; Broekaert, Eric; Spinhoven, Philip	2007	Validation of the multiple language versions of the Hopkins Symptom Checklist-37 for refugee adolescents.	Adolescence	42	165	51-71		Exclusion reason: Not along resettlement pathway;
Bean, Tammy; Mooijart, Ab; Eurelings-Bontekoe, Elisabeth; Spinhoven, Philip	2006	Validation of the child behavior checklist for guardians of unaccompanied refugee minors.	Children and Youth Services Review	28	8	867-887	http://dx.doi.org/1	Exclusion reason: Not along resettlement pathway;
Bell, Sue Anne; Lori, Jody; Redman, Richard; Seng, Julia	2015	Development of a brief screening tool for women's mental health assessment in refugee settings: A psychometric evaluation.	International journal of nursing studies	52	7	1202-8	https://dx.doi.org/	Exclusion reason: Not along resettlement pathway;
Bell, Sue Anne; Lori, Jody; Redman, Richard; Seng, Julia	2015	Psychometric Validation and Comparison of the Self-Reporting Questionnaire-20 and Self-Reporting Questionnaire-Suicidal Ideation and Behavior Among Congolese Refugee Women.	Journal of nursing measurement	23	3	393-408	https://dx.doi.org/	Exclusion reason: Not along resettlement pathway;
Belz, Maria; Belz, Michael; Ozkan, Ibrahim; Graef-Calliess, Iris T	2017	Posttraumatic stress disorder and comorbid depression among refugees: Assessment of a sample from a German refugee reception center.	Transcultural psychiatry	54	5-6	595-610	https://dx.doi.org/	Exclusion reason: No mental health screening;
Bertelsen N.S.; Selden E.; Krass P.; Keatley E.S.; Keller A.	2016	Primary care screening methods and outcomes among asylum seekers in New York City	Annals of Global Health	82	3	432		Exclusion reason: conference poster/ abstract;
Berthold, S Megan; Mollica, Richard F; Silove, Derrick; Tay, Alvin Kuowei; Lavelle, James; Lindert, Jutta	2019	HTQ-5: revision of the Harvard Trauma Questionnaire for measuring torture, trauma and DSM-5 PTSD symptoms in refugee populations.	European Journal of Public Health	29	3	468-474	10.1093/eurpub/c	Exclusion reason: Not a screening program;
Berthold, S Megan; Mollica, Richard F; Silove, Derrick; Tay, Alvin Kuowei; Lavelle, James; Lindert, Jutta	2019	The HTQ-5: revision of the Harvard Trauma Questionnaire for measuring torture, trauma and DSM-5 PTSD symptoms in refugee populations.	European journal of public health	29	3	468-474	https://dx.doi.org/	Exclusion reason: Duplicate;
Betancourt, Theresa S; Yudron, Monica; Wheaton, Wendy; Smith-Fawzi, Mary C	2012	Caregiver and adolescent mental health in Ethiopian Kunama refugees participating in an emergency education program.	The Journal of adolescent health : official publication of the Society for Adolescent Medicine	51	4	357-65	https://dx.doi.org/	Exclusion reason: Ineligible study design;
Biddle, Louise; Miners, Alec; Bozorgmehr, Kayvan	2019	Cost-utility of screening for depression among asylum seekers: a modelling study in Germany.	Health policy (Amsterdam, Netherlands)	123	9	873-881	https://dx.doi.org/	Exclusion reason: Ineligible study design;
Bishop D.; Altshuler M.; Scott K.; Panzer J.; Mills G.; McManus P.	2012	The refugee medical exam: What you need to do	Journal of Family Practice	61	12	E1-E10		Exclusion reason: Ineligible study design;
Blair, R G	2000	Risk factors associated with PTSD and major depression among Cambodian refugees in Utah.	Health & social work	25	1	23-30		Exclusion reason: Not along resettlement pathway;
Blight, Karin Johansson; Ekblad, Solvig; Persson, Jan-Olov; Ekberg, Jan	2006	Mental health, employment and gender. Cross-sectional evidence in a sample of refugees from Bosnia-Herzegovina living in two Swedish regions.	Social science & medicine (1982)	62	7	1697-709		Exclusion reason: Not along resettlement pathway;
Boyle, Jacqueline Anne; Willey, Suzanne; Blackmore, Rebecca; East, Christine; McBride, Jacqueline; Gray, Kylie; Melvin, Glenn; Fradkin, Rebecca; Ball, Natahl; Highet, Nicole; Gibson-Helm, Melanie	2019	Improving Mental Health in Pregnancy for Refugee Women: Protocol for the Implementation and Evaluation of a Screening Program in Melbourne, Australia.	Journal of Medical Internet Research	21	8	N.PAG-N.PAG	10.2196/13271	Exclusion reason: Ineligible study design;
Bradley, Lloyd; Tawfiq, Nouran	2006	The physical and psychological effects of torture in Kurds seeking asylum in the United Kingdom.	Torture : quarterly journal on rehabilitation of torture victims and prevention of torture	16	1	41-7		Exclusion reason: Not along resettlement pathway;
Brakemeier, Eva-Lotta; Zimmermann, Johannes; Erz, Elina; Bollmann, Simon; Rump, Simon; von Kempski, Viktoria; Grossmuller, Tanita; Mitelman, Avija; Gehrisch, Johanna; Spies, Jan; Storck, Timo; Schouler-Ocak, Meryam	2017	Interpersonal integrative pilot project for refugees with mental disorders. Presentation of the project and initial results on feasibility and outcome.	Psychotherapeut	62	4	322-332	http://dx.doi.org/1	Exclusion reason: Full text unavailable;
Briggs, Lynne	2011	Demoralization and psychological distress in refugees: From research to practice.	Social Work in Mental Health	9	5	336-345	http://dx.doi.org/1	Exclusion reason: Not a screening program;
Bronstein, Israel; Montgomery, Paul; Dobrowolski, Stephanie	2012	PTSD in asylum-seeking male adolescents from Afghanistan.	Journal of traumatic stress	25	5	551-7	https://dx.doi.org/	Exclusion reason: Ineligible study design;
Bronstein, Israel; Montgomery, Paul; Ott, Eleanor	2013	Emotional and behavioural problems amongst Afghan unaccompanied asylum-seeking children: results from a large-scale cross-sectional study.	European child & adolescent psychiatry	22	5	285-94	https://dx.doi.org/	Exclusion reason: Ineligible study design;
Brown, Carina M; Swaminathan, Lalitha; Saif, Nadia T; Hauck, Fern R	2020	Health Care for Refugee and Immigrant Adolescents.	Primary care	47	2	291-306	https://dx.doi.org/	Exclusion reason: Not a screening program;
Brown, Chris; Schale, Codi L; Nilsson, Johanna E	2010	Vietnamese immigrant and refugee women's mental health: An examination of age of arrival, length of stay, income, and English language proficiency.	Journal of Multicultural Counseling and Development	38	2	66-76	http://dx.doi.org/1	Exclusion reason: Not along resettlement pathway;
Bryant, Richard A; Edwards, Ben; Creamer, Mark; O'Donnell, Meaghan; Forbes, David; Felmingham, Kim L; Silove, Derrick; Steel, Zachary; McFarlane, Alexander C; Van Hooff, Miranda; Nickerson, Angela; Hadzi-Pavlovic, Dusan	2020	Prolonged grief in refugees, parenting behaviour and children's mental health.	The Australian and New Zealand journal of psychiatry		9i6, 0111052	4867420967420	https://dx.doi.org/	Exclusion reason: Not along resettlement pathway;

Authors	Published Year	Title	Journal	Volume	Issue	Pages	DOI	Notes
Bryant, Richard A; Edwards, Ben; Creamer, Mark; O'Donnell, Meaghan; Forbes, David; Felmingham, Kim L; Silove, Derrick; Steel, Zachary; Nickerson, Angela; McFarlane, Alexander C; Van Hooff, Miranda; Hadzi-Pavlovic, Dusan	2018	The effect of post-traumatic stress disorder on refugees' parenting and their children's mental health: a cohort study.	The Lancet. Public health	3	5	e249-e258	https://dx.doi.org/	Exclusion reason: Not a screening program;
Buchmuller, Thimo; Lembcke, Hanna; Ialuna, Francesca; Busch, Julian; Leyendecker, Birgit	2020	Mental Health Needs of Refugee Children in Specialized Early Education and Care Programs in Germany.	Journal of immigrant and minority health	22	1	22-33	https://dx.doi.org/	Exclusion reason: Not a screening program;
Buljan D.; Vrcek D.; Cekic-Arambasin A.; Karlovic D.; Zoricic Z.; Golik-Gruber V.	2002	Posttraumatic stress disorder, alcohol dependence, and somatic disorders in displaced persons	Alcoholism	38	1-2	35-40		Exclusion reason: Not along resettlement pathway;
Cantekin, Duygu; Gencoz, Tulin	2017	Mental health of Syrian asylum seekers in Turkey: The role of pre-migration and post-migration risk factors.	Journal of Social and Clinical Psychology	36	10	835-859	http://dx.doi.org/1	Exclusion reason: Full text unavailable;
Carlsson, Jessica M; Mortensen, Erik L; Kastrup, Marianne	2006	Predictors of mental health and quality of life in male tortured refugees.	Nordic journal of psychiatry	60	1	51-7		Exclusion reason: Ineligible study design;
Carlsson, Jessica Mariana; Mortensen, Erik Lykke; Kastrup, Marianne	2005	A follow-up study of mental health and health-related quality of life in tortured refugees in multidisciplinary treatment.	The Journal of nervous and mental disease	193	10	651-7		Exclusion reason: Not along resettlement pathway;
Cengiz I.; Ergun D.; Cakici E.	2019	Posttraumatic stress disorder, posttraumatic growth and psychological resilience in syrian refugees: Hatay, Turkey	Anadolu Psikiyatri Dergisi	20	3	269-276	http://dx.doi.org/1	Exclusion reason: Not along resettlement pathway;
Ceri, Veysi; Beser, Can; Perdahli Fis, Nese; Arman, Ayse	2018	Findings from a specialized child psychiatry unit for care of refugee children in Istanbul.	Klinik Psikiyatri Dergisi: The Journal of Clinical Psychiatry	21	2	113-121	http://dx.doi.org/1	Exclusion reason: Not along resettlement pathway;
Ceri, Veysi; Nasiroglu, Serhat; Ceri, Monika; Cetin, Fusun Cuhadaroglu	2018	Psychiatric morbidity among a school sample of Syrian refugee children in turkey: a cross-sectional, semistructured, standardized interview-based study.	Journal of the American Academy of Child & Adolescent Psychiatry	57	9	696-698	http://dx.doi.org/1	Exclusion reason: Not along resettlement pathway;
Ceri, Veysi; Ozlu-Erkilic, Zeliha; Ozer, Urun; Yalcin, Murat; Popow, Christian; Akkaya-Kalayci, Turkan	2016	Psychiatric symptoms and disorders among Yazidi children and adolescents immediately after forced migration following ISIS attacks.	Neuropsychiatrie : Klinik, Diagnostik, Therapie und Rehabilitation : Organ der Gesellschaft Osterreichischer Nervenarzte und Psychiater	30	3	145-150		Exclusion reason: Not along resettlement pathway
Cha, Jiho; Surkan, Pamela J; Kim, Jaeshin; Yoon, Isabel A; Robinson, Courtland; Cardozo, Barbara Lopes; Lee, Hayoung	2018	Human Rights as Political Determinants of Health: A Retrospective Study of North Korean Refugees.	American journal of preventive medicine	55	2	271-279	https://dx.doi.org/	Exclusion reason: Not along resettlement pathway;
Chaaya, M; Sibai, A M; Fayad, R; El-Roueiheb, Z	2007	Religiosity and depression in older people: evidence from underprivileged refugee and non-refugee communities in Lebanon.	Aging & mental health	11	1	37-44		Exclusion reason: Not along resettlement pathway;
Chang A.; Scherb H.; Causevic S.	2016	Analysis of refugee mental health screening and referral processes at the newcomers health program, San Francisco general hospital's refugee medical clinic: A quality improvement study	Annals of Global Health	82	3	409		Exclusion reason: Ineligible study design;
Charney, Meredith E; Keane, Terence M	2007	Psychometric analyses of the Clinician-Administered PTSD Scale (CAPS)--Bosnian translation.	Cultural diversity & ethnic minority psychology	13	2	161-168	https://dx.doi.org/	Exclusion reason: Not along resettlement pathway;
Chaves, Nadia J; Paxton, Georgia A; Biggs, Beverley-Ann; Thambiran, Aesen; Gardiner, Joanne; Williams, Jan; Smith, Mitchell M; Davis, Joshua S	2017	The Australasian Society for Infectious Diseases and Refugee Health Network of Australia recommendations for health assessment for people from refugee-like backgrounds: an abridged outline.	The Medical journal of Australia	206	7	310-315		Exclusion reason: Ineligible study design;
Chernet A.; Probst-Hensch N.; Sydow V.; Paris D.H.; Labhardt N.D.	2019	Mental health and resilience, on and post arrival among eritrean refugees in Switzerland: A mental health and resilience, on and post arrival among eritrean refugees in Switzerland-a cohort study	Transactions of the Royal Society of Tropical Medicine and Hygiene	113	Supplement 1	S290	http://dx.doi.org/1	Exclusion reason: Full text unavailable;
Cheung Chung, Man; AlQarni, Nowf; AlMazrouei, Mariam; Al Muhairi, Shamsa; Shakra, Mudar; Mitchell, Britt; Al Mazrouei, Sara; Al Hashimi, Shurooq	2018	The impact of trauma exposure characteristics on post-traumatic stress disorder and psychiatric co-morbidity among Syrian refugees.	Psychiatry research	259	qc4, 7911385	310-315	https://dx.doi.org/	Exclusion reason: Not along resettlement pathway;
Cleveland, Janet; Rousseau, Cecile	2013	Psychiatric symptoms associated with brief detention of adult asylum seekers in Canada.	Canadian journal of psychiatry. Revue canadienne de psychiatrie	58	7	409-16		Exclusion reason: Not a screening program;
Coyle R.; Bowen S.; Mullin S.; Sayer N.; Siggers G.; Bennett S.	2016	Physical and mental health needs of unaccompanied children seeking asylum: A descriptive analysis in Kent, UK	The Lancet	388	SPEC.ISS 1	40		Exclusion reason: Full text unavailable;
Crawshaw, A F; Kirkbride, H	2018	Public Health England's Migrant Health Guide: an online resource for primary care practitioners.	Public health	158	qi7, 0376507	198-202	https://dx.doi.org/	Exclusion reason: Population is not refugee/asylum seeker;

Authors	Published Year	Title	Journal	Volume	Issue	Pages	DOI	Notes
Crepet, Anna; Rita, Francesco; Reid, Anthony; Van den Boogaard, Wilma; Deiana, Pina; Quaranta, Gaia; Barbieri, Aurelia; Bongiorno, Francesco; Di Carlo, Stefano	2017	Mental health and trauma in asylum seekers landing in Sicily in 2015: a descriptive study of neglected invisible wounds.	Conflict and health	11	101286573	1	https://dx.doi.org/	Exclusion reason: Ineligible study design;
Cusack, Karen Jean	2002	Refugee experiences of trauma and PTSD; Effects on psychological, physical, and financial well-being.	Dissertation Abstracts International: Section B: The Sciences and Engineering	62	10-B	4778		Exclusion reason: Ineligible study design;
D'Avanzo, C E; Barab, S A	1998	Depression and anxiety among Cambodian refugee women in France and the United States.	Issues in mental health nursing	19	6	541-56		Exclusion reason: Not along resettlement pathway; ;
D'Souza R.F.	2011	The mental health consequences of protracted asylum seeking in Australia	European Psychiatry	26	SUPPL. 1		http://dx.doi.org/1	Exclusion reason: Full text unavailable;
Dao, Tam K; Poritz, Julia M P; Moody, Rachel P; Szeto, Kim	2012	Development, reliability, and validity of the Posttraumatic Stress Disorder Interview for Vietnamese refugees: a diagnostic instrument for Vietnamese refugees.	Journal of traumatic stress	25	4	440-5	https://dx.doi.org/	Exclusion reason: Not along resettlement pathway;
Davey, Carine; Heard, Robert; Lennings, Chris	2015	Development of the Arabic versions of the Impact of Events Scale-Revised and the Posttraumatic Growth Inventory to assess trauma and growth in Middle Eastern refugees in Australia.	Clinical Psychologist	19	3	131-139	http://dx.doi.org/1	Exclusion reason: Not a screening program;
Davidson, N; Skull, S; Chaney, G; Frydenberg, A; Jones, Cheryl; Isaacs, D; Kelly, P; Lampropoulos, B; Raman, S; Silove, D; Buttery, J; Smith, M; Steel, Z; Burgner, D	2004	Comprehensive health assessment for newly arrived refugee children in Australia.	Journal of paediatrics and child health	40	9-10	562-8		Exclusion reason: Ineligible study design;
Davis, Rebecca Meghan; Davis, Henry 4th	2006	PTSD symptom changes in refugees.	Torture : quarterly journal on rehabilitation of torture victims and prevention of torture	16	1	10-9		Exclusion reason: Full text unavailable;
De Andrade, Yaya Maria	1995	Psychosocial trauma: Dialogues with emigre children from El Salvador.	Dissertation Abstracts International: Section B: The Sciences and Engineering	56	5-B	2859		Exclusion reason: Not along resettlement pathway;
de Fouchier, Capucine; Blanchet, Alain; Hopkins, William; Bui, Eric; Ait-Aoudia, Malik; Jehel, Louis	2012	Validation of a French adaptation of the Harvard Trauma Questionnaire among torture survivors from sub-Saharan African countries.	European journal of psychotraumatology	3	101559025		https://dx.doi.org/	Exclusion reason: Screening program administered post 12months of settlement;
de Jong, J P; Scholte, W F; Koeter, M W; Hart, A A	2000	The prevalence of mental health problems in Rwandan and Burundese refugee camps.	Acta psychiatrica Scandinavica	102	3	171-7		Exclusion reason: Not along resettlement pathway;
De Vries, J	2001	Mental health issues in Tamil refugees and displaced persons. Counselling implications.	Patient education and counseling	42	1	15-24		Exclusion reason: Not along resettlement pathway;
Devins, G M; Beiser, M; Dion, R; Pelletier, L G; Edwards, R G	1997	Cross-cultural measurements of psychological well-being: the psychometric equivalence of Cantonese, Vietnamese, and Laotian translations of the Affect Balance Scale.	American journal of public health	87	5	794-9		Exclusion reason: Not along resettlement pathway;
Dietrich, Hans; Al Ali, Radwan; Tagay, Sefik; Hebebrand, Johannes; Reissner, Volker	2019	Screening for posttraumatic stress disorder in young adult refugees from Syria and Iraq.	Comprehensive psychiatry	90	do9, 0372612	73-81	https://dx.doi.org/	Exclusion reason: Ineligible study design;
Dixius, Andrea; Mohler, Eva	2017	A new therapy concept validating the special needs of refugeed children and adolescents: START-Stress-Traumasymptoms-Arousal-Regulation-Treatment.	Psychotherapie Forum	22	3	76-85	http://dx.doi.org/1	Exclusion reason: Full text unavailable;
Doolan, Emma L; Bryant, Richard A; Liddell, Belinda J; Nickerson, Angela	2017	The conceptualization of emotion regulation difficulties, and its association with posttraumatic stress symptoms in traumatized refugees.	Journal of anxiety disorders	50	co1, 8710131	7-14	https://dx.doi.org/	Exclusion reason: Not along resettlement pathway;
Elklit, Ask; Norregard, Jette; Tibor, Birgitte	1997	Trauma among young Bosnian refugees in Denmark.	Psykologisk Paedagogisk Radgivning	34	1	3-18		Exclusion reason: Full text unavailable;
Elklit, Ask; Ostergard Kjaer, Kamilla; Lasgaard, Mathias; Palic, Sabina	2012	Social support, coping and posttraumatic stress symptoms in young refugees.	Torture : quarterly journal on rehabilitation of torture victims and prevention of torture	22	1	11-23		Exclusion reason: Ineligible study design;
Ellis, B Heidi; Lhewa, Dechen; Charney, Meredith; Cabral, Howard	2006	Screening for PTSD among Somali adolescent refugees: psychometric properties of the UCLA PTSD Index.	Journal of traumatic stress	19	4	547-51		Exclusion reason: Not along resettlement pathway;
Ephraim, David	2002	Rorschach trauma assessment of survivors of torture and state violence.	Rorschachiana	25	1	58-76	http://dx.doi.org/1	Exclusion reason: Not a screening program;
Euteneuer, Frank; Schafer, Sarina J	2018	Brief Report: Subjective Social Mobility and Depressive Symptoms in Syrian Refugees to Germany.	Journal of immigrant and minority health	20	6	1533-1536	https://dx.doi.org/	Exclusion reason: Not a screening program;
Eytan, Ariel; Durieux-Paillard, Sophie; Whitaker-Clinch, Barbara; Loutan, Louis; Bovier, Patrick A	2007	Transcultural validity of a structured diagnostic interview to screen for major depression and posttraumatic stress disorder among refugees.	The Journal of nervous and mental disease	195	9	723-8		Exclusion reason: Duplicate;
Fawzi, M C; Pham, T; Lin, L; Nguyen, T V; Ngo, D; Murphy, E; Mollica, R F	1997	The validity of posttraumatic stress disorder among Vietnamese refugees.	Journal of traumatic stress	10	1	101-8		Exclusion reason: Not a screening program;

Authors	Published Year	Title	Journal	Volume	Issue	Pages	DOI	Notes
Fellmeth, Gracia; Plugge, Emma; Fazel, Mina; Charunwattana, Prakaykaew; Nosten, Francois; Fitzpatrick, Raymond; Simpson, Julie A; McGready, Rose	2018	Validation of the Refugee Health Screener-15 for the assessment of perinatal depression among Karen and Burmese women on the Thai-Myanmar border.	PloS one	13	5	e0197403	https://dx.doi.org/	Exclusion reason: Population is not refugee/asylum seeker;
Fox, Samara D; Griffin, Randi H; Pachankis, John E	2020	Minority stress, social integration, and the mental health needs of LGBTQ asylum seekers in North America.	Social science & medicine (1982)	246	ut9, 8303205	112727	https://dx.doi.org/	Exclusion reason: Not along resettlement pathway;
Francis J.; Mutch R.C.; Rutherford D. M.; Cherian S.	2012	Universal paediatric refugee health screening	Journal of Paediatrics and Child Health	48	11	1048-1049	http://dx.doi.org/1	Exclusion reason: Not a screening program;
Gabel U.; Ruf M.; Schauer M.; Odenwald M.; Neuner F.	2006	Prevalence of posttraumatic stress disorder among asylum seekers in Germany and its detection in the application process for asylum	Zeitschrift fur Klinische Psychologie und Psychotherapie	35	1	12-20	http://dx.doi.org/1	Exclusion reason: Full text unavailable;
Gadeberg, Anne Kristine; Norredam, Marie	2016	Urgent need for validated trauma and mental health screening tools for refugee children and youth.	European child & adolescent psychiatry	25	8	929-31	https://dx.doi.org/	Exclusion reason: Ineligible study design;
Gandham S.; Gunasekera H.; Isaacs D.; Maycock A.; Britton P.N.	2017	High prevalence of symptoms of post-traumatic stress in children of refugee and asylum seeker backgrounds	Journal of Paediatrics and Child Health	53	Supplement 3	16	http://dx.doi.org/1	Exclusion reason: Full text unavailable;
Georgiadou, Ekaterini; Morawa, Eva; Erim, Yesim	2017	High Manifestations of Mental Distress in Arabic Asylum Seekers Accommodated in Collective Centers for Refugees in Germany.	International journal of environmental research and public health	14	6		https://dx.doi.org/	Exclusion reason: Not a screening program;
Gerritsen, Annette A M; Bramsen, Inge; Deville, Walter; van Willigen, Loes H M; Hovens, Johannes E; van der Ploeg, Henk M	2006	Physical and mental health of Afghan, Iranian and Somali asylum seekers and refugees living in the Netherlands.	Social psychiatry and psychiatric epidemiology	41	1	18-26		Exclusion reason: Not a screening program;
Gerritsen, Annette A M; Bramsen, Inge; Deville, Walter; van Willigen, Loes H M; Hovens, Johannes E; van der Ploeg, Henk M	2004	Health and health care utilisation among asylum seekers and refugees in the Netherlands: design of a study.	BMC public health	4	100968562	7		Exclusion reason: Ineligible study design;
Getnet, Berhanie; Alem, Atalay	2019	Validity of the Center for Epidemiologic Studies Depression Scale (CES-D) in Eritrean refugees living in Ethiopia.	BMJ open	9	5	e026129	https://dx.doi.org/	Exclusion reason: Not along resettlement pathway;
Ghazinour, Mehdi; Richter, Jorg; Eisemann, Martin	2004	Quality of life among Iranian refugees resettled in Sweden.	Journal of immigrant health	6	2	71-81		Exclusion reason: Not along resettlement pathway;
Goodman, Laura F; Jensen, Guy W; Galante, Joseph M; Farmer, Diana L; Tache, Stephanie	2018	A cross-sectional investigation of the health needs of asylum seekers in a refugee clinic in Germany.	BMC family practice	19	1	64	https://dx.doi.org/	Exclusion reason: Ineligible study design;
Goodwin, Robin; Takahashi, Masahito; Sun, Shaojing; Ben-Ezra, Menachem	2015	Psychological distress among tsunami refugees from the Great East Japan earthquake.	BJPsych open	1	1	92-97		Exclusion reason: Ineligible study design;
Gottlieb, Nora; Puschmann, Conny; Stenzinger, Fabian; Koelber, Julia; Rasch, Laurette; Koppelow, Martha; Al Munjid, Razan	2020	Health and Healthcare Utilization among Asylum-Seekers from Berlin's LGBTIQ Shelter: Preliminary Results of a Survey.	International journal of environmental research and public health	17	12		https://dx.doi.org/	Exclusion reason: Ineligible study design;
Gottvall, Maria; Sjolund, Sara; Arwidson, Charlotta; Saboonchi, Fredrik	2019	Health-related quality of life among syrian refugees resettled in sweden.	Quality of Life Research: An International Journal of Quality of Life Aspects of Treatment, Care & Rehabilitation			No-Specified	http://dx.doi.org/1	Exclusion reason: Ineligible study design;
Green, Aliza S; Ruchman, Samuel G; Katz, Craig L; Singer, Elizabeth K	2020	Piloting forensic tele-mental health evaluations of asylum seekers.	Psychiatry research	291	qc4, 7911385	113256	https://dx.doi.org/	Exclusion reason: Ineligible study design;
Gulden, Ashley; Westermeyer, Joseph; Lien, Rebecca; Spring, Marline; Johnson, David; Butcher, James; Jaranson, James	2010	HADStress screen for posttraumatic stress: replication in ethiopian refugees.	The Journal of nervous and mental disease	198	10	762-7	https://dx.doi.org/	Exclusion reason: Not along resettlement pathway;
Haith-Cooper, Melanie; Waskett, Catherine; Montague, Jane; Horne, Maria	2018	Exercise and physical activity in asylum seekers in Northern England; using the theoretical domains framework to identify barriers and facilitators.	BMC public health	18	1	762	https://dx.doi.org/	Exclusion reason: No mental health screening;
Hamdan-Mansour, Ayman M; Abdel Razeq, Nadin M; AbdulHaq, Bayan; Arabiat, Diana; Khalil, Amani A	2017	Displaced Syrian children's reported physical and mental wellbeing.	Child and adolescent mental health	22	4	186-193	https://dx.doi.org/	Exclusion reason: Ineligible study design;
Hamrah, Mohammad Shoaib; Hoang, Ha; Mond, Jonathan; Pahlavanzade, Bagher; Charkazi, Abdurrahman; Auckland, Stuart	2020	The prevalence and correlates of symptoms of post-traumatic stress disorder (PTSD) among resettled Afghan refugees in a regional area of Australia.	Journal of mental health (Abingdon, England)		9212352	1-7	https://dx.doi.org/	Exclusion reason: Not along resettlement pathway;
Hebebrand, Johannes; Anagnostopoulos, Dimitris; Eliez, Stephan; Linse, Henk; Pejovic-Milovancevic, Milica; Klasen, Henrikje	2016	A first assessment of the needs of young refugees arriving in Europe: what mental health professionals need to know.	European child & adolescent psychiatry	25	1	1-6	https://dx.doi.org/	Exclusion reason: Ineligible study design;
Hecker, Tobias; Huber, Stephanie; Maier, Thomas; Maercker, Andreas	2018	Differential Associations Among PTSD and Complex PTSD Symptoms and Traumatic Experiences and Postmigration Difficulties in a Culturally Diverse Refugee Sample.	Journal of traumatic stress	31	6	795-804	https://dx.doi.org/	Exclusion reason: Not along resettlement pathway;

Authors	Published Year	Title	Journal	Volume	Issue	Pages	DOI	Notes
Heeren, Martina; Wittmann, Lutz; Ehlert, Ulrike; Schnyder, Ulrich; Maier, Thomas; Muller, Julia	2014	Psychopathology and resident status - comparing asylum seekers, refugees, illegal migrants, labor migrants, and residents.	Comprehensive psychiatry	55	4	818-25	https://dx.doi.org/	Exclusion reason: Not along resettlement pathway;
Hengst, Sophie M C; Smid, Geert E; Laban, Cornelis J	2018	The Effects of Traumatic and Multiple Loss on Psychopathology, Disability, and Quality of Life in Iraqi Asylum Seekers in the Netherlands.	The Journal of nervous and mental disease	206	1	52-60	https://dx.doi.org/	Exclusion reason: Not along resettlement pathway;
Hermansson A.-C.; Timpka T.	1999	How do you feel?': A self-rating scale for measuring well-being in refugees	Transcultural Psychiatry	36	3	317-328	http://dx.doi.org/1	Exclusion reason: Not along resettlement pathway;
Hermansson, Ann-Charlotte; Timpka, Toomas; Thyberg, Mikael	2003	The long-term impact of torture on the mental health of war-wounded refugees: findings and implications for nursing programmes.	Scandinavian journal of caring sciences	17	4	317-24		Exclusion reason: Ineligible study design;
Hewlett, Montana; Merry, Lisa; Mishra, Anit; Islam, Risatul; Wali, Raz Mohammad; Gagnon, Anita	2015	Alcohol use among Bhutanese refugees in Nepal.	International Journal of Migration, Health & Social Care	11	3	158-168	10.1108/IJMHSC	Exclusion reason: Not along resettlement pathway;
Hinton D.E.; Sinclair S.J.; Chung R.C.-Y.; Pollack M.H.	2007	The SF-36 among Cambodian and Vietnamese refugees: An examination of psychometric properties	Journal of Psychopathology and Behavioral Assessment	29	1	39-46	http://dx.doi.org/1	Exclusion reason: Not along resettlement pathway;
Hinton, Devon E; Chhean, Dara; Pich, Vuth; Pollack, M H; Orr, Scott P; Pitman, Roger K	2006	Assessment of posttraumatic stress disorder in Cambodian refugees using the Clinician-Administered PTSD Scale: psychometric properties and symptom severity.	Journal of traumatic stress	19	3	405-9		Exclusion reason: Not along resettlement pathway;
Hinton, Devon E; Kredlow, M Alexandra; Pich, Vuth; Bui, Eric; Hofmann, Stefan G	2013	The relationship of PTSD to key somatic complaints and cultural syndromes among Cambodian refugees attending a psychiatric clinic: the Cambodian Somatic Symptom and Syndrome Inventory (CSSI).	Transcultural psychiatry	50	3	347-70	https://dx.doi.org/	Exclusion reason: Not a screening program;
Hinton, Devon E; Pich, Vuth; Safren, Steven A; Pollack, Mark H; McNally, Richard J	2005	Anxiety sensitivity in traumatized Cambodian refugees: a discriminant function and factor analytic investigation.	Behaviour research and therapy	43	12	1631-43		Exclusion reason: Not along resettlement pathway;
Hinton, Devon E; Reis, Ria; de Jong, Joop	2015	The "Thinking a Lot" Idiom of Distress and PTSD: An Examination of Their Relationship among Traumatized Cambodian Refugees Using the "Thinking a Lot" Questionnaire.	Medical anthropology quarterly	29	3	357-80	https://dx.doi.org/	Exclusion reason: Not along resettlement pathway;
Hinton, W L; Tiet, Q; Tran, C G; Chesney, M	1997	Predictors of depression among refugees from Vietnam: a longitudinal study of new arrivals.	The Journal of nervous and mental disease	185	1	39-45		Exclusion reason: Ineligible study design;
Hocking, Debbie C; Kennedy, Gerard A; Sundram, Suresh	2015	Mental disorders in asylum seekers: the role of the refugee determination process and employment.	The Journal of nervous and mental disease	203	1	28-32	https://dx.doi.org/	Exclusion reason: Ineligible study design;
Hoffmann, Christopher; McFarland, Bentson H; Kinzie, J David; Bresler, Larissa; Rakhlin, Dmitriy; Wolf, Solomon; Kovas, Anne E	2005	Psychometric properties of a Russian version of the SF-12 Health Survey in a refugee population.	Comprehensive psychiatry	46	5	390-7		Exclusion reason: Not along resettlement pathway;
Hollifield, Michael; Eckert, Valorie; Warner, Teddy D; Jenkins, Janis; Krakow, Barry; Ruiz, James; Westermeyer, Joseph	2005	Development of an inventory for measuring war-related events in refugees.	Comprehensive psychiatry	46	1	67-80		Exclusion reason: Not along resettlement pathway;
Hollifield, Michael; Warner, Teddy D; Krakow, Barry; Jenkins, Janis; Westermeyer, Joseph	2009	The range of symptoms in refugees of war: the New Mexico Refugee Symptom Checklist-121.	The Journal of nervous and mental disease	197	2	117-25	https://dx.doi.org/	Exclusion reason: Screening program administered post 12months of settlement;
Hossain M.; Pearson R.; McAlpine A.; Bacchus L.; Muuo S.W.; Muthuri S.K.; Spangaro J.; Kuper H.; Franchi G.; Cordero R.P.; Cornish-Spencer S.; Hess T.; Bangha M.; Izugbara C.	2020	Disability, violence, and mental health among Somali refugee women in a humanitarian setting	Global Mental Health	7	(Hossain, McAlpi	e30	http://dx.doi.org/1	Exclusion reason: Ineligible study design;
Hussain, Dilwar; Bhushan, Braj	2009	Development and validation of the Refugee Trauma Experience Inventory.	Psychological Trauma: Theory, Research, Practice, and Policy	1	2	107-117	http://dx.doi.org/1	Exclusion reason: Screening program administered post 12months of settlement;
Hvidtfeldt, Camilla; Petersen, Jorgen Holm; Norredam, Marie	2020	Prolonged periods of waiting for an asylum decision and the risk of psychiatric diagnoses: a 22-year longitudinal cohort study from Denmark.	International journal of epidemiology	49	2	400-409	https://dx.doi.org/	Exclusion reason: Not a screening program;
Hyland, P; Ceannt, R; Daccache, F; Abou Daher, R; Sleiman, J; Gilmore, B; Byrne, S; Shevlin, M; Murphy, J; Vallieres, F	2018	Are posttraumatic stress disorder (PTSD) and complex-PTSD distinguishable within a treatment-seeking sample of Syrian refugees living in Lebanon?.	Global mental health (Cambridge, England)	5	101659641	e14	https://dx.doi.org/	Exclusion reason: Not along resettlement pathway;
Ing, Harriet; Fellmeth, Gracia; White, Jitrachote; Stein, Alan; Simpson, Julie A; McGready, Rose	2017	Validation of the Edinburgh Postnatal Depression Scale (EPDS) on the Thai-Myanmar border.	Tropical doctor	47	4	339-347	https://dx.doi.org/	Exclusion reason: Not along resettlement pathway;
Islam L.; Lamberti R.; Bardino M.; Grijuela B.; Matarazzo E.; Rizzo Y.; Ranieri R.	2018	Trauma, migration and mental health in a sample of asylum-seeking women in Italy	European Psychiatry	48	Supplement 1	S453	http://dx.doi.org/1	Exclusion reason: Full text unavailable;
Jakobsen, Marianne; Demott, Melinda A M; Heir, Trond	2014	Prevalence of psychiatric disorders among unaccompanied asylum-seeking adolescents in norway.	Clinical practice and epidemiology in mental health : CP & EMH	10	101245735	53-8	https://dx.doi.org/	Exclusion reason: Ineligible study design;

Authors	Published Year	Title	Journal	Volume	Issue	Pages	DOI	Notes
Javanbakht, Arash; Grasser, Lana Ruvolo; Kim, Soyeong; Arfken, Cynthia L; Nugent, Nicole	2020	Perceived health, adversity, and posttraumatic stress disorder in Syrian and Iraqi refugees.	The International journal of social psychiatry		gt5, 0374726	2076402097827	https://dx.doi.org/	Exclusion reason: Ineligible study design;
Jensen, Natasja Koitzsch; Norredam, Marie; Priebe, Stefan; Krasnik, Allan	2013	How do general practitioners experience providing care to refugees with mental health problems? A qualitative study from Denmark.	BMC family practice	14	100967792	17	https://dx.doi.org/	Exclusion reason: Population is not refugee/asylum seeker;
Jensen, Tine K; Fjermestad, Krister W; Granly, Lene; Wilhelmsen, Nicolai H	2015	Stressful life experiences and mental health problems among unaccompanied asylum-seeking children.	Clinical child psychology and psychiatry	20	1	106-16	https://dx.doi.org/	Exclusion reason: Not a screening program;
Jensen, Tine K; Skar, Ane-Marthe Solheim; Andersson, Elin Sofia; Birkeland, Marianne Skogbrott	2019	Long-term mental health in unaccompanied refugee minors: pre- and post-flight predictors.	European child & adolescent psychiatry	28	12	1671-1682	https://dx.doi.org/	Exclusion reason: Ineligible study design;
Jeon, Woo-Teak; Yu, Shi-Eun; Cho, Young-A; Eom, Jin-Sup	2008	Traumatic experiences and mental health of north korean refugees in South Korea.	Psychiatry investigation	5	4	213-20	https://dx.doi.org/	Exclusion reason: Not a screening program;
Jeremie B.; Anne-Emmanuelle A.; Martine M.; Florence F.; Javier S.; Alejandro R.-U.; Bernard H.; Mario G.; Patrick B.	2017	Assessing the plight of young unaccompanied refugees	Swiss Medical Weekly	147	47-48	w14547	http://dx.doi.org/1	Exclusion reason: Not a screening program;
Joshua P.; Koh A.; Woodland L.; Zwi K.	2010	Refugee child health screening in the illawarra: A comprehensive population screening program	Journal of Paediatrics and Child Health	46	SUPPL. 2	8	http://dx.doi.org/1	Exclusion reason: Full text unavailable;
Kaiser, B. N; Ticao, C; Anoje, C; Minto, J; Boglosa, J; Kohrt, B. A	2019	Adapting culturally appropriate mental health screening tools for use among conflict-affected and other vulnerable adolescents in Nigeria.	Global Mental Health	6	Health Psychology & Medicine [33		http://dx.doi.org/1	Exclusion reason: Population is not refugee/asylum seeker;
Kaur, Kushilpal; Sulaiman, Ahmad Hatim; Yoon, Chee Kok; Hashim, Aili Hanim; Kaur, Manveen; Hui, Koh Ong; Sabki, Zuraida Ahmad; Francis, Benedict; Singh, Sarbhan; Gill, Jesjeet Singh	2020	Elucidating Mental Health Disorders among Rohingya Refugees: A Malaysian Perspective.	International journal of environmental research and public health	17	18		https://dx.doi.org/	Exclusion reason: Not along resettlement pathway;
Kaya, Edip; Kilic, Cengiz; Karadag Caman, Ozge; Uner, Sarp	2019	Posttraumatic Stress and Depression Among Syrian Refugees Living in Turkey: Findings From an Urban Sample.	The Journal of nervous and mental disease	207	12	995-1000	https://dx.doi.org/	Exclusion reason: Not along resettlement pathway;
Keller, Allen S; Rosenfeld, Barry; Trinh-Shevrin, Chau; Meserve, Chris; Sachs, Emily; Leviss, Jonathan A; Singer, Elizabeth; Smith, Hawthorne; Wilkinson, John; Kim, Glen; Allden, Kathleen; Ford, Douglas	2003	Mental health of detained asylum seekers.	Lancet (London, England)	362	9397	1721-3		Exclusion reason: Not along resettlement pathway;
Kennedy, J; Seymour, D J; Hummel, B J	1999	A comprehensive refugee health screening program.	Public health reports (Washington, D.C. : 1974)	114	5	469-77		Exclusion reason: Ineligible study design;
Kiat, Naama; Youngmann, Rafael; Lurie, Ido	2017	The emotional distress of asylum seekers in Israel and the characteristics of those seeking psychiatric versus medical help.	Transcultural psychiatry	54	5-6	575-594	https://dx.doi.org/	Exclusion reason: Not a screening program;
Kim, Seong-Hyeon; Goodman, Grace M; Toruno, Joseph A; Sherry, Alissa R; Kim, Hee Kyung	2015	The cross-cultural validity of the MMPI-2-RF Higher-Order Scales in a sample of North Korean female refugees.	Assessment	22	5	640-649	http://dx.doi.org/1	Exclusion reason: Not a screening program;
Kira, Ibrahim A; Ramaswamy, Vidya; Lewandowski, Linda; Mohanesh, Jamal; Abdul-Khalek, Husam	2015	Psychometric assessment of the Arabic version of the Internalized Stigma of Mental Illness (ISMI) measure in a refugee population.	Transcultural psychiatry	52	5	636-58	https://dx.doi.org/	Exclusion reason: Not along resettlement pathway;
Kira, Ibrahim A; Templin, Thomas; Lewandowski, Linda; Ashby, Jeffery S; Oladele, Alwande; Odenat, Lydia	2012	Cumulative Trauma Disorder scale (CTD): Two studies.	Psychology	3	9	643-656	http://dx.doi.org/1	Exclusion reason: Not along resettlement pathway;
Kiselev, Nikolai; Pfaltz, Monique; Schick, Matthis; Bird, Martha; Pernille, Hansen; Sijbrandij, Marit; de Graaff, Anne M; Schnyder, Ulrich; Morina, Naser	2020	Problems faced by Syrian refugees and asylum seekers in Switzerland.	Swiss medical weekly	150	d10, 100970884	w20381	https://dx.doi.org/	Exclusion reason: Ineligible study design;
Kliem, Soren; Mosle, Thomas; Klatt, Thimna; Fleischer, Stephanie; Kudlacek, Dominic; Kroger, Christoph; Brahler, Elmar; Beutel, Manfred E; Wiltink, Jorg	2016	[Psychometric Evaluation of an Arabic Version of the PHQ-4 Based on a Representative Survey of Syrian Refugees].	Psychotherapie, Psychosomatik, medizinische Psychologie	66	9-10	385-392		Exclusion reason: Not a screening program;
Kroger, Christoph; Frantz, Inga; Friel, Pauline; Heinrichs, Nina	2016	Posttraumatic stress and depressive symptoms amongst asylum seekers: Screening in a State Refugee Reception Center.	PPmP: Psychotherapie Psychosomatik Medizinische Psychologie	66	9-10	377-384	http://dx.doi.org/1	Exclusion reason: Duplicate;
Kroger, Christoph; Frantz, Inga; Friel, Pauline; Heinrichs, Nina	2016	[Posttraumatic Stress and Depressive Symptoms amongst Asylum Seekers].	Psychotherapie, Psychosomatik, medizinische Psychologie	66	9-10	377-384		Exclusion reason: Duplicate;
Kronick, Rachel	2018	Mental Health of Refugees and Asylum Seekers: Assessment and Intervention.	Canadian journal of psychiatry. Revue canadienne de psychiatrie	63	5	290-296	https://dx.doi.org/	Exclusion reason: Ineligible study design;

Authors	Published Year	Title	Journal	Volume	Issue	Pages	DOI	Notes
Laban, Cornelis J; Gernaat, Hajo B P E; Komproe, Ivan H; van der Tweel, Ingeborg; De Jong, Joop T V M	2005	Postmigration living problems and common psychiatric disorders in Iraqi asylum seekers in the Netherlands.	The Journal of nervous and mental disease	193	12	825-32		Exclusion reason: Not a screening program;
Laugharne J.	2012	Investigating the mental health of refugees recently arrived in western Australia	European Psychiatry	27	SUPPL. 1		http://dx.doi.org/1	Exclusion reason: conference poster/ abstract;
Laugharne J.; Thambiran A.; Lillee A.	2011	Investigating the mental health of adult refugees recently arrived in Western Australia	Australian and New Zealand Journal of Psychiatry	45	O1	A41		Exclusion reason: conference poster/ abstract;
Laukamp A.; Prufer-Kramer L.; Fischer F.; Kramer A.	2019	Health of Syrian unaccompanied asylum seeking adolescents (UASA) at first medical examination in Germany in comparison to UASA from other world regions	BMC International Health and Human Rights	19	1	5	http://dx.doi.org/1	Exclusion reason: Ineligible study design;
Laws, A; Patsalides, B	1997	Medical and psychological examination of women seeking asylum: documentation of human rights abuses.	Journal of the American Medical Women's Association (1972)	52	4	185-7		Exclusion reason: Full text unavailable;
Leiler, Anna; Bjärtå, Anna; Ekdahl, Johanna; Wasteson, Elisabet	2019	Mental health and quality of life among asylum seekers and refugees living in refugee housing facilities in Sweden.	Social Psychiatry & Psychiatric Epidemiology	54	5	543-551	10.1007/s00127-	Exclusion reason: Ineligible study design;
Leiler, Anna; Hollifield, Michael; Wasteson, Elisabet; Bjarta, Anna	2019	Suicidal Ideation and Severity of Distress among Refugees Residing in Asylum Accommodations in Sweden.	International journal of environmental research and public health	16	15		https://dx.doi.org/	Exclusion reason: Ineligible study design;
LeMaster JW, Broadbridge CL, Lumley MA, Arnetz JE, Arfken C, Fetters MD, Jamil H, Pole N, Arnetz BB	2017	Acculturation and Post-Migration Psychological Symptoms Among Iraqi Refugees: a Path Analysis		Date of Publication: March 02				Exclusion reason: Duplicate;
Lepper, Leigh E Tenkku; Karamehic-Muratovic, Ajlina; Salas, Joanne; Pollard, C Alec; Karahodzic, Edina; Asher, Jaron	2017	Mental Health Screening in a Bosnian Refugee Population Using the Primary Care Behavioral Health Screener-Bosnian Translation.	Journal of clinical psychology in medical settings	24	2	152-162	https://dx.doi.org/	Exclusion reason: Not along resettlement pathway;
Lies J.; Drummond S.	2017	Prevalence study of sleep disturbance, mental health, and psychosocial concerns among asylum seekers and refugees	Journal of Sleep Research	26	Supplement 1	45-46	http://dx.doi.org/1	Exclusion reason: Full text unavailable;
Llosa, Augusto E; Van Ommeren, Mark; Kolappa, Kavitha; Ghantous, Zeina; Souza, Renato; Bastin, Pierre; Slavuckij, Andrej; Grais, Rebecca F	2017	A two-phase approach for the identification of refugees with priority need for mental health care in Lebanon: a validation study.	BMC psychiatry	17	1	28	https://dx.doi.org/	Exclusion reason: Not along resettlement pathway;
Logie, Carmen H; Okumu, Moses; Mwima, Simon; Hakiza, Robert; Chemutai, Doreen; Kyambadde, Peter	2020	Contextual factors associated with depression among urban refugee and displaced youth in Kampala, Uganda: findings from a cross-sectional study.	Conflict and health	14	101286573	45	https://dx.doi.org/	Exclusion reason: Not along resettlement pathway;
Lopes Cardozo, Barbara; Talley, Leisel; Burton, Ann; Crawford, Carol	2004	Karenni refugees living in Thai-Burmese border camps: traumatic experiences, mental health outcomes, and social functioning.	Social science & medicine (1982)	58	12	2637-44		Exclusion reason: Not along resettlement pathway;
Loutan, L; Bierens de Haan, D; Subilia, L	1997	[The health of asylum seekers: from communicable disease screening to post-traumatic disorders].	Bulletin de la Societe de pathologie exotique (1990)	90	4	233-7		Exclusion reason: Full text unavailable;
Lupone C.D.; Cronkright P.	2017	Emotional distress screening tool as a predictor for medical utilization and disability: A retrospective analysis of refugees resettling in Syracuse, NY	Annals of Global Health	83	1	172		Exclusion reason: Full text unavailable;
M'zah, Skander; Lopes Cardozo, Barbara; Evans, Dabney P	2019	Mental Health Status and Service Assessment for Adult Syrian Refugees Resettled in Metropolitan Atlanta: A Cross-Sectional Survey.	Journal of immigrant and minority health	21	5	1019-1025	https://dx.doi.org/	Exclusion reason: Not along resettlement pathway;
Magdalini P.; Narring F.; Chamay-Weber C.	2018	Health care needs for unaccompanied asylum-seeking adolescents: A retrospec-tive study from an adolescent health unit in switzerland	Journal of Adolescent Health	62	2 Supplement 1	S108-S109		Exclusion reason: Ineligible study design;
Maldari, Toni; Elsley, Natasha; Rahim, Razlyn Abdul	2019	The health status of newly arrived Syrian refugees at the Refugee Health Service, South Australia, 2016.	Australian journal of general practice	48	7	480-486	https://dx.doi.org/	Exclusion reason: Not a screening program;
Malekzai, A S; Niazi, J M; Paige, S R; Hendricks, S E; Fitzpatrick, D; Leuschen, M P; Millimet, C R	1996	Modification of CAPS-1 for diagnosis of PTSD in Afghan refugees.	Journal of traumatic stress	9	4	891-8		Exclusion reason: Not along resettlement pathway;
Malm, Andreas; Tinghog, Petter; Narusyte, Jurgita; Saboonchi, Fredrik	2020	The refugee post-migration stress scale (RPMS) - development and validation among refugees from Syria recently resettled in Sweden.	Conflict and health	14	101286573	2	https://dx.doi.org/	Exclusion reason: Not along resettlement pathway;
Marusic, Ana; Kozaric-Kovacic, Dragica; Folnegovic-Smalc, Vera; Ljubin, Tajana; et al	1995	Use of two PTSD scales in assessing posttraumatic stress disorder in refugees and displaced persons from Bosnia and Herzegovina and Croatia.	Psychologische Beitrage	37	1-2	209-214		Exclusion reason: Not along resettlement pathway;
Mazur V.; Chahraoui K.	2011	Psychopathology of asylum seekers in Slovak Republic. Case study of 40 asylum seekers	Annales Medico-Psychologiques	169	10	621-626	http://dx.doi.org/1	Exclusion reason: Ineligible study design;
Minhas, Ripudaman S; Graham, Hamish; Jegathesan, Thivia; Huber, Joelene; Young, Elizabeth; Barozzino, Tony	2017	Supporting the developmental health of refugee children and youth.	Paediatrics & child health	22	2	68-71	https://dx.doi.org/	Exclusion reason: Ineligible study design;

Authors	Published Year	Title	Journal	Volume	Issue	Pages	DOI	Notes
Mishori, Ranit; Aleinikoff, Shoshana; Davis, Dawn	2017	Primary Care for Refugees: Challenges and Opportunities.	American family physician	96	2	112-120		Exclusion reason: Ineligible study design;
Mogos, Mulubrhan F.; Beckstead, Jason W.; Evans, Mary E.; Kip, Kevin E.; Boothroyd, Roger A.	2019	Forward-backward translation and cross-cultural validation of the Center for Epidemiologic Studies Depression scale among Tigrigna-speaking Eritrean refugees.	International Journal of Migration, Health & Social Care	15	2	163-176	10.1108/IJMHSC	Exclusion reason: Not along resettlement pathway;
Mohler, Eva; Simons, Michael; Kolch, Michael; Herpertz-Dahlmann, Beate; Schulte-Markwort, Michael; Fegert, Jorg M	2015	[Diagnoses and treatment of (unaccompanied) refugee minors - a major challenge for child and adolescent psychiatry in Germany].	Zeitschrift fur Kinder- und Jugendpsychiatrie und Psychotherapie	43	6	381-3	https://dx.doi.org/	Exclusion reason: Full text unavailable;
Morville, Anne-Le; Erlandsson, Lena-Karin; Eklund, Mona; Danneskiold-Samsoe, Bente; Christensen, Robin; Amris, Kirstine	2014	Activity of daily living performance amongst Danish asylum seekers: a cross-sectional study.	Torture : quarterly journal on rehabilitation of torture victims and prevention of torture	24	1	49-64		Exclusion reason: Not a screening program;
Mulhall D.J.	2003	The psychological assessment of asylum seekers	Clinical Psychology		22	4-6		Exclusion reason: Full text unavailable;
Muller, Lauritz Rudolf Floribert; Buter, Karl Phillipp; Rosner, Rita; Unterhitzenberger, Johanna	2019	Mental health and associated stress factors in accompanied and unaccompanied refugee minors resettled in Germany: a cross-sectional study.	Child and adolescent psychiatry and mental health	13	101297974	8	https://dx.doi.org/	Exclusion reason: Not along resettlement pathway;
Muller, Matthias Johannes; Kamcili-Kubach, Suzan; Strassheim, Songul; Koch, Eckhardt	2012	Assessing stressors related to migration in patients with psychiatric disorders: Development and reliability of a standardized instrument (MIGSTR10).	European Journal of Psychological Assessment	28	4	262-269	http://dx.doi.org/1	Exclusion reason: Population is not refugee/asylum seeker;
Nallusamy, Vasumathi; Afgarshe, Mohamud; Shlosser, Heather	2016	Reliability and validity of Somali version of the PHQ-9 in primary care practice.	International journal of psychiatry in medicine	51	6	508-520	https://dx.doi.org/	Exclusion reason: Population is not refugee/asylum seeker;
Nesterko, Y; Jackle, D; Friedrich, M; Holzapfel, L; Glaesmer, H	2019	Prevalence of post-traumatic stress disorder, depression and somatisation in recently arrived refugees in Germany: an epidemiological study.	Epidemiology and psychiatric sciences	29	101561091	e40	https://dx.doi.org/	Exclusion reason: Ineligible study design;
Nesterko, Yuriy; Jackle, David; Friedrich, Michael; Holzapfel, Laura; Glaesmer, Heide	2020	Health care needs among recently arrived refugees in Germany: a cross-sectional, epidemiological study.	International journal of public health	65	6	811-821	https://dx.doi.org/	Exclusion reason: Not a screening program;
Nesterko, Yuriy; Jackle, David; Friedrich, Michael; Holzapfel, Laura; Glaesmer, Heide	2020	Factors predicting symptoms of somatization, depression, anxiety, post-traumatic stress disorder, self-rated mental and physical health among recently arrived refugees in Germany.	Conflict and health	14	101286573	44	https://dx.doi.org/	Exclusion reason: Ineligible study design;
Nickerson, Angela; Schick, Matthis; Schnyder, Ulrich; Bryant, Richard A; Morina, Naser	2017	Comorbidity of Posttraumatic Stress Disorder and Depression in Tortured, Treatment-Seeking Refugees.	Journal of traumatic stress	30	4	409-415	https://dx.doi.org/	Exclusion reason: Not along resettlement pathway;
Nielsen, Signe Smith; Norredam, Marie; Christensen, Karen Louise; Obel, Carsten; Krasnik, Allan	2007	[The mental health of asylum-seeking children in Denmark].	Ugeskrift for laeger	169	43	3660-5		Exclusion reason: Not along resettlement pathway;
Nijman, Ruud Gerard; Krone, Johanna; Mintegi, Santiago; Bidlingmaier, Christoph; Maconochie, Ian K.; Lyttle, Mark D.; von Both, Ulrich	2021	Emergency care provided to refugee children in Europe: RefuNET: a cross-sectional survey study.	Emergency Medicine Journal	38	1	5-13	10.1136/emerme	Exclusion reason: Not a screening program;
Nose, Michela; Tarsitani, Lorenzo; Tedeschi, Federico; Lotito, Claudia; Massetti, Paola; Purgato, Marianna; Roselli, Valentina; Todini, Liliana; Turrini, Giulia; Barbui, Corrado	2020	Association of traumatic events with levels of psychological distress and depressive symptoms in male asylum seekers and refugees resettled in Italy.	BMC psychiatry	20	1	576	https://dx.doi.org/	Exclusion reason: Not a screening program;
Nose, Michela; Turrini, Giulia; Imoli, Maria; Ballette, Francesca; Ostuzzi, Giovanni; Cucchi, Francesca; Padoan, Chiara; Ruggeri, Mirella; Barbui, Corrado	2018	Prevalence and Correlates of Psychological Distress and Psychiatric Disorders in Asylum Seekers and Refugees Resettled in an Italian Catchment Area.	Journal of immigrant and minority health	20	2	263-270	https://dx.doi.org/	Exclusion reason: Not along resettlement pathway;
O'Laughlin K.N.; Xu A.; Ashaba S.; Khidiir H.; Parker R.; Faustin Z.; Muwonge T.; Tsai A.; Bassett I.; Strehlow M.	2019	Assessing the burden of mental illness among refugees in uganda	Academic Emergency Medicine	26	Supplement 1	S68	http://dx.doi.org/1	Exclusion reason: Not along resettlement pathway;
Oda, Anna; Tuck, Andrew; Agic, Branka; Hynie, Michaela; Roche, Brenda; McKenzie, Kwame	2017	Health care needs and use of health care services among newly arrived Syrian refugees: a cross-sectional study.	CMAJ open	5	2	E354-E358	https://dx.doi.org/	Exclusion reason: Ineligible study design;
Palic, Sabina; Kappel, Michelle Lind; Nielsen, Monica Stougaard; Carlsson, Jessica; Bech, Per	2014	Comparison of psychiatric disability on the health of nation outcome scales (HoNOS) in resettled traumatized refugee outpatients and Danish inpatients.	BMC psychiatry	14	100968559	330	https://dx.doi.org/	Exclusion reason: Not along resettlement pathway;
Patseadou M.; Liso-Navarro A.; Djapo S.; Narring F.	2018	Mental health care needs of unaccompanied asylum-seeking minors resettled in the canton of geneva: The importance of networking	Swiss Medical Weekly	147	Supplement 228	53S		Exclusion reason: conference poster/ abstract;
Peltzer, K	1999	Trauma and mental health problems of Sudanese refugees in Uganda.	The Central African journal of medicine	45	5	110-4		Exclusion reason: Not along resettlement pathway;

Authors	Published Year	Title	Journal	Volume	Issue	Pages	DOI	Notes
Pfeiffer, Elisa; Sukale, Thorsten; Muller, Lauritz Rudolf Floribert; Plener, Paul Lukas; Rosner, Rita; Fegert, Joerg Michael; Sachser, Cedric; Unterhitzenberger, Johanna	2019	The symptom representation of posttraumatic stress disorder in a sample of unaccompanied and accompanied refugee minors in Germany: a network analysis.	European journal of psychotraumatology	10	1	1675990	https://dx.doi.org/	Exclusion reason: Ineligible study design;
Polcher, Kelly; Calloway, Susan	2016	Addressing the Need for Mental Health Screening of Newly Resettled Refugees: A Pilot Project.	Journal of primary care & community health	7	3	199-203	https://dx.doi.org/	Exclusion reason: Duplicate;
Poole G.E.; Galpin G.	2011	Prevalence of victims of torture in the health screening of quota refugees in New Zealand during 2007-2008 and implications for follow-up care	New Zealand Medical Journal	124	1338	18-24		Exclusion reason: Full text unavailable;
Poole, Danielle N; Liao, Shirley; Larson, Elysia; Hedt-Gauthier, Bethany; Raymond, Nathaniel A; Barnighausen, Till; Smith Fawzi, Mary C	2020	Sequential screening for depression in humanitarian emergencies: a validation study of the Patient Health Questionnaire among Syrian refugees.	Annals of general psychiatry	19	101236515	5	https://dx.doi.org/	Exclusion reason: Not along resettlement pathway;
Puric, Danka; Vukcevic Markovic, Masa	2019	Development and validation of the Stressful Experiences in Transit Questionnaire (SET-Q) and its Short Form (SET-SF).	European journal of psychotraumatology	10	1	1611091	https://dx.doi.org/	Exclusion reason: Ineligible study design;
Ramos, Katherine; Jones, Martinque K; Shellman, Alison B; Dao, Tam K; Szeto, Kim	2016	Reliability and Validity of the Vietnamese Depression Interview (VDI).	Journal of immigrant and minority health	18	4	799-809	https://dx.doi.org/	Exclusion reason: Population is not refugee/asylum seeker;
Renner, Walter; Salem, Ingrid	2009	Post-traumatic stress in asylum seekers and refugees from Chechnya, Afghanistan, and West Africa: gender differences in symptomatology and coping.	The International journal of social psychiatry	55	2	99-108	https://dx.doi.org/	Exclusion reason: Ineligible study design;
Rodolico, Alessandro; Vaccino, Noemi; Riso, Maria C; Concerto, Carmen; Aguglia, Eugenio; Signorelli, Maria S	2020	Prevalence of Post-Traumatic Stress Disorder Among Asylum Seekers in Italy: A Population-Based Survey in Sicily.	Journal of immigrant and minority health	22	3	634-638	https://dx.doi.org/	Exclusion reason: Not along resettlement pathway;
Rothe, Eugenio M; Lewis, John; Castillo-Matos, Hector; Martinez, Orestes; Busquets, Ruben; Martinez, Igna	2002	Posttraumatic stress disorder among Cuban children and adolescents after release from a refugee camp.	Psychiatric services (Washington, D.C.)	53	8	970-6		Exclusion reason: Not a screening program;
Salt, Rebekah J; Costantino, Margaret E; Dotson, Emma L; Paper, Bruce M	2017	"You Are Not Alone" Strategies for Addressing Mental Health and Health Promotion with a Refugee Women's Sewing Group.	Issues in mental health nursing	38	4	337-343	https://dx.doi.org/	Exclusion reason: Not a screening program;
Schnyder, Ulrich; Muller, Julia; Morina, Naser; Schick, Matthis; Bryant, Richard A; Nickerson, Angela	2015	A Comparison of DSM-5 and DSM-IV Diagnostic Criteria for Posttraumatic Stress Disorder in Traumatized Refugees.	Journal of traumatic stress	28	4	267-74	https://dx.doi.org/	Exclusion reason: Not a screening program;
Schubert, Carla C; Punamaki, Raija-Leena	2011	Mental health among torture survivors: cultural background, refugee status and gender.	Nordic journal of psychiatry	65	3	175-82	https://dx.doi.org/	Exclusion reason: Not along resettlement pathway;
Semere W.; Agrawal P.; Yun K.; Di Bartolo I.M.; Annamalai A.; Ross J.S.	2016	Timing of refugee health assessments may be associated with acute healthcare utilization	Journal of General Internal Medicine	31	2 SUPPL. 1	S440		Exclusion reason: conference poster/ abstract;
Shaw, Stacey A.; Karim, Hamid; Bellows, Noelle; Pillai, Veena	2019	Emotional distress among Rohingya refugees in Malaysia.	Intervention (15718883)	17	2	174-180	10.4103/INTV.INT	Exclusion reason: Not along resettlement pathway;
Shin J.; Rosenberg L.M.; Ackerman K.; Minor M.; Shah P.	2019	Trauma exposure, health status, and disease burden among minors seeking asylum in the US	Journal of General Internal Medicine	34	2 Supplement	S388	http://dx.doi.org/1	Exclusion reason: conference poster/ abstract;
Shoeb, Marwa; Weinstein, Harvey; Mollica, Richard	2007	The Harvard trauma questionnaire: adapting a cross-cultural instrument for measuring torture, trauma and posttraumatic stress disorder in Iraqi refugees.	The International journal of social psychiatry	53	5	447-63		Exclusion reason: Duplicate;
Shrestha, N M; Sharma, B; Van Ommeren, M; Regmi, S; Makaju, R; Komproe, I; Shrestha, G B; de Jong, J T	1998	Impact of torture on refugees displaced within the developing world: symptomatology among Bhutanese refugees in Nepal.	JAMA	280	5	443-8		Exclusion reason: Not along resettlement pathway;
Sierau, Susan; Schneider, Esther; Nesterko, Yuriy; von Klitzing, Kai; Glaesmer, Heide	2019	[Mental Health Problems of Unaccompanied Young Refugees in Youth Welfare Institutions].	Psychiatrische Praxis	46	3	135-140	https://dx.doi.org/	Exclusion reason: Not along resettlement pathway;
Sigvardsdotter, Erika; Nilsson, Henrik; Malm, Andreas; Tinghog, Petter; Gottvall, Maria; Vaez, Marjan; Saboonchi, Fredrik	2017	Development and Preliminary Validation of Refugee Trauma History Checklist (RTHC)-A Brief Checklist for Survey Studies.	International journal of environmental research and public health	14	10		https://dx.doi.org/	Exclusion reason: Not a screening program;
Silove, D; Sinnerbrink, I; Field, A; Manicavasagar, V; Steel, Z	1997	Anxiety, depression and PTSD in asylum-seekers: assocations with pre-migration trauma and post-migration stressors.	The British journal of psychiatry : the journal of mental science	170	0342367, b1k	351-7		Exclusion reason: Ineligible study design;
Silove, Derrick; Tay, Alvin Kuowei; Kareth, Moses; Rees, Susan	2017	The Relationship of Complex Post-traumatic Stress Disorder and Post-traumatic Stress Disorder in a Culturally Distinct, Conflict-Affected Population: A Study among West Papuan Refugees Displaced to Papua New Guinea.	Frontiers in psychiatry	8	101545006	73	https://dx.doi.org/	Exclusion reason: Not along resettlement pathway;

Authors	Published Year	Title	Journal	Volume	Issue	Pages	DOI	Notes
Slewa-Younan, Shameran; Mond, Jonathan; Bussion, Elise; Mohammad, Yaser; Uribe Guajardo, Maria Gabriela; Smith, Mitchell; Milosevic, Diana; Lujic, Sanja; Jorm, Anthony Francis	2014	Mental health literacy of resettled Iraqi refugees in Australia: knowledge about posttraumatic stress disorder and beliefs about helpfulness of interventions.	BMC psychiatry	14	100968559	320	https://dx.doi.org/	Exclusion reason: Not a screening program;
Smid, Geert E; Lensvelt-Mulders, Gerty J L M; Knipscheer, Jeroen W; Gersons, Berthold P R; Kleber, Rolf J	2011	Late-onset PTSD in unaccompanied refugee minors: exploring the predictive utility of depression and anxiety symptoms.	Journal of clinical child and adolescent psychology : the official journal for the Society of Clinical Child and Adolescent Psychology, American Psychological Association, Division 53	40	5	742-55	https://dx.doi.org/	Exclusion reason: Not a screening program;
Smith Fawzi, M C; Murphy, E; Pham, T; Lin, L; Poole, C; Mollica, R F	1997	The validity of screening for post-traumatic stress disorder and major depression among Vietnamese former political prisoners.	Acta psychiatrica Scandinavica	95	2	87-93		Exclusion reason: Not along resettlement pathway;
Sorkin D.H.; Rizzo S.; Biegler K.; Sim S.E.; Nicholas E.; Chandler M.; Ngo-Metzger Q.; Paigne K.; Nguyen D.V.; Mollica R.	2019	Novel Health Information Technology to Aid Provider Recognition and Treatment of Major Depressive Disorder and Posttraumatic Stress Disorder in Primary Care	Medical Care	57	Supplement 62	S190-S196	http://dx.doi.org/1	Exclusion reason: Not along resettlement pathway;
Sourander, A	1998	Behavior problems and traumatic events of unaccompanied refugee minors.	Child abuse & neglect	22	7	719-27		Exclusion reason: Not along resettlement pathway;
Sulaiman-Hill, Cheryl Mr; Thompson, Sandra C	2010	Selecting instruments for assessing psychological wellbeing in Afghan and Kurdish refugee groups.	BMC research notes	3	101462768	237	https://dx.doi.org/	Exclusion reason: Not along resettlement pathway;
Tay A.K.; Rees S.; Chen J.; Kareth M.; Mohsin M.; Silove D.	2015	The Refugee-Mental Health Assessment Package (R-MHAP); rationale, development and first-stage testing amongst West Papuan refugees	International Journal of Mental Health Systems	9	1	29	http://dx.doi.org/1	Exclusion reason: Not along resettlement pathway;
Tay, Alvin Kuowei; Frommer, Naomi; Hunter, Jill; Silove, Derrick; Pearson, Linda; Roque, Mehera San; Redman, Ronnit; Bryant, Richard A; Manicavasagar, Vijaya; Steel, Zachary	2015	"A mixed-method study of expert psychological evidence submitted for a cohort of asylum seekers undergoing refugee status determination in Australia": Corrigendum.	Social Science & Medicine	138		12	http://dx.doi.org/1	Exclusion reason: Duplicate;
Tay, Alvin Kuowei; Rees, Susan; Miah, Mohammed Abdul Awal; Khan, Sanjida; Badrudduza, Mohammad; Morgan, Karen; Fadil Azim, Darlina; Balasundaram, Susheela; Silove, Derrick	2019	Functional impairment as a proxy measure indicating high rates of trauma exposure, post-migration living difficulties, common mental disorders, and poor health amongst Rohingya refugees in Malaysia.	Translational psychiatry	9	1	213	https://dx.doi.org/	Exclusion reason: Not along resettlement pathway;
Teodorescu, Dinu-Stefan; Heir, Trond; Hauff, Edvard; Wentzel-Larsen, Tore; Lien, Lars	2012	Mental health problems and post-migration stress among multi-traumatized refugees attending outpatient clinics upon resettlement to Norway.	Scandinavian journal of psychology	53	4	316-32	https://dx.doi.org/	Exclusion reason: Not along resettlement pathway;
Thela, Lindokuhle; Tomita, Andrew; Maharaj, Varsha; Mhlongo, Mpho; Burns, Jonathan K	2017	Counting the cost of Afrophobia: Post-migration adaptation and mental health challenges of African refugees in South Africa.	Transcultural psychiatry	54	5-6	715-732	https://dx.doi.org/	Exclusion reason: Not along resettlement pathway;
Tibubos, Ana N; Kroger, Hannes	2020	A cross-cultural comparison of the ultrabrief mental health screeners PHQ-4 and SF-12 in Germany.	Psychological assessment	32	7	690-697	https://dx.doi.org/	Exclusion reason: Not a screening program;
Tomita, Andrew; Kandolo, Ka Muzombo; Susser, Ezra; Burns, Jonathan K	2016	Use of short messaging services to assess depressive symptoms among refugees in South Africa: Implications for social services providing mental health care in resource-poor settings.	Journal of telemedicine and telecare	22	6	369-77	https://dx.doi.org/	Exclusion reason: Not along resettlement pathway;
Uygun E.; Yildirim O.; Koseoglu A.; Erkoc S.	2018	Factors associated with PTSD in a group of Syrian refugee who applied to immigrant/refugee mental health special branch outpatient clinic	European Psychiatry	48	Supplement 1	S489	http://dx.doi.org/1	Exclusion reason: conference poster/ abstract;
Uygun, Ersin; Ilkkursun, Zeynep; Sijbrandij, Marit; Aker, A Tamer; Bryant, Richard; Cuijpers, Pim; Fuhr, Daniela C; de Graaff, Anne M; de Jong, Joop; McDaid, David; Morina, Naser; Park, A-La; Roberts, Bayard; Ventevogel, Peter; Yurtbakan, Taylan; Acarturk, Ceren; STRENGHTS consortium	2020	Protocol for a randomized controlled trial: peer-to-peer Group Problem Management Plus (PM+) for adult Syrian refugees in Turkey.	Trials	21	1	283	https://dx.doi.org/	Exclusion reason: Ineligible study design;
Vallieres, F; Ceannt, R; Daccache, F; Abou Daher, R; Sleiman, J; Gilmore, B; Byrne, S; Shevlin, M; Murphy, J; Hyland, P	2018	ICD-11 PTSD and complex PTSD amongst Syrian refugees in Lebanon: the factor structure and the clinical utility of the International Trauma Questionnaire.	Acta psychiatrica Scandinavica	138	6	547-557	https://dx.doi.org/	Exclusion reason: Not along resettlement pathway;
van Melle, Marije A; Lamkaddem, Majda; Stuiver, Martijn M; Gerritsen, Annette A M; Deville, Walter L J M; Essink-Bot, Marie-Louise	2014	Quality of primary care for resettled refugees in the Netherlands with chronic mental and physical health problems: a cross-sectional analysis of medical records and interview data.	BMC family practice	15	100967792	160	https://dx.doi.org/	Exclusion reason: Ineligible study design;
Van Ommeren, M; de Jong, J T; Sharma, B; Komproe, I; Thapa, S B; Cardena, E	2001	Psychiatric disorders among tortured Bhutanese refugees in Nepal.	Archives of general psychiatry	58	5	475-82		Exclusion reason: Not along resettlement pathway;

Authors	Published Year	Title	Journal	Volume	Issue	Pages	DOI	Notes
Van Ommeren, Mark; Sharma, Bhogendra; Sharma, Gyanendra K; Komproe, Ivan; Cardena, Etzel; de Jong, Joop T V M	2002	The relationship between somatic and PTSD symptoms among Bhutanese refugee torture survivors: examination of comorbidity with anxiety and depression.	Journal of traumatic stress	15	5	415-21		Exclusion reason: Not along resettlement pathway;
van Wyk, Sierra; Schweitzer, Robert; Brough, Mark; Vromans, Lyn; Murray, Kate	2012	A longitudinal study of mental health in refugees from Burma: the impact of therapeutic interventions.	The Australian and New Zealand journal of psychiatry	46	10	995-1003	https://dx.doi.org/	Exclusion reason: Not a screening program;
Vang, Maria Louison; Nielsen, Sabrina Brodsgaard; Auning-Hansen, Mikkel; Elklit, Ask	2019	Testing the validity of ICD-11 PTSD and CPTSD among refugees in treatment using latent class analysis.	Torture : quarterly journal on rehabilitation of torture victims and prevention of torture	29	3	27-45	https://dx.doi.org/	Exclusion reason: Not along resettlement pathway;
Veliu, Bahrie; Leathem, Janet	2017	Neuropsychological assessment of refugees: Methodological and cross-cultural barriers.	Applied neuropsychology. Adult	24	6	481-492	https://dx.doi.org/	Exclusion reason: Not a screening program;
Veronese, Guido; Pepe, Alessandro; Almurnak, Feda; Jaradah, Alaa; Hamdouna, Husam	2018	Quality of life, primary traumatisation, and positive and negative affects in primary school students in the Gaza Strip.	Lancet (London, England)	391 Suppl 2	2985213r, l0s, 00	S14	https://dx.doi.org/	Exclusion reason: Not along resettlement pathway;
Vitale, Agata; Ryde, Judy	2016	Promoting male refugees' mental health after they have been granted leave to remain (refugee status).	International Journal of Mental Health Promotion	18	2	106-125	http://dx.doi.org/1	Exclusion reason: Not a screening program;
von Overbeck Ottino, Saskia; Passini, Christina Moses; Schechter, Daniel S.	2018	Perinatal Mental Health Screening and Consultation Model for Refugee Women and Their Infants.	Zero to Three	38	4	72-74		Exclusion reason: Ineligible study design;
Vonnahme, Laura A; Lankau, Emily W; Ao, Trong; Shetty, Sharmila; Cardozo, Barbara Lopes	2015	Factors Associated with Symptoms of Depression Among Bhutanese Refugees in the United States.	Journal of immigrant and minority health	17	6	1705-14	https://dx.doi.org/	Exclusion reason: Not along resettlement pathway;
Vromans, Lyn; Schweitzer, Robert D; Brough, Mark; Correa-Velez, Ignacio; Murray, Kate; Lenette, Caroline	2019	Psychometric Properties of the Multidimensional Loss Scale with Refugee Women-at-Risk Recently Arrived in Australia.	Journal of immigrant and minority health	21	2	271-277	https://dx.doi.org/	Exclusion reason: Not a screening program;
Walg, Marco; Fink, Ewgeni; Grosmeier, Mark; Temprano, Miguel; Hapfelmeier, Gerhard	2017	[The proportion of unaccompanied refugee minors suffering from psychiatric disorders in Germany].	Zeitschrift fur Kinder- und Jugendpsychiatrie und Psychotherapie	45	1	58-68	https://dx.doi.org/	Exclusion reason: Full text unavailable;
Walker, Sophie; von Werthern, Martha; Brady, Francesca; Katona, Cornelius	2020	Mental health of forced migrants recently granted leave to remain in the United Kingdom.	The International journal of social psychiatry		gt5, 0374726	2076402093961(	https://dx.doi.org/	Exclusion reason: Not a screening program;
Weine, S M; Becker, D F; McGlashan, T H; Laub, D; Lazrove, S; Vojvoda, D; Hyman, L	1995	Psychiatric consequences of "ethnic cleansing": clinical assessments and trauma testimonies of newly resettled Bosnian refugees.	The American journal of psychiatry	152	4	536-42		Exclusion reason: Not a screening program;
Weine, Stevan M; Raina, Dheeraj; Zhubi, Merita; Delesi, Mejreme; Huseni, Dzana; Feetham, Suzanne; Kulauzovic, Yasmina; Mermelstein, Robin; Campbell, Richard T; Rolland, John; Pavkovic, Ivan	2003	The TAFES multi-family group intervention for Kosovar refugees: a feasibility study.	The Journal of nervous and mental disease	191	2	100-7		Exclusion reason: Not a screening program;
Westermeyer, Joseph J; Campbell, Robyn; Lien, Rebecca; Spring, Marline; Johnson, David R; Butcher, James; Hyland, Jacqueline; Thuras, Paul; Jaranson, James M	2010	HADStress: a somatic symptom screen for posttraumatic stress among Somali refugees.	Psychiatric services (Washington, D.C.)	61	11	1132-7	https://dx.doi.org/	Exclusion reason: Not along resettlement pathway;
Willey, Suzanne M; Blackmore, Rebecca P; Gibson-Helm, Melanie E; Ali, Razia; Boyd, Leanne M; McBride, Jacqueline; Boyle, Jacqueline A	2020	"If you don't ask ... you don't tell": Refugee women's perspectives on perinatal mental health screening.	Women and birth : journal of the Australian College of Midwives	33	5	e429-e437	https://dx.doi.org/	Exclusion reason: Not along resettlement pathway;
Wind, Tim R; van der Aa, Niels; Knipscheer, Jeroen; de la Rie, Simone	2017	"The assessment of psychopathology among traumatized refugees: Measurement invariance of the Harvard Trauma Questionnaire and the Hopkins Symptom Checklist-25 across five linguistic groups": Corrigendum.	European Journal of Psychotraumatology	8	Suppl 2	No-Specified	http://dx.doi.org/1	Exclusion reason: Not along resettlement pathway;
Young P.	2014	Mental health screening and outcome measures in immigration detention	Australian and New Zealand Journal of Psychiatry	48	SUPPL. 1	92-93	http://dx.doi.org/1	Exclusion reason: conference poster/ abstract;
